# Supplementary material for: Health condition at first fit note and number of fit notes: a longitudinal study of primary care records in south London
Source: BMJ Open. 2021 Mar 26;11(3):e043889. doi: 10.1136/bmjopen-2020-043889 (PMC8006821; doi:10.1136/bmjopen-2020-043889)
Supplement: Supplementary data [file bmjopen-2020-043889supp001.pdf]

Supplementary Figure 1 Classification of conditions into sub-groups, groups and categories

**Categories**	**Groups**	**Sub-groups**
Non-Mental Health	Injury*	Injury*
	Infection*	Infection*
	Surgery*	Minor Surgery*<br>Major Surgery*
	Fatigue/Insomnia	Insomnia<br>Fatigue
	Obstetrics	Obstetrics
	Musculoskeletal	Musculoskeletal
	Physical Health	Cancer<br>Neurology<br>Haematology<br>Obesity<br>Test<br>Cardiovascular<br>Gastroenterology<br>Gynaecology<br>Dermatology<br>Respiratory<br>Physical Health Treatment<br>Genitourinary Medicine/Urology<br>Allergy<br>Risk factor<br>Ear, Nose, Throat and Dental<br>Renal<br>Diabetes<br>Systemic Illness<br>Physical symptom<br>Wound
	Other	Other Physical Health
Mental Health	Mental Illness	Common Mental Disorders<br>Severe Mental Illness<br>Drug and Alcohol<br>Mental Health Treatment<br>Other Mental Health Problem**
	External Stressor	Bereavement<br>External stressor
	Stressed	Stressed

*Injury, infection and surgery were at the top of the two-tier hierarchy of groupings, as an alternative to assigning injuries, infections and surgery to their system groups.
** Other mental health problem includes self-harm, developmental disorders, personality disorder
