# Supplementary material for: Health condition at first fit note and number of fit notes: a longitudinal study of primary care records in south London
Source: BMJ Open. 2021 Mar 26;11(3):e043889. doi: 10.1136/bmjopen-2020-043889 (PMC8006821; doi:10.1136/bmjopen-2020-043889)
Supplement: Supplementary data [file bmjopen-2020-043889supp002.pdf]

SUPPLEMENTARY MATERIAL TABLES

Table 1 Predicted number of fit notes by condition type

Condition sub-group	Number (%)	Predicted number of fit notes (NFN)	Predicted NFN adj*	Predicted NFN adj**
Drug and alcohol	584 (1.4)	3.9 (3.6-4.3)	4.2 (3.8-4.6)	4.5 (4.1-4.8)
Other Mental Health Problem	294 (0.7)	2.7 (2.3-3.0)	3.7 (3.2-4.2)	3.6 (3.1-4.0)
Common Mental Disorders	4,393(10.8)	2.7 (2.5-2.9)	3.2 (3.1-3.4)	3.2 (3.1-3.3)
Severe Mental Illness	374 (0.9)	2.7 (2.5-3.0)	3.0 (2.8-3.3)	2.6 (2.4-2.8)
Neurology	640 (1.6)	2.5 (2.2-2.8)	2.8 (2.5-3.1)	2.8 (2.5-3.1)
Haematology	134 (0.3)	2.5 (2.1-2.9)	2.8 (2.3-3.3)	3.2 (2.6-3.8)
Obesity	74 (0.2)	2.7 (2.0-3.3)	2.7 (2.0-3.4)	2.4 (1.8-2.9)
Insomnia	174 (0.4)	2.2 (1.8-2.7)	2.6 (2.0-3.2)	2.7 (2.1-3.3)
Mental Health Treatment	192 (0.5)	2.3 (1.7-2.8)	2.6 (2.0-3.1)	2.4 (1.9-2.9)
Cancer	239 (0.6)	2.5 (2.3-2.8)	2.5 (2.2-2.7)	2.5 (2.2-2.7)
Stressed	1,039 (2.6)	2.1 (1.9-2.3)	2.5 (2.2-2.7)	2.7 (2.5-2.9)
Diabetes	58 (0.1)	2.8 (2.0-3.6)	2.5 (1.8-3.2)	2.1 (1.5-2.7)
Systemic Illness	1,028 (2.5)	2.6 (2.3-2.8)	2.4 (2.2-2.6)	2.1 (1.9-2.3)
Physical symptom	1,439 (3.5)	2.2 (2.0-2.4)	2.4 (2.2-2.5)	2.5 (2.3-2.6)
Other	326 (0.8)	2.2 (1.9-2.5)	2.4 (2.1-2.6)	2.5 (2.2-2.7)
External stressor	349 (0.9)	2.1 (1.8-2.5)	2.4 (2.0-2.7)	2.6 (2.2-2.9)
Musculoskeletal	8,288 (20.4)	2.3 (2.1 2.4)	2.3 (2.2-2.5)	2.6 (2.4-2.7)
Bereavement	495 (1.2)	2.2 (1.9-2.5)	2.3 (2.1-2.6)	2.5 (2.3-2.8)
Test	26 (0.1)	2.7 (1.7-3.7)	2.3 (1.6-3.1)	2.3 (1.6-3.0)
Cardiovascular	1,234 (3.0)	2.5 (2.2-2.7)	2.2 (2.1-2.4)	2.2 (2.0-2.3)
Gastroenterology	2,241 (5.5)	2.0 (1.8-2.1)	2.2 (2.1-2.4)	2.4 (2.3-2.6)
Gynaecology	768 (1.9)	1.9 (1.7-2.0)	2.2 (2.0-2.3)	2.4 (2.3-2.6)
Dermatology	1,129 (2.8)	1.9 (1.6-2.1)	2.2 (1.9-2.4)	2.3 (2.1-2.6)
Respiratory	1,663 (4.1)	2.0 (1.8-2.2)	2.1 (2.0-2.3)	2.0 (1.9-2.2)
Injury	2,183 (5.4)	1.8 (1.6-2.0)	2.1 (1.9-2.3)	2.4 (2.2-2.5)
Fatigue	367 (0.9)	1.8 (1.5-2.1)	2.1 (1.8-2.4)	2.3 (2.0-2.6)
Physical Health Treatment	13 (0.0)	1.9 (1.0-2.9)	2.1 (1.0-3.2)	2.1 (0.8-3.3)
Genitourinary Medicine/Urology	481 (1.2)	2.0 (1.7-2.3)	2.0 (1.8-2.3)	2.1 (1.9-2.4)
Obstetrics	1,371 (3.4)	1.4 (1.3-1.6)	2.0 (1.8-2.2)	2.2 (2.0-2.5)
Ear, Nose, Throat and Dental	1,016 (2.5)	1.8 (1.6-2.1)	2.0 (1.7-2.2)	2.1 (1.9-2.4)
Wound	562 (1.4)	1.8 (1.6-2.0)	1.9 (1.8-2.1)	2.1 (1.9-2.3)
Infection	6,727 (16.5)	1.5 (1.4-1.7)	1.8 (1.7-1.9)	1.9 (1.8-2.0)
Risk factor	33 (0.1)	1.8 (1.1-2.5)	1.8 (1.2-2.3)	1.8 (1.2-2.4)
Minor Surgery	232 (0.6)	1.6 (1.3-1.9)	1.7 (1.4-2.0)	1.9 (1.5-2.3)
Allergy	88 (0.2)	1.4 (1.0-1.9)	1.7 (1.2-2.2)	1.9 (1.3-2.4)
Major Surgery	397 (1.0)	1.6 (1.3-1.8)	1.6 (1.4-1.9)	1.8 (1.5-2.0)
Renal	47 (0.1)	1.6 (0.8-2.3)	1.6 (0.9-2.4)	1.9 (1.0-2.7)

*adjusted for age, gender, ethnicity and deprivation

**adjusted for age, gender, ethnicity, deprivation and number of long term condition

Table 2 Example of subgroup codes:

Drug and Alcohol Codes:
[RFC] Drug/ substance abuse<br>[RFC] Substance abuse<br>[X]Alcohol addiction<br>[X]Drug addiction - cannabis<br>[X]Heroin addiction<br>[X]Men & behav disorder multiple drug use/psychoactive subst<br>[X]Mental and behav dis due to use alcohol: dependence syndr<br>[X]Mental and behav dis due to use cannabinoids: harmful use<br>[X]Mental and behav dis due to use of alcohol: harmful use<br>[X]Mental and behav dis due to use of cocaine: harmful use<br>[X]Mental and behav dis due to use of opioids: harmful use<br>[X]Mental and behav dis due to use opioids: dependence syndr<br>[X]Mental behav disorders due use crack cocaine: harmful use<br>Alcohol dependence syndrome<br>Alcohol dependence syndrome NOS<br>Alcohol misuse - enhanced service completed<br>Alcohol problem drinking<br>Alcohol withdrawal syndrome<br>Alcoholism<br>Analgesic overuse headache<br>Benzodiazepine dependence<br>Cannabis dependence, continuous<br>Cannabis dependence, unspecified<br>Cannabis type drug dependence<br>Chronic alcoholism<br>Chronic alcoholism in remission<br>Cocaine dependence, episodic<br>Cocaine dependence, unspecified<br>Cocaine drug dependence NOS<br>Cocaine type drug dependence<br>Diazepam dependence<br>Drug addiction<br>Drug addiction therapy<br>Drug addictn therap-methadone<br>Drug dependence<br>Drug dependence NOS<br>Drug dependence therapy<br>Drug seeking behaviour<br>Drug withdrawal syndrome<br>Drunkenness NOS<br>Hangover (alcohol)<br>Harmful alcohol use<br>Hazardous alcohol use<br>Heavy drinker - 7-9u/day<br>Heroin dependence<br>Intoxication - alcohol<br>Methadone dependence<br>Misuse of drugs NOS<br>Morphine dependence<br>Nondependent alcohol abuse<br>Nondependent cannabis abuse<br>Opioid drug dependence NOS<br>Opioid type drug dependence<br>Refer to drugs service<br>Very heavy drinker - >9u/day
