# Supplementary material for: Health condition at first fit note and number of fit notes: a longitudinal study of primary care records in south London
Source: BMJ Open. 2021 Mar 26;11(3):e043889. doi: 10.1136/bmjopen-2020-043889 (PMC8006821; doi:10.1136/bmjopen-2020-043889)
Supplement: Supplementary data [file bmjopen-2020-043889supp003.pdf]

Supplementary Figure 2: Percentage of patients with 1, 2, 3 or 4+ fit notes by symptom at first fit note

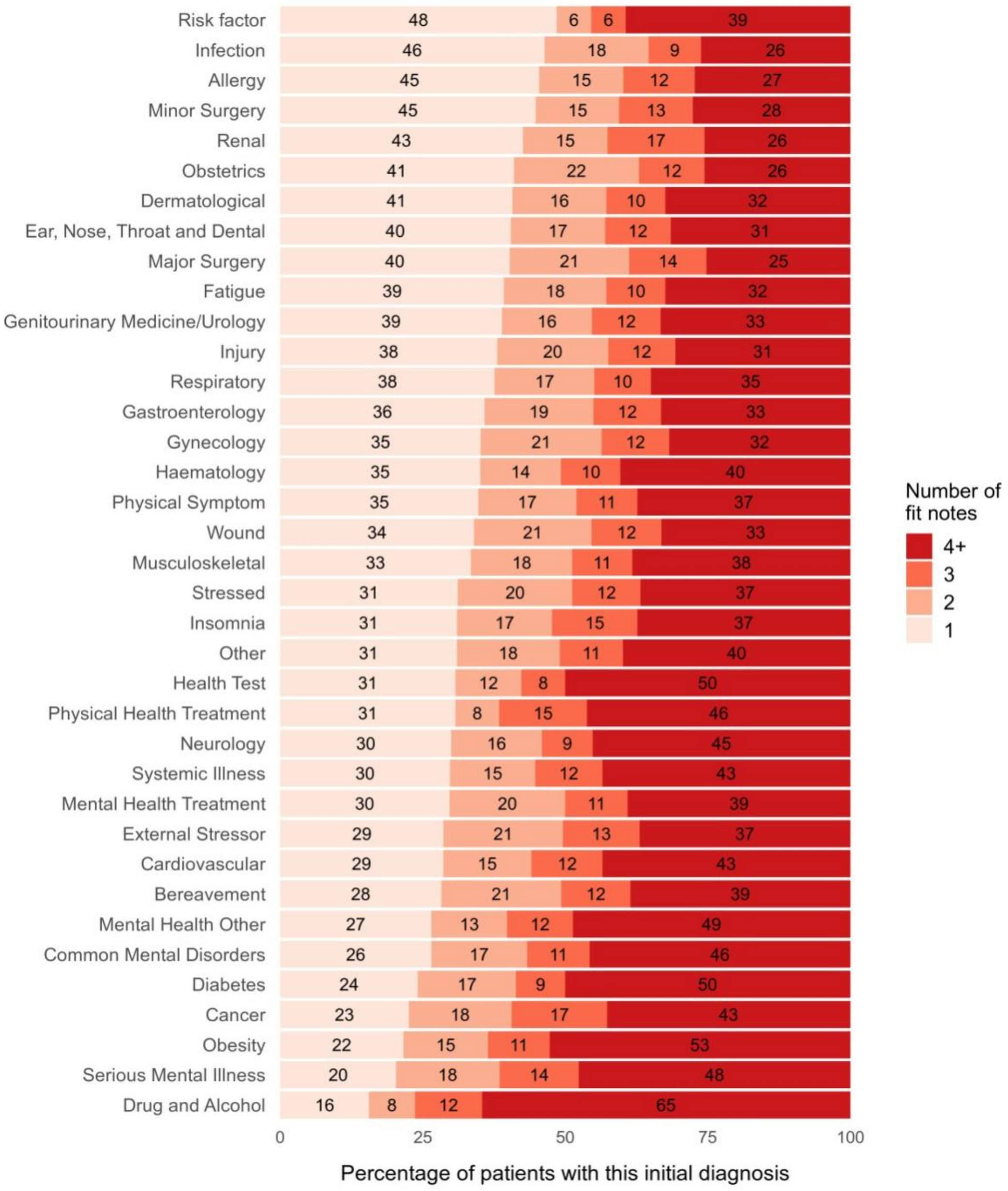

Data source: Lambeth Datanet. Sample size: 40698 patients with fit notes aged 16-60, registered with a Lambeth GP between 1st Jan 2014 and 30th April 2017
