# Supplementary material for: Health condition at first fit note and number of fit notes: a longitudinal study of primary care records in south London
Source: BMJ Open. 2021 Mar 26;11(3):e043889. doi: 10.1136/bmjopen-2020-043889 (PMC8006821; doi:10.1136/bmjopen-2020-043889)
Supplement: Author's manuscript [file bmjopen-2020-043889.draft_revisions.pdf]

BMJ Open

BMJ Open is committed to open peer review. As part of this commitment we make the peer review history of every article we publish publicly available.

When an article is published we post the peer reviewers' comments and the authors' responses online. We also post the versions of the paper that were used during peer review. These are the versions that the peer review comments apply to.

The versions of the paper that follow are the versions that were submitted during the peer review process. They are not the versions of record or the final published versions. They should not be cited or distributed as the published version of this manuscript.

BMJ Open is an open access journal and the full, final, typeset and author-corrected version of record of the manuscript is available on our site with no access controls, subscription charges or pay-per-view fees (http://bmjopen.bmj.com).

If you have any questions on BMJ Open's open peer review process please email info.bmjopen@bmj.com

BMJ Open

Health condition at first fit note and number of fit notes: evidence from a large primary care sample in south London

Journal:	*BMJ Open*
Manuscript ID	bmjopen-2020-043889
Article Type:	Original research
Date Submitted by the Author:	18-Aug-2020
Complete List of Authors:	Dorrington, Sarah; King's College London,<br>Carr, Ewan ; King's College London,<br>Polling, C; King's College London, Psychological Medicine, Institute of Psychiatry, Psychology and Neuroscience<br>Stevelink, Sharon; King's College London, Department of Psychological Medicine<br>Ashworth, Mark; King's College London, UK, School of Population Health & Environmental Sciences<br>Roberts , Emmert; Institute of Psychiatry Psychology and Neuroscience, National Addiction Centre; South London and Maudsley NHS Foundation Trust,<br>Broadbent, Matthew; South London and Maudsley NHS Foundation Trust,<br>HATCH, STEPHANI; INSTITUTE OF PSYCHIATRY, KINGS COLLEGE LONDON,<br>MADAN, IRA; GUYS AND ST THOMAS NHS FOUNDATION TRUST,<br>Hotopf, Matthew; King's College London (Institute of Psychiatry),
Keywords:	PRIMARY CARE, PUBLIC HEALTH, OCCUPATIONAL & INDUSTRIAL MEDICINE, Health policy < HEALTH SERVICES ADMINISTRATION & MANAGEMENT

SCHOLARONE™
Manuscripts

BMJ

I, the Submitting Author has the right to grant and does grant on behalf of all authors of the Work (as defined in the below author licence), an exclusive licence and/or a non-exclusive licence for contributions from authors who are: i) UK Crown employees; ii) where BMJ has agreed a CC-BY licence shall apply, and/or iii) in accordance with the terms applicable for US Federal Government officers or employees acting as part of their official duties; on a worldwide, perpetual, irrevocable, royalty-free basis to BMJ Publishing Group Ltd ("BMJ") its licensees and where the relevant Journal is co-owned by BMJ to the co-owners of the Journal, to publish the Work in this journal and any other BMJ products and to exploit all rights, as set out in our licence.

The Submitting Author accepts and understands that any supply made under these terms is made by BMJ to the Submitting Author unless you are acting as an employee on behalf of your employer or a postgraduate student of an affiliated institution which is paying any applicable article publishing charge ("APC") for Open Access articles. Where the Submitting Author wishes to make the Work available on an Open Access basis (and intends to pay the relevant APC), the terms of reuse of such Open Access shall be governed by a Creative Commons licence – details of these licences and which Creative Commons licence will apply to this Work are set out in our licence referred to above.

Other than as permitted in any relevant BMJ Author's Self Archiving Policies, I confirm this Work has not been accepted for publication elsewhere, is not being considered for publication elsewhere and does not duplicate material already published. I confirm all authors consent to publication of this Work and authorise the granting of this licence.

For peer review only

Health condition at first fit note and number of fit notes: evidence from a large primary care sample in south London

Sarah Dorrington (1,2)*

Ewan Carr (1)

Catherine Polling (1,2)

Sharon A.M. Stevelink (1)

Mark Ashworth (3)

Emmert Roberts (1,2)

Matthew Broadbent (2)

Stephani L Hatch (1)

Ira Madan (4)

Matthew Hotopf (1,2)

(1) Institute of Psychiatry, Psychology & Neuroscience King's College London, 16 De Crespigny Park, London SE5 8AF

(2) South London and Maudsley NHS Foundation Trust, London, UK

(3) School of Population Health and Environmental Sciences, King's College London, Guy's Campus, Addison House, London SE1 1UL

(4) Department of Occupational Health, Guy's and St Thomas' NHS Foundation Trust, London, UK

Word count: 3508

*Corresponding author.

3rd Floor, Institute of Psychiatry, Psychology and Neuroscience, De Crespigny Park, London SE5 8AF; Sarah.dorrington@kcl.ac.uk; 02078485817.

For peer review only

We used the STROBE cohort checklist when writing our report.

Funding statement: This paper represents independent research part funded by the Royal College of Psychiatrists' Donald Dean Fellowship and the National Institute for Health Research (NIHR) Biomedical Research Centre at South London and Maudsley NHS Foundation Trust and King's College London (IS-BRC-1215-20018). The views expressed are those of the authors and not necessarily those of the Royal College of Psychiatrists, the NHS, the NIHR or the Department of Health and NIHR Maudsley Biomedical Research Centre, South London and Maudsley NHS Foundation Trust, London, UK.

Acknowledgements: Particular thanks to Megan Pritchard and Amelia Jewell at the NIHR Maudsley Biomedical Research Centre for their support with this study.

Conflict of interests: Professor Matthew Hotopf receives funding from Janssen as part of the RADAR-CNS consortium, which includes a project on depression. He is a principal investigator of RADAR-CNS, a pre-competitive public private partnership co-funded by Innovative Medicines Initiative (European Commission) and European Federation of Pharmaceutical Industries and Associations (EFPIA). He has also been an independent expert witness in group litigations instructed by claimants against pharmaceutical companies for alleged harmful effects of their products.
Authors have no other conflict of interest to declare.

Key words: fit note, health policy, primary care, public health, occupational health

Abstract

Objectives: The fit note, introduced in the UK in 2010, was designed to reduce long-term sickness absence. Previous fit note research has relied on extracting diagnoses directly from fit notes, rather than extracting information from clinical records. In this paper we extract information from clinical records to explore demographic factors and conditions associated with number of fit notes issued.

Design: This is a longitudinal study of clinical data. We analysed individual-level anonymised data from general practitioner consultations, including demographic information and condition recorded at first fit note. The latter encompassed diagnoses, individual symptoms and psychosocial issues.

For peer review only

Setting: A database called Lambeth DataNet, containing electronic clinical records on 326,415 adults (ages 16-60) from all 45 general practices within the London borough of Lambeth from 1st Jan 2014 to 30th April 2017.

Participants: Our analytical sample contained 40,698 people with a condition recorded at first fit note.

Primary outcome measure: Predicted number of fit notes

Results: Of all studied diagnostic groups, mental illness had the highest number of fit notes (n=3.3; 95% CI 3.1,3.4) after controlling for demographic factors and long-term conditions. The highest predicted number of fit notes for any condition subgroup was among patients presenting for drug and/or alcohol misuse (NFN = 4.5; 95% CI 4.1, 4.8).

Conclusions: For the first time, we show drug and/or alcohol misuse at first fit note are associated with the highest number of fit notes. Research is needed to understand the trajectories of individuals at highest risk of long-term sickness absence, in particular, people presenting with drug and/or alcohol misuse.

Strengths and limitations of this study

- This is the largest study to analyse fit note use and clinical information from GP records at point of first fit note, using individual-level clinical data; a major strength of using clinical records data is that they contain more detail on condition type than information reported on the fit note which previous research has relied on.
- The study was conducted in a single geographical area for which we had GP data; this had the benefit of providing information on virtually all individuals seeking help within that population, but it had the disadvantage of a possible loss of generalisability, and our findings should be tested in other areas.
- We do not have access to the information written on the fit note to compare it with the information recorded in clinical notes, and we do not know the length of time that each fit note was prescribed for.
- Each of the condition groups and subgroups encompasses a very wide variety of disorders.
- There is additional information that would have been extremely useful, such as nature of employment, educational level, occupational status or benefit use, which were not available in this dataset.

For peer review only

Introduction

In the UK long term sickness absence is a major driver of inequalities, leading to social exclusion and adverse health outcomes[1]. The impact of long term sickness absence is usually expressed in financial terms, with an estimated £15bn lost to the economy in the year 2017/18[2]. In 2008, a government report suggested that the process of sickness certification by general practitioners (GPs) was a contributor to the problem of long-term sickness absence, and recommended the introduction of a new form of sickness certification: the fit note[2].

The sick note, used to certify episodes of sickness absence of over 7 days, was replaced by the fit note in 2010[3]. The fit note was designed to reduce long term sickness absence by replacing the sick note's binary 'fit' versus 'not fit to work', with the addition of a third option, 'maybe fit'[2]. 'Not fit for work' and the new 'maybe fit' option both provide evidence of sickness to an employer and can be used in the administration of sickness benefits. The fit note also includes space for GPs to recommend work adjustments for employees, to encourage employers to adapt to the health needs of workers[4]. The digitisation of the fit note in 2012 created the opportunity for researchers and policy makers to describe patterns of fit note use in the UK population[5-7].

A single fit note following an injury or illness is a common occurrence. A study found that 60% of all fit notes issued in the UK from 2011-13 were single fit notes[8]. In contrast, repeated fit notes are rarer and suggest a more serious injury or illness, or other reasons for not recovering, such as multimorbidity, illness complications or barriers to return to work. International research suggests that a large proportion of workers presenting for sickness certification have multimorbidity[9] (Dorrington et al. *in press* Psychological Medicine); yet, the extent of multimorbidity is rarely captured on the fit note itself, even though there is no limit to the number of conditions that can be recorded. Even when information about multimorbidity is provided on the fit note, it is often excluded from analysis[8,10].

In the majority of fit note research, the reason for sickness absence is taken directly from the fit note, where information is written for the worker, employer and/or the Department of Work and Pensions[11,12]. There are limitations to this approach. Firstly, half of all fit notes do not provide adequate information for the reason for sickness absence to be coded[13], and secondly, information on the fit note may differ from information in the clinical notes, for reasons such as individual confidentiality or clinicians' preference[12,14,15].

Research to date has found multiple fit note use to be associated with mental illness and stress-related problems, higher levels of deprivation, minority ethnic groups, and female gender[16-18].

For peer review only

Mental disorders are the single most common reason for a fit note, making up over 30% of fit notes nationally (NHS Digital 2014-2017)[5], and are strongly associated with multimorbidity[19].

In this paper we extract conditions recorded in clinical records at the point of first fit note receipt from Read codes recorded by general practitioners. Conditions include diagnoses, individual symptoms, psychosocial issues and codes related to the healthcare provided. We explore the relationship between variables known to be associated with fit note receipt and number of fit notes, anticipating that the number of fit notes over time would be higher among individuals with mental disorders, those with multimorbidity, women, those living in areas of greater deprivation, older age groups, and minority ethnic groups. We will test for a multiplier effect of mental health and physical co-morbidity on number of fit notes. We expect demographic associations to be partially explained by long-term conditions and that individuals with infection, injury and obstetric conditions will tend to have a fewer fit notes.

Methods

Data

Prospective longitudinal data for 326,415 patients were extracted from electronic clinical records using a database of primary care providers in the London Borough of Lambeth, Lambeth DataNet (LDN)[18]. The London Borough of Lambeth, home to a population of just over 318,000, is the 22nd most deprived local authority in England[20] and has the 4th highest level of income inequality of any borough in London[21]. Lambeth contains a young and ethnically diverse population, including large Portuguese, South American and Black populations[22]. Around 150 different languages are spoken and around 17,000 people (6% of the population) speak a main language other than English[20]. Thirty-eight percent of the population was born outside of the UK[20]. The number of individuals registered on LDN at any one time (n=405,000) exceeds the total Lambeth population recorded in the Census, due to the cross-boundary registration of individuals not living in Lambeth, GP list inflation[23], and the temporary residence of students in the Lambeth area from surrounding universities. We analysed records covering a period of 40 months from 1st January 2014 to 30th April 2017. Data were extracted in May 2017 from the primary care clinical record system, EMIS Web. We restricted the sample to adults age 16-60 years to exclude exits from the workforce due to reaching statutory pension age (ages 60-65 years), which was not measured in our sample.

Demographic variables

Information on individuals' first recorded sex, age at the beginning of window, ethnicity and lower super output area (LSOA) of residence (an area covering an average population of 1,722[24]) were

For peer review only

extracted from the GP records. Ethnicity was coded using seven subcategories: Asian, Black African, Black Caribbean, Black Other, Mixed, Other and White). This uses the five broad groups used in the 2011 census, with the Black group split into sub-groups, following previous research within South London suggesting differences in mental health outcomes between Black Caribbean and Black African groups[25 26]. Deprivation was measured using the Index of Multiple Deprivation (IMD) 2015 rank of their LSOA of residence. IMD scores were divided into quintiles, based on deprivation scores in Lambeth[27].

Fit note

Date of first fit note was derived for each participant based on digital fit notes issued between 1st January 2014 to 30th April 2016. We excluded fit notes issued before this period because although digital fit notes were introduced in 2012, they were not in widespread use until 2014. Due to the lack of information on prior sickness certification (before January 2014) we could not determine whether a 'first fit note' during the study period represented the individual's first ever sickness certification. Number of fit notes are measured as the total number of fit notes received in the study window.

Presenting condition at first fit note

There were a wide variety of conditions (recorded using Read codes) in the electronic GP record when a first fit note was issued. These were split into two broad categories of "Mental Health" and "Non-Mental Health", containing 11 broad groups comprising 37 subgroups. The creation of 11 groups was based on a pragmatic mix of the size of the groups, the way diagnoses are grouped in ICD-10 and past fit note research[28 10 29]. The three Mental Health groups were: mental disorder, stress and specific external stressors (which includes bereavement and relationship breakdown) and the eight Non-Mental Health groups were musculoskeletal, infection, surgery, obstetrics, injury, physical health, fatigue, and a group for 'other' conditions that could not easily be categorised into one of the other groups (e.g. administrative codes). The broad 'physical health' group was created for individual symptoms that could not be attributed to a single system or group of diagnoses. We defined 37 diagnostic subgroups (Supplementary Figure 1 and Supplementary Table 1). Where a condition fit into more than one diagnostic group, we used a hierarchy to allocate codes ensuring each condition was allocated to a single diagnostic subgroup (see Supplementary Figure 1).

Long term conditions and pain

Presence of long-term conditions was assessed in addition to condition at first fit note, using the Quality and Outcomes Framework (QOF), an annual reward and incentive programme for all GP practices in England. QOF measures have been shown to underestimate prevalence of disorders in the general population due to the reliance on the GP for a diagnosis[30]. QOF diagnosis is

For peer review only

therefore a specific, but not sensitive measure[31] with a low false-positive but high false-negative rates. In this paper 'long term condition' comprises 15 QOF[32] conditions: depression, epilepsy, diabetes mellitus (DM), coronary heart disease (CHD), chronic obstructive pulmonary disorder (COPD), cancer (non-specified), atrial fibrillation (AF), heart failure (HF), stroke, rheumatoid arthritis, obesity, severe mental illness (SMI; schizophrenia, bipolar disorder), learning disability, hypertension and asthma. QOF conditions excluded from our analysis were smoking (not a long-term condition), palliative care, osteoporosis, dementia and chronic kidney disease (due to the small numbers with these diagnosis in the age group under study). In addition, chronic pain[33] was derived based on receipt (yes/no) of any prescriptions listed in British National Formulary medication chapters 4.7.2 or 4.7.3 (opioid analgesics and neuropathic pain medication) with repeat, repeat dispensed or automatic issue type.

Statistical analysis

We undertook a descriptive analysis of demographic variables (age, gender, ethnicity and deprivation), long-term conditions and conditions at first fit note. To assess associations between conditions and fit note counts we used zero-truncated negative binomial regression models. A truncated model was used because all individuals in our sample had at least one fit note to ensure predictions were made on the range $[1, \infty)$ rather than $[0, \infty)$. A negative binomial model was used to account for overdispersion in the number of fit notes. To account for clustering of individuals within GP practices, standard errors were adjusted using a sandwich estimator[34]. Models are presented in terms of model predicted number of fit notes because this is the most clinically relevant outcome. The predicted number of fit notes was calculated for each group, holding other variables to their sample mean values. In model 1 we adjust for demographic variables: age, gender, ethnicity and deprivation, and in model 2 we adjust for demographic variables and additionally the number of long-term conditions. We did a full case analysis, described in detail in a recent publication[18].

Results

Our analytical sample included 40,698 patients with at least one condition code recorded at first fit note, having excluded 284,913 patients without any fit notes, and 804 people with undefined reasons for fit note use, these included administrative codes reported in the clinical record at point of first fit note. Of 40,698 patients, 86% of individuals had one condition code and 14% of people had more than one condition recorded in the clinical notes at first fit note. The number of conditions types varied by age, with 13% of 16-40 year-olds having multiple condition types compared with 17% among 51-60 year-olds ($p<0.001$). The largest group of conditions was the

For peer review only

physical health group (31% of all conditions), followed by musculoskeletal (21%), infection (17%) and mental illness (14%).

Table 1 shows that the number of fit notes received varied by age, ethnicity and deprivation, but were similar among women and men. Older age, high deprivation, Black Caribbean, Black Other and Mixed ethnic groups were strongly associated with a higher number of fit notes. Twice as many individuals in the oldest age group (51-60 years) received 4+ fit notes compared to those aged 20-25 years. The percentage of individuals receiving 4+ fit notes was higher among Black Caribbean and Black Other individuals (47% and 45%, compared with 34% or less among Other, Asian and White individuals), and for individuals living in more deprived areas (compared to those living in less deprived areas). These differences remained after adjustment for long-term conditions.

Table 2 and Figure 1 show the number of fit notes by condition at first fit note. Mental illness, stress and musculoskeletal conditions were associated with the highest number of fit notes, after adjusting for demographic factors and long-term conditions. Infection and surgery were associated with the lowest numbers of fit notes. After adjustment for demographic factors and long-term conditions, of the 37 subgroups the highest predicted number of fit notes were for drug and/or alcohol misuse, common mental disorders, and severe mental illness (Table 2, Supplementary Table 1 and Figure 2).

Patients with comorbid mental and physical health conditions had a higher predicted number of fit notes than patients with either mental health or physical health conditions alone. The predicted number of fit notes increased across all condition groups when coupled with a mental health condition but interaction effects reached statistical significance only for mental health conditions co-morbid with infection at first fit note (predicted fit notes for infection 1.8 [1.7,1.8] versus co-morbid infection and mental health condition 3.2 [2.4,4.0] (chi2 3.7 (1) p=0.05), these results are in the text only.

We found no associations between number of condition types and number of fit notes, but we found a higher number of fit notes amongst individuals with mental health conditions than those with non-mental health conditions, independent of the number of condition types recorded at first fit note.

Discussion

We show for the first time that individuals with drug and/or alcohol misuse have the highest number of fit notes of any condition at first fit note. We are aware of one study identifying alcohol

For peer review only

and drug misuse recorded on fit notes which found that alcohol and drug misuse was more common in the most socially deprived areas, making up 9% of fit notes for mild-moderate mental health disorders, compared to 1% in the least deprived areas[35]. We anticipate that such analyses of drug and/or alcohol misuse recorded on fit notes (rather than in clinical records as in this study) are likely to lead to an underestimation, because GPs writing fit notes are aware that if they record substance misuse disorders directly on the fit note, it will be seen by the individual's employer[15].

Across the 11 main groups, our findings were consistent with previous studies; mental illness at first fit note was associated with the highest number of fit notes, followed by 'feeling stressed' and 'external stressors'[18 36]. In contrast, infection and minor surgery were associated with the smallest number of fit notes[8]. Evidence suggests that co-existing mental and physical conditions are associated with poorer clinical and functional outcomes and lower quality of life, than physical conditions alone[19 37-47]. We found that the combination of mental and physical disorders at first fit note increased NFN significantly compared to physical conditions alone, but we did not find a "multiplier effect" of mental health across physical conditions. However, amongst people who presented with infection and mental health conditions at first fit note, the combination doubled the predicted number of fit notes of infection (p=0.05).

Older individuals, people living in deprived areas and those in Black Other, Black Caribbean, and Mixed groups continue to receive on average a higher number of fit notes in the final fully adjusted model. This remaining association is likely to be explained by exposure to a multitude of disadvantages that we have not been able to measure, such as long term conditions not captured by the QOF, occupational disadvantages such as precarity, high risk occupations (e.g. construction, health and social care)[10] and work place discrimination[48]. Although women are more likely to receive a first fit note, we find that they then received a similar number of fit notes to men.

The largest study of fit notes to date is national reporting on fit notes issued, published by NHS Digital[7]. At first glance, compared to NHS Digital, there appears to be an underrepresentation of mental health in our sample, despite being a population with a high prevalence of mental health problems[49]. The lower prevalence of fit notes for mental health in our sample compared to previous studies is expected because we have extracted diagnosis at first fit note, rather than diagnosis on all fit notes, of which mental health takes up a larger proportion due to the association between mental health conditions and multiple fit note use[13]. Secondly, we know that 1.7-9.6% of non-mental health diagnosis will change to mental health conditions on future fit notes, which we have not captured in our study[8].

For peer review only

In contrast to mental health conditions, infections make up a much higher proportion of first fit notes in our sample than total fit note samples (16.5% at first fit note vs 3.5% of NHS Digital sample). Infection is associated with lower number of fit notes and younger populations, so we expect to see a higher proportion of fit notes for infection at first fit note than found in the NHS digital sample which includes diagnosis on all fit notes, not just the first. In addition, the categorisation of condition types in our study is likely to explain some of the difference: we have categorised conditions using a hierarchy of condition groups, infections were grouped together instead of added to their corresponding system group[28]. Our findings are in keeping with the labour force survey in which 'minor illnesses', including infections, were a large contributor to sickness absence[50].

The 'drug and/or alcohol misuse' subgroup in our study represents a wide spectrum of conditions, including harmful use and dependence on multiple different substances (Supplementary Table 2). Substance misuse, in particular alcohol misuse, is common and associated with absenteeism[51], and higher risk drinking profiles in early adulthood are an important predictor of receiving a disability pension later in life.[52]. The Alcohol Use Disorders Identification Test (AUDIT) used in the Adult Psychiatric Morbidity Survey 2014 found that 20% of adults in England drink at hazardous levels and above (AUDIT $\geq$ 8). White men and women are more likely to drink at hazardous, and harmful levels than Black, Asian and minority ethnic groups. Some workforce groups, such as doctors, and male dominated industries such as the military and construction, have been identified as having particularly high levels of alcohol misuse[53-55]. Despite high levels of physical and mental comorbidity, workers with drug and/or alcohol misuse often present late, and struggle to access health services[56]. In addition, in recent years funding for addiction services has been drastically cut in England, and addiction budgets removed from ringfencing protections within the NHS[57 58]. The findings suggest a strong case for employment based treatment services such as the successful in-house Drug and Alcohol Treatment Service developed by Transport for London[59], a major employer in Lambeth.

This study suggests that communities at highest risk of insecure work, and workers with mental health conditions, particularly drug and/or alcohol misuse, experience major barriers to work. Countries in which changes have been most successful in tackling exclusion from the workplace due to sickness and disability, include those with systems for early identification of occupational health problems and where employer incentives and legislation have supported workers to exercise their right to remain in work[60].

Strengths and Limitations

This is the largest study to analyse fit note use and clinical information from GP records at point of first fit note, using individual-level clinical data. A major strength of using clinical records data

For peer review only

is that they contain more detail on condition type than information reported on the fit note which previous research has relied on. Clinical record data also provided usable information on reason for fit note for 98% of first fit notes, compared to the 50% of NHS Digital fit notes with diagnostic information. The study was limited because we do not have access to the information written on the fit note to compare it with the information recorded in clinical notes, and we do not know the length of time that each fit note was prescribed for. We also did not have access to sickness certification before the start of our study window, therefore our results do not represent lifetime first fit note, but first fit note in the study window. Each of the condition groups and subgroups encompasses a very wide variety of disorders. For example, the subgroup 'drug and/or alcohol misuse' is made up of harmful use and dependence diagnoses on multiple different substances (see Supplementary Table 2). The lack of granularity in the 'drug and/or alcohol' subgroup, and other groups/subgroups is a limitation.

The study was conducted in a single geographical area for which we had GP data. This had the benefit of providing information on virtually all individuals seeking help within that population, but it had the disadvantage of a possible loss of generalisability, and our findings should be tested in other areas. There is additional information that would have been extremely useful, such as nature of employment, educational level or benefit use, which were not available in this dataset. Without this information it was impossible to ascertain whether fit notes were issued for individuals in work versus those already out of work who were applying for health benefits. Furthermore, we did not know the occupational status of individuals within the population—for example whether they were unemployed, retired or in full-time education.

Conclusions

For the first time, we show drug and/or alcohol misuse at first fit note is associated with the highest number of fit notes, and that there is evidence that co-morbid mental health conditions at first fit note may have a multiplier effect on predicted number of fit notes for individuals presenting with infection. Demographic risk factors for high numbers of fit notes include Black Caribbean, Black Other or Mixed ethnicity, and higher levels of deprivation; these are likely to be explained by risk factors that we have not been able to measure, such as high risk occupations[10] and work place discrimination[48]. Linked occupational and health data sets are needed to better understand the occupational, clinical and wider structural risk factors for high number of fit notes, and to explore the trajectories of individuals after first fit note use.

For peer review only

Author Contributions: Sarah Dorrington designed the study, analysed the data and drafted the manuscript. Matthew Hotopf, Stephani Hatch and Ira Madan advised extensively throughout the process. Ewan Carr advised on the statistical analysis. Catherine Polling advised on the categorisation of read codes into groups and subgroups. Matthew Broadbent, Mark Ashworth and Sharon Stevelink provided expertise and guidance on extracting, analysing and interpreting the data. Emmert Roberts and all authors commented on the final manuscript.

Ethics approval CRIS was established in 2008 and approved by the Oxfordshire Research Ethics Committee in 2008 (reference 18/SC/0372). Approval for linkage with Lambeth DataNet was granted by Lambeth Clinical Commissioning Group and Information Governance Committee. This project was approved by the CRIS Oversight Committee in 2015. All patients have the choice to opt-out of their anonymised data being used.

Data availability statement Data may be obtained from a third party and are not publicly available. Access to the deidentified data in this study can be applied for through Lambeth Datanet and South London and Maudsley Biomedical Research Centre (BRC), De Crespigny Park, London SE5 8AF (cris.administrator@slam.nhs.uk). Access is granted only if approval is given by both the BRC and the Lambeth Datanet Steering Group.

Patient and public involvement: The findings of this study were presented to and discussed with the NIHR Biomedical Research Nucleus Data Linkage Service User and Carer Advisory Group.

References

1. Henderson M, Clark C, Stansfeld S, et al. A lifecourse approach to long-term sickness absence-a Cohort study. *PLoS ONE* 2012;7(5):e36645. doi: 10.1371/journal.pone.0036645
2. Health and safety at work: summary statistics for Great Britain 2019: Health and Safety Executive, 2019.
3. Coggon D, Palmer KT. Assessing fitness for work and writing a “fit note”. *BMJ (Clinical research ed)* 2010;341:c6305.
4. Thornhill S, Williams N. Completion of the statement of fitness for work: concise guidance. *Clin Med (Lond)* 2012;12(1):63-6. [published Online First: 2012/03/01]
5. Digital N. Fit notes issued by GP practices : December 2014 - March 2017 : experimental statistics. 2017
6. Gabbay MB. Electronic fit notes: sickness certification in the new decade. *Br J Gen Pract* 2010;60(573):235-6. doi: 10.3399/bjgp10X483841
7. NHSDigital. Fit notes issued by GP practices, England. December 2014 - March 2017 2017 [Available from: https://digital.nhs.uk/data-and-information/publications/statistical/fit-notes-issued-by-gp-practices/fit-notes-issued-by-gp-practices-england-december-2014-march-2017.

For peer review only

8. Shiels C, Gabbay MB, Hillage J. Change in diagnosis on fit notes within an episode of certified sickness absence. *Family Practice* 2016;33(5):510-16.
9. Buist-Bouwman MA, de Graaf R, Vollebergh WA, et al. Comorbidity of physical and mental disorders and the effect on work-loss days. *Acta Psychiatr Scand* 2005;111(6):436-43. doi: 10.1111/j.1600-0447.2005.00513.x [published Online First: 2005/05/10]
10. Starzmann K, Hjerpe P, Dalemo S, et al. Diagnoses have the greatest impact on variation in sick-leave certification rate among primary-care patients in Sweden: A multilevel analysis including patient, physician and primary health-care centre levels. *Scand J Public Health* 2015;43(7):704-12. doi: 10.1177/1403494815591898 [published Online First: 2015/07/01]
11. Coole C, Potgieter I, Nouri F, et al. Return-to-work outcomes and usefulness of actual fit notes received by employers. *Family Practice* 2015;32(5):551-56.
12. Hussey S, Hoddinott P, Wilson P, et al. Sickness certification system in the United Kingdom: qualitative study of views of general practitioners in Scotland. *BMJ (Clinical research ed)* 2004;328(7431):88. doi: 10.1136/bmj.37949.656389.EE
13. Digital N. Fit Notes Issued by GP Practices 2020 [Available from: https://digital.nhs.uk/data-and-information/publications/statistical/fit-notes-issued-by-gp-practices/guide#csv-metadata accessed April 2020.
14. Rachman R, Bunce D, Thorley K, et al. Patients' attitudes to sickness certification in general practice. *Occupational medicine (Oxford, England)* 2015;65(6):485-8. doi: 10.1093/occmed/kqv069 [published Online First: 2015/06/17]
15. Fletcher-Brown R. 'Mind Your Own Business' - how can we best support employers to improve the mental health of their employees? *Perspect Public Heal* 2015;135(1):18-20.
16. Dorrington S, Roberts E, Mykletun A, et al. Systematic review of fit note use for workers in the UK. *Occupational and environmental medicine* 2018 doi: 10.1136/oemed-2017-104730 [published Online First: 2018/05/08]
17. Gabbay M, Shiels C, Hillage J. Factors associated with the length of fit note-certified sickness episodes in the UK. *Occupational and environmental medicine* 2015;72(7):467-75. doi: 10.1136/oemed-2014-102307 [published Online First: 2015/02/26]
18. Dorrington S, Carr E, Stevelink SAM, et al. Demographic variation in fit note receipt and long-term conditions in south London. *Occupational & Environmental Medicine* 2020 doi: 10.1136/oemed-2019-106035 [published Online First: 2020/03/27]
19. Making more of multimorbidity: an emerging priority. The Lancet: Elsevier, 2018:1637.
20. Lambeth Council: State of the Borough. Lambeth Council, 2016.
21. Overview of London Boroughs. https://www.trustforlondon.org.uk/data/boroughs/overview-of-london-boroughs/. : Trust for London: Tackling poverty and inequality, 2019.
22. Mixed and balanced communities: Census Information Service, Greater London Authority, London, 2014.
23. Patient Register: quality assurance of administrative data used in population statistics: Office for National Statistics; 2016 [Available from: https://www.ons.gov.uk/peoplepopulationandcommunity/populationandmigration/populationestimates/methodologies/patientregisterqualityassuranceofadministrativedatausedinpopulationstatisticsdec2016.

24. LSOA Atlas https://data.london.gov.uk/dataset/lsoa-atlas: Greater London Authority 2014.
25. 2011 Census analysis: Ethnicity and religion of the non-UK born population in England and Wales: 2011. In: Statistics OoN, ed. https://www.ons.gov.uk/peoplepopulationandcommunity/culturalidentity/ethnicity/articles/2011censusanalysisethnicityandreligionofthenonukbornpopulationinenglandandwales/2015-06-18, 2011.
26. Hatch SL, Frissa S, Verdecchia M, et al. Identifying socio-demographic and socioeconomic determinants of health inequalities in a diverse London community: the South East London Community Health (SELCoH) study. *BMC Public Health* 2011;11:861. doi: 10.1186/1471-2458-11-861 [published Online First: 2011/11/15]
27. The English Index of Multiple Deprivation (IMD) 2015. In: Government DfCaL, ed. https://assets.publishing.service.gov.uk/government/uploads/system/uploads/attachment_data/file/464430/English_Index_of_Multiple_Deprivation_2015_-_Guidance.pdf, 2015.
28. Shiels C, Gabbay MB, Hillage J. Change in diagnosis on fit notes within an episode of certified sickness absence. *Fam Pract* 2016;33(5):510-6. doi: 10.1093/fampra/cmw050 [published Online First: 2016/06/28]
29. Shiels C, Gabbay M, Hillage J. Factors associated with prevalence and types of 'may be fit' advice on fit notes: a cross-sectional primary care analysis. *Br J Gen Pract* 2014;64(620):e137-43. doi: 10.3399/bjgp14X677509 [published Online First: 2014/02/26]
30. The King’s Fund. Impact of Quality and Outcomes Framework on health inequalities. 2011
31. Economou A, Grey M, McGregor J, et al. The health informatics cohort enhancement project (HICE): using routinely collected primary care data to identify people with a lifetime diagnosis of psychotic disorder. *BMC Research Notes* 2012;5:95. doi: 10.1186/1756-0500-5-95 [published Online First: 2012/02/16]
32. QOF Business Rules - version 35. Department of Health 2017.
33. From One to Many: Exploring people’s progression to multiple long-term conditions in an urban environment https://www.gsttcharity.org.uk/what-we-do/our-programmes/multiple-long-term-conditions/one-many-exploring-peoples-progression2018 [
34. Rogers W. Regression standard errors in clustered samples. *Stata Technical Bulletin* 1994;3(13)
35. Shiels CH, J; Pollard E; Gabbay, M. Evaluation of the statement of fitness for work (fit note) : quantitative survey of fit notes. *Research report* 2013(no 841):95 pages.
36. Gabbay M, Shiels C, Hillage J. Sickness certification for common mental disorders and GP return-to-work advice. *Primary Health Care Research Development* 2016:1-11. doi: 10.1017/s1463423616000074 [published Online First: 2016/03/11]
37. Scott KM, Bruffaerts R, Tsang A, et al. Depression-anxiety relationships with chronic physical conditions: results from the World Mental Health Surveys. *Journal of Affective Disorders* 2007;103(1-3):113-20. doi: 10.1016/j.jad.2007.01.015 [published Online First: 2007/02/13]

38. Teesson M, Mitchell PB, Deady M, et al. Affective and anxiety disorders and their relationship with chronic physical conditions in Australia: findings of the 2007 National Survey of Mental Health and Wellbeing. *Australian and New Zealand Journal of Psychiatry* 2011;45(11):939-46. doi: 10.3109/00048674.2011.614590 [published Online First: 2011/10/05]
39. Teesson M, Slade T, Mills K. Comorbidity in Australia: findings of the 2007 National Survey of Mental Health and Wellbeing. *Aust N Z J Psychiatry* 2009;43(7):606-14. doi: 10.1080/00048670902970908 [published Online First: 2009/06/17]
40. Li C, Ford ES, Zhao G, et al. Prevalence and correlates of undiagnosed depression among U.S. adults with diabetes: the Behavioral Risk Factor Surveillance System, 2006. *Diabetes Res Clin Pract* 2009;83(2):268-79. doi: 10.1016/j.diabres.2008.11.006 [published Online First: 2008/12/30]
41. Das-Munshi J, Stewart R, Ismail K, et al. Diabetes, common mental disorders, and disability: findings from the UK National Psychiatric Morbidity Survey. *Psychosomatic Medicine* 2007;69(6):543-50. doi: 10.1097/PSY.0b013e3180cc3062 [published Online First: 2007/07/20]
42. Tsang A, Von Korff M, Lee S, et al. Common chronic pain conditions in developed and developing countries: gender and age differences and comorbidity with depression-anxiety disorders. *J Pain* 2008;9(10):883-91. doi: 10.1016/j.jpain.2008.05.005 [published Online First: 2008/07/08]
43. Moussavi S, Chatterji S, Verdes E, et al. Depression, chronic diseases, and decrements in health: results from the World Health Surveys. *Lancet* 2007;370(9590):851-8. doi: 10.1016/S0140-6736(07)61415-9 [published Online First: 2007/09/11]
44. Evans DL, Charney DS, Lewis L, et al. Mood disorders in the medically ill: scientific review and recommendations. *Biol Psychiatry* 2005;58(3):175-89. doi: 10.1016/j.biopsych.2005.05.001 [published Online First: 2005/08/09]
45. Whooley MA, Caska CM, Hendrickson BE, et al. Depression and inflammation in patients with coronary heart disease: findings from the Heart and Soul Study. *Biol Psychiatry* 2007;62(4):314-20. doi: 10.1016/j.biopsych.2006.10.016 [published Online First: 2007/04/17]
46. Chapman DP, Perry GS, Strine TW. The vital link between chronic disease and depressive disorders. *Prev Chronic Dis* 2005;2(1):A14. [published Online First: 2005/01/27]
47. Woodhead C, Ashworth M, Schofield P, et al. Patterns of physical co-/multi-morbidity among patients with serious mental illness: a London borough-based cross-sectional study. *BMC Family Practice* 2014;15:117. doi: 10.1186/1471-2296-15-117 [published Online First: 2014/06/13]
48. Hatch SL, Gazard B, Williams DR, et al. Discrimination and common mental disorder among migrant and ethnic groups: findings from a South East London Community sample. *Soc Psychiatry Psychiatr Epidemiol* 2016 doi: 10.1007/s00127-016-1191-x
49. Hatch SL, Woodhead C, Frissa S, et al. Importance of thinking locally for mental health: data from cross-sectional surveys representing South East London and England. *PLoS One* 2012;7(12):e48012. doi: 10.1371/journal.pone.0048012 [published Online First: 2012/12/20]
50. Labour Force Survey: Sickness absence in the UK labour market 2018 GovUK, 2018.

51. Roche A, Pidd K, Kostadinov V. Alcohol- and drug-related absenteeism: a costly problem. *Aust N Z J Public Health* 2016;40(3):236-8. doi: 10.1111/1753-6405.12414 [published Online First: 2015/08/12]
52. Bockerman P, Hyytinen A, Maczulskij T. Devil in disguise: Does drinking lead to a disability pension? *Prev Med* 2016;86:130-5. doi: 10.1016/j.ypmed.2016.03.008 [published Online First: 2016/03/13]
53. Jones E, Fear NT. Alcohol use and misuse within the military: a review. *Int Rev Psychiatry* 2011;23(2):166-72. doi: 10.3109/09540261.2010.550868 [published Online First: 2011/04/28]
54. Fowlie DG. The misuse of alcohol and other drugs by doctors: a UK report and one region's response. *Alcohol Alcohol* 1999;34(5):666-71. doi: 10.1093/alcalc/34.5.666 [published Online First: 1999/10/21]
55. Flannery J, Ajayi SO, Oyegoke AS. Alcohol and substance misuse in the construction industry. *Int J Occup Saf Ergon* 2019:1-16. doi: 10.1080/10803548.2019.1601376 [published Online First: 2019/04/03]
56. Brooke DE, Griffiths; Taylor, Colin. Addiction as an occupational hazard. *British journal of addiction* 1991
57. Drummond C. Cuts to addiction services are a false economy. *BMJ : British Medical Journal* 2017
58. Roberts E, Hotopf M, Drummond C. The relationship between alcohol-related hospital admission and specialist alcohol treatment provision across local authorities in England since passage of the Health and Social Care Act 2012. *Br J Psychiatry* 2020:1-3. doi: 10.1192/bjp.2020.120 [published Online First: 2020/07/02]
59. Ballard J. TfL's Drug and Alcohol Assessment and Treatment Service. *Occupational Health at Work* 2019;15/5:36-38.
60. Hemmings P. Sickness and disability systems: comparing outomes and policies in Norway with those in Sweden, Netherlands and Switzerland. 2020

For peer review only

Health condition at first fit note and number of fit notes: evidence from a large primary care sample in south London

Table 1 Number of fit notes received and predicted number of fit notes by sociodemographic variables

Demographic variables	Number of fit notes received: 1st January 2014 to 20th April 2016 n (%)				Predicted NFN unadjusted (95%CI)	Predicted number of fit notes (95% CI)	
	1	2	3	4+		Model 1: Adjusted for age, gender, ethnicity and deprivation	Model 2: +long-term conditions
Whole pop	40,698				2.0 [1.8,2.1]	2.2 [2.1,2.3]	2.4 [2.3,2.5]
Gender							
Male	6,097 (35.2)	3,014 (17.4)	1,878 (10.9)	6,312 (36.5)	2.0 [1.8,2.2]	2.2 [2.1,2.3]	2.4 [2.3,2.6]
Female	8,304 (35.5)	4,231 (18.1)	2,557 (10.9)	8,305 (35.5)	1.9 [1.8,2.1]	2.2 [2.1,2.3]	2.3 [2.2,2.4]
Age group							
16-20	428 (46.9)	178 (19.5)	80 (8.8)	227 (24.9)	1.4 [1.2,1.6]	1.4 [1.2,1.6]	1.7 [1.5,1.9]
21-25	2,054 (49.3)	807 (19.4)	379 (9.1)	926 (22.2)	1.3 [1.2,1.5]	1.4 [1.3,1.5]	1.7 [1.5,2.8]
36-30	2,825 (46.0)	1,138 (18.5)	650 (10.6)	1,532 (24.9)	1.5 [1.4,1.6]	1.6 [1.4,1.7]	1.8 [2.7,2.9]
31-35	2,311 (38.7)	1,136 (19.0)	691 (11.6)	1,839 (30.8)	1.8 [1.7,2.0]	1.9 [2.8,2.0]	2.2 [2.1,2.3]
36-40	1,771 (34.3)	996 (19.3)	591 (11.5)	1,803 (34.9)	2.1 [2.0,2.3]	2.2 [2.0,2.3]	2.4 [2.3,2.5]
41-45	1,541 (31.2)	881 (17.8)	532 (10.8)	1,985 (40.2)	2.4 [2.3,2.6]	2.5 [2.3,2.6]	2.6 [2.4,2.7]
46-50	1,528 (28.1)	880 (16.2)	594 (10.9)	2,437 (44.8)	2.7 [2.5,2.9]	2.7 [2.6,2.9]	2.7 [2.5,2.8]
51-55	1,226 (26.2)	709 (15.1)	536 (11.4)	2,218 (47.3)	2.9 [2.7,3.1]	2.9 [1.8,3.1]	2.8 [2.6,2.9]
56-60	717 (21.9)	520 (15.9)	382 (11.7)	1,650 (50.5)	3.1 [2.9,3.3]	3.2 [3.0,3.4]	2.9 [2.7,3.0]
Ethnicity							
White	8,592 (39.4)	3,979 (18.2)	2,264 (10.4)	6,980 (32.0)	1.8 [1.6,2.0]	2.0 [1.9,2.1]	2.2 [2.1,2.3]
Black African	1,889 (32.4)	1,090 (18.7)	650 (11.1)	2,211 (37.9)	2.1 [2.0,2.3]	2.2 [2.1,2.4]	2.4 [2.2,2.5]
Asian	845 (35.5)	430 (18.1)	292 (12.3)	815 (34.2)	1.9 [1.7,2.2]	2.2 [1.9,2.4]	2.3 [2.1,2.5]
Black Caribbean	1,279 (25.4)	809 (16.1)	567 (11.3)	2,380 (47.3)	2.7 [2.5,2.9]	2.8 [2.7,2.9]	2.9 [2.8,3.0]
Mixed	774 (31.5)	404 (16.5)	300 (12.2)	978 (39.8)	2.2 [2.1,2.4]	2.6 [2.4,2.8]	2.7 [2.6,2.9]
Other	503 (36.2)	268 (19.3)	167 (12.0)	450 (32.4)	1.9 [1.7,2.1]	2.0 [1.8,2.2]	2.3 [2.1,2.5]
Black Other	519 (29.1)	265 (14.9)	195 (10.9)	803 (45.1)	2.6 [2.4,2.8]	2.8 [2.6,3.0]	2.9 [2.7,3.2]
Deprivation							
1	2,325 (39.8)	1,142 (19.5)	626 (10.7)	1,750 (30.0)	1.7 [1.5,1.8]	1.9 [1.8,2.0]	2.0 [2.0,2.2]
2	2,575 (38.1)	1,265 (18.1)	705 (10.4)	2,254 (33.3)	1.8 [1.7,2.0]	2.1 [2.9,2.2]	2.1 [2.1,2.4]
3	2,725 (37.6)	1,411 (17.1)	823 (11.4)	2,836 (33.9)	1.9 [1.7,2.1]	2.1 [2.0,2.3]	2.1 [2.1,2.4]
4	3,067 (32.7)	1,606 (17.8)	1,033 (11.0)	3,521 (38.5)	2.2 [2.0,2.3]	2.3 [2.2,2.5]	2.4 [2.4,2.6]
5	3,353 (32.1)	1,817 (17.1)	1,134 (10.9)	4,195 (39.9)	2.2 [2.1,2.4]	2.4 [2.3,2.6]	2.6 [2.4,2.7]

*mutually adjusted for age, gender, ethnicity and deprivation **mutually adjusted as above, also adjusted for number of long-term conditions (QOF)

For peer review only

Table 2 Predicted number of fit notes by diagnostic group

Category	Groups	Selected sub-groups	N people (%)	Predicted number of fit notes (NFN) [95%CI]	Predicted NFN adj* [95%CI]	Predicted NFN adj** [95%CI]
Mental Health	Total		40,698	2.0 [1.8,2.1]	2.2 [2.1,2.3]	2.4 [2.3,2.5]
	Mental illness***		5,841 (14.4)	2.8 [2.6,3.0]	3.3 [3.1,3.5]	3.3 [3.1,3.4]
		Drug and alcohol	584 (1.4)	3.9 [3.6,4.3]	4.2 [3.8,4.6]	4.5 [4.1,4.8]
		Common Mental Disorders	4,393(10.8)	2.7 [2.5,2.9]	3.2 [3.1,3.4]	3.2 [3.1,3.3]
		Severe Mental Illness	374 (0.9)	2.7 [2.5,3.0]	3.0 [2.8,3.3]	2.6 [2.4,2.8]
	Stress		1,037 (2.6)	2.1 [1.8,2.3]	2.5 [2.2,2.7]	2.7 [2.4,2.9]
	Stress Ext		845 (2.1)	2.2 [1.9,2.4]	2.3 [2.1,2.5]	2.5 [2.3,2.7]
Non Mental Health	MSK		8,366 (20.6)	2.2 [2.1,2.4]	2.3 [2.2,2.5]	2.6 [2.4,2.7]
	Fatigue		541 (1.3)	1.9 [1.6,2.2]	2.2 [2.0,2.5]	2.4 [2.1,2.7]
	Injury		2,183 (5.4)	1.8 [1.6,1.9]	2.1 [1.9,2.2]	2.3 [2.2,2.5]
	Physical health		12,710 (31.2)	2.1 [1.9,2.3]	2.2 [2.1,2.3]	2.3 [2.2,2.4]
	Other		448 (1.1)	2.0 [1.8,2.3]	2.2 [2.0,2.4]	2.3 [2.1,2.5]
	Obstetrics		1,372 (3.4)	1.4 [1.3,1.6]	2.0 [1.8,2.2]	2.2 [2.0,2.4]
	Infection		6,726 (16.5)	1.5 [1.3,1.6]	1.8 [1.7,1.9]	1.9 [1.8,2.0]
	Surgery		629 (1.6)	1.5 [1.3,1.8]	1.6 [1.4,1.9]	1.8 [1.5,2.1]

For peer review only

*adjusted for age, gender, ethnicity and deprivation
**adjusted for age, gender, ethnicity, deprivation and number of long-term conditions
***made up of drug & alcohol, common mental disorders, severe mental illness, mental health treatment and mental health other

For peer review only

Figure 1

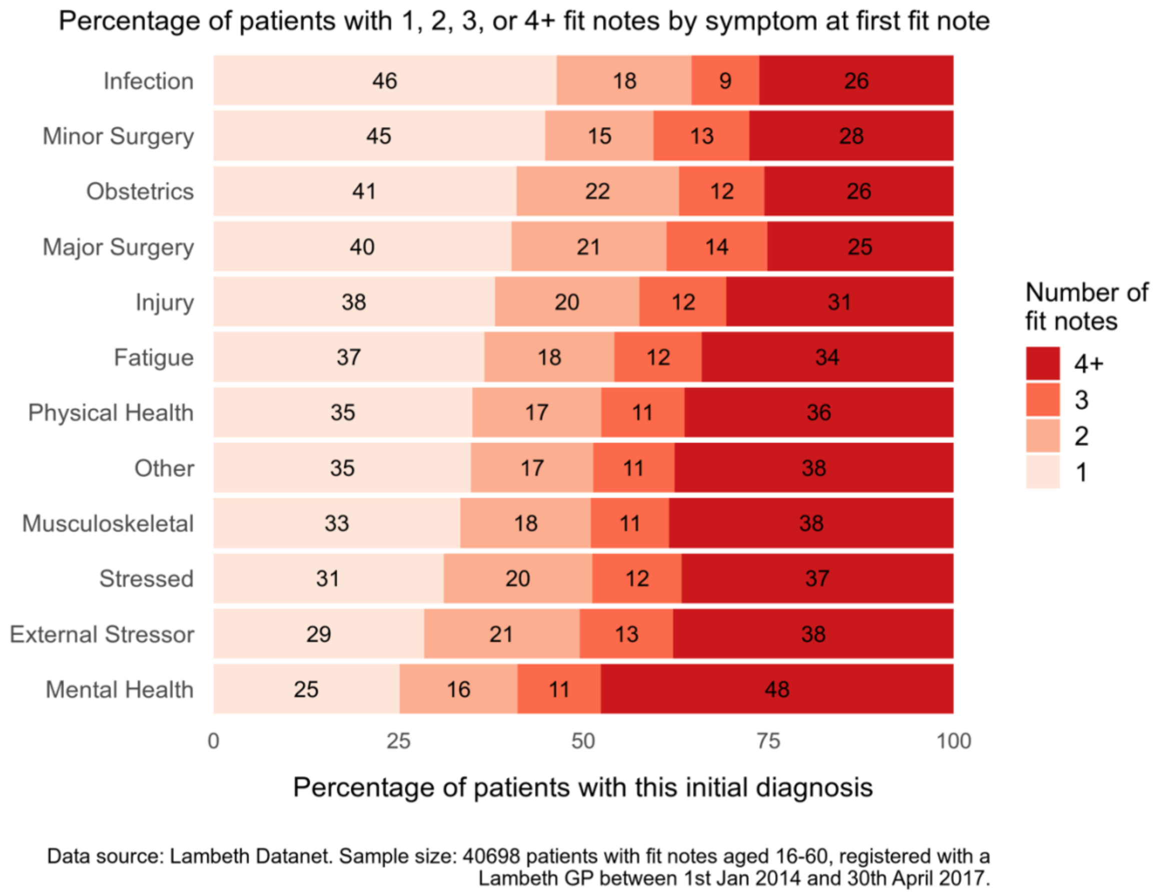
Percentage of patients with 1, 2, 3, or 4+ fit notes by symptom at first fit note
Infection 46 18 9 26
Minor Surgery 45 15 13 28
Obstetrics 41 22 12 26
Major Surgery 40 21 14 25
Injury 38 20 12 31
Fatigue 37 18 12 34
Physical Health 35 17 11 36
Other 35 17 11 38
Musculoskeletal 33 18 11 38
Stressed 31 20 12 37
External Stressor 29 21 13 38
Mental Health 25 16 11 48
0 25 50 75 100
Percentage of patients with this initial diagnosis
Number of fit notes
4+
3
2
1
Data source: Lambeth Datanet. Sample size: 40698 patients with fit notes aged 16-60, registered with a Lambeth GP between 1st Jan 2014 and 30th April 2017.

For peer review only

SUPPLEMENTARY MATERIAL

Figure 1 Classification of conditions into sub-groups, groups and categories

**Categories**	**Groups**	**Sub-groups**
Non-Mental Health	Injury*	Injury*
	Infection*	Infection*
	Surgery*	Minor Surgery*<br>Major Surgery*
	Fatigue/Insomnia	Insomnia<br>Fatigue
	Obstetrics	Obstetrics
	Musculoskeletal	Musculoskeletal
	Physical Health	Cancer<br>Neurology<br>Haematology<br>Obesity<br>Test<br>Cardiovascular<br>Gastroenterology<br>Gynaecology<br>Dermatology<br>Respiratory<br>Physical Health Treatment<br>Genitourinary Medicine/Urology<br>Allergy<br>Risk factor<br>Ear, Nose, Throat and Dental<br>Renal<br>Diabetes<br>Systemic Illness<br>Physical symptom<br>Wound
	Other	Other Physical Health
Mental Health	Mental Illness	Common Mental Disorders<br>Severe Mental Illness<br>Drug and Alcohol<br>Mental Health Treatment<br>Other Mental Health Problem**
	External Stressor	Bereavement<br>External stressor
	Stressed	Stressed

*Injury, infection and surgery were at the top of the two-tier hierarchy of groupings, as an alternative to assigning injuries, infections and surgery to their system groups.
** Other mental health problem includes self-harm, developmental disorders, personality disorder

Table 1 Predicted number of fit notes by condition type

Condition sub-group	Number (%)	Predicted number of fit notes (NFN)	Predicted NFN adj*	Predicted NFN adj**
Drug and alcohol	584 (1.4)	3.9 (3.6-4.3)	4.2 (3.8-4.6)	4.5 (4.1-4.8)
Other Mental Health Problem	294 (0.7)	2.7 (2.3-3.0)	3.7 (3.2-4.2)	3.6 (3.1-4.0)
Common Mental Disorders	4,393(10.8)	2.7 (2.5-2.9)	3.2 (3.1-3.4)	3.2 (3.1-3.3)
Severe Mental Illness	374 (0.9)	2.7 (2.5-3.0)	3.0 (2.8-3.3)	2.6 (2.4-2.8)
Neurology	640 (1.6)	2.5 (2.2-2.8)	2.8 (2.5-3.1)	2.8 (2.5-3.1)
Haematology	134 (0.3)	2.5 (2.1-2.9)	2.8 (2.3-3.3)	3.2 (2.6-3.8)
Obesity	74 (0.2)	2.7 (2.0-3.3)	2.7 (2.0-3.4)	2.4 (1.8-2.9)
Insomnia	174 (0.4)	2.2 (1.8-2.7)	2.6 (2.0-3.2)	2.7 (2.1-3.3)
Mental Health Treatment	192 (0.5)	2.3 (1.7-2.8)	2.6 (2.0-3.1)	2.4 (1.9-2.9)
Cancer	239 (0.6)	2.5 (2.3-2.8)	2.5 (2.2-2.7)	2.5 (2.2-2.7)
Stressed	1,039 (2.6)	2.1 (1.9-2.3)	2.5 (2.2-2.7)	2.7 (2.5-2.9)
Diabetes	58 (0.1)	2.8 (2.0-3.6)	2.5 (1.8-3.2)	2.1 (1.5-2.7)
Systemic Illness	1,028 (2.5)	2.6 (2.3-2.8)	2.4 (2.2-2.6)	2.1 (1.9-2.3)
Physical symptom	1,439 (3.5)	2.2 (2.0-2.4)	2.4 (2.2-2.5)	2.5 (2.3-2.6)
Other	326 (0.8)	2.2 (1.9-2.5)	2.4 (2.1-2.6)	2.5 (2.2-2.7)
External stressor	349 (0.9)	2.1 (1.8-2.5)	2.4 (2.0-2.7)	2.6 (2.2-2.9)
Musculoskeletal	8,288 (20.4)	2.3 (2.1 2.4)	2.3 (2.2-2.5)	2.6 (2.4-2.7)
Bereavement	495 (1.2)	2.2 (1.9-2.5)	2.3 (2.1-2.6)	2.5 (2.3-2.8)
Test	26 (0.1)	2.7 (1.7-3.7)	2.3 (1.6-3.1)	2.3 (1.6-3.0)
Cardiovascular	1,234 (3.0)	2.5 (2.2-2.7)	2.2 (2.1-2.4)	2.2 (2.0-2.3)
Gastroenterology	2,241 (5.5)	2.0 (1.8-2.1)	2.2 (2.1-2.4)	2.4 (2.3-2.6)
Gynaecology	768 (1.9)	1.9 (1.7-2.0)	2.2 (2.0-2.3)	2.4 (2.3-2.6)
Dermatology	1,129 (2.8)	1.9 (1.6-2.1)	2.2 (1.9-2.4)	2.3 (2.1-2.6)
Respiratory	1,663 (4.1)	2.0 (1.8-2.2)	2.1 (2.0-2.3)	2.0 (1.9-2.2)
Injury	2,183 (5.4)	1.8 (1.6-2.0)	2.1 (1.9-2.3)	2.4 (2.2-2.5)
Fatigue	367 (0.9)	1.8 (1.5-2.1)	2.1 (1.8-2.4)	2.3 (2.0-2.6)
Physical Health Treatment	13 (0.0)	1.9 (1.0-2.9)	2.1 (1.0-3.2)	2.1 (0.8-3.3)
Genitourinary Medicine/Urology	481 (1.2)	2.0 (1.7-2.3)	2.0 (1.8-2.3)	2.1 (1.9-2.4)
Obstetrics	1,371 (3.4)	1.4 (1.3-1.6)	2.0 (1.8-2.2)	2.2 (2.0-2.5)
Ear, Nose, Throat and Dental	1,016 (2.5)	1.8 (1.6-2.1)	2.0 (1.7-2.2)	2.1 (1.9-2.4)
Wound	562 (1.4)	1.8 (1.6-2.0)	1.9 (1.8-2.1)	2.1 (1.9-2.3)
Infection	6,727 (16.5)	1.5 (1.4-1.7)	1.8 (1.7-1.9)	1.9 (1.8-2.0)
Risk factor	33 (0.1)	1.8 (1.1-2.5)	1.8 (1.2-2.3)	1.8 (1.2-2.4)
Minor Surgery	232 (0.6)	1.6 (1.3-1.9)	1.7 (1.4-2.0)	1.9 (1.5-2.3)
Allergy	88 (0.2)	1.4 (1.0-1.9)	1.7 (1.2-2.2)	1.9 (1.3-2.4)
Major Surgery	397 (1.0)	1.6 (1.3-1.8)	1.6 (1.4-1.9)	1.8 (1.5-2.0)
Renal	47 (0.1)	1.6 (0.8-2.3)	1.6 (0.9-2.4)	1.9 (1.0-2.7)

*adjusted for age, gender, ethnicity and deprivation

**adjusted for age, gender, ethnicity, deprivation and number of long term conditions

For peer review only

Figure 2

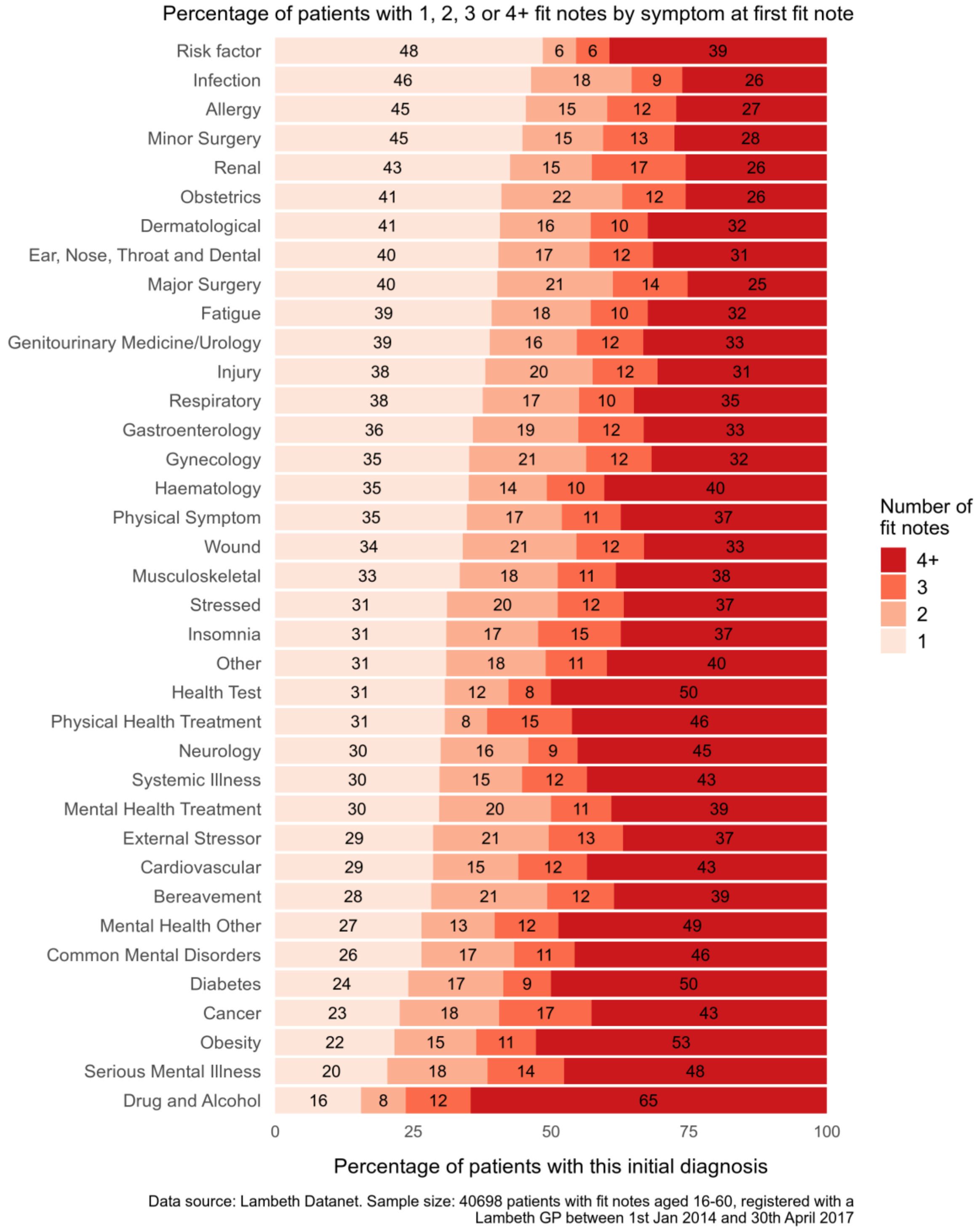
Percentage of patients with 1, 2, 3 or 4+ fit notes by symptom at first fit note
Risk factor
48
6
6
39
Infection
46
18
9
26
Allergy
45
15
12
27
Minor Surgery
45
15
13
28
Renal
43
15
17
26
Obstetrics
41
22
12
26
Dermatological
41
16
10
32
Ear, Nose, Throat and Dental
40
17
12
31
Major Surgery
40
21
14
25
Fatigue
39
18
10
32
Genitourinary Medicine/Urology
39
16
12
33
Injury
38
20
12
31
Respiratory
38
17
10
35
Gastroenterology
36
19
12
33
Gynecology
35
21
12
32
Haematology
35
14
10
40
Physical Symptom
35
17
11
37
Wound
34
21
12
33
Musculoskeletal
33
18
11
38
Stressed
31
20
12
37
Insomnia
31
17
15
37
Other
31
18
11
40
Health Test
31
12
8
50
Physical Health Treatment
31
8
15
46
Neurology
30
16
9
45
Systemic Illness
30
15
12
43
Mental Health Treatment
30
20
11
39
External Stressor
29
21
13
37
Cardiovascular
29
15
12
43
Bereavement
28
21
12
39
Mental Health Other
27
13
12
49
Common Mental Disorders
26
17
11
46
Diabetes
24
17
9
50
Cancer
23
18
17
43
Obesity
22
15
11
53
Serious Mental Illness
20
18
14
48
Drug and Alcohol
16
8
12
65
0
25
50
75
100
Percentage of patients with this initial diagnosis
Number of fit notes
4+
3
2
1
Data source: Lambeth Datanet. Sample size: 40698 patients with fit notes aged 16-60, registered with a Lambeth GP between 1st Jan 2014 and 30th April 2017

Table 2 Example of subgroup codes:

Drug and Alcohol Codes:
[RFC] Drug/ substance abuse
[RFC] Substance abuse
[X]Alcohol addiction
[X]Drug addiction - cannabis
[X]Heroin addiction
[X]Men & behav disorder multiple drug use/psychoactive subst
[X]Mental and behav dis due to use alcohol: dependence syndr
[X]Mental and behav dis due to use cannabinoids: harmful use
[X]Mental and behav dis due to use of alcohol: harmful use
[X]Mental and behav dis due to use of cocaine: harmful use
[X]Mental and behav dis due to use of opioids: harmful use
[X]Mental and behav dis due to use opioids: dependence syndr
[X]Mental behav disorders due use crack cocaine: harmful use
Alcohol dependence syndrome
Alcohol dependence syndrome NOS
Alcohol misuse - enhanced service completed
Alcohol problem drinking
Alcohol withdrawal syndrome
Alcoholism
Analgesic overuse headache
Benzodiazepine dependence
Cannabis dependence, continuous
Cannabis dependence, unspecified
Cannabis type drug dependence
Chronic alcoholism
Chronic alcoholism in remission
Cocaine dependence, episodic
Cocaine dependence, unspecified
Cocaine drug dependence NOS
Cocaine type drug dependence
Diazepam dependence
Drug addiction
Drug addiction therapy
Drug addictn therap-methadone
Drug dependence
Drug dependence NOS
Drug dependence therapy
Drug seeking behaviour
Drug withdrawal syndrome
Drunkenness NOS
Hangover (alcohol)
Harmful alcohol use
Hazardous alcohol use
Heavy drinker - 7-9u/day
Heroin dependence
Intoxication - alcohol
Methadone dependence
Misuse of drugs NOS
Morphine dependence
Nondependent alcohol abuse
Nondependent cannabis abuse
Opioid drug dependence NOS
Opioid type drug dependence
Refer to drugs service
Very heavy drinker - >9u/day

For peer review only

For peer review only

Reporting checklist for cohort study.

Based on the STROBE cohort guidelines.

Instructions to authors

Complete this checklist by entering the page numbers from your manuscript where readers will find each of the items listed below.

Your article may not currently address all the items on the checklist. Please modify your text to include the missing information. If you are certain that an item does not apply, please write "n/a" and provide a short explanation.

Upload your completed checklist as an extra file when you submit to a journal.

In your methods section, say that you used the STROBE cohortreporting guidelines, and cite them as:

von Elm E, Altman DG, Egger M, Pocock SJ, Gotzsche PC, Vandenbroucke JP. The Strengthening the Reporting of Observational Studies in Epidemiology (STROBE) Statement: guidelines for reporting observational studies.

For peer review only

		Reporting Item	Page Number
**Title and abstract**			
Title	#1a	Indicate the study’s design with a commonly used term in the title or the abstract	1
Abstract	#1b	Provide in the abstract an informative and balanced summary of what was done and what was found	3
**Introduction**			
Background / rationale	#2	Explain the scientific background and rationale for the investigation being reported	4
Objectives	#3	State specific objectives, including any prespecified hypotheses	4
**Methods**			
Study design	#4	Present key elements of study design early in the paper	5
Setting	#5	Describe the setting, locations, and relevant dates, including periods of	5

		recruitment, exposure, follow-up, and data collection	
Eligibility criteria	#6a	Give the eligibility criteria, and the sources and methods of selection of participants. Describe methods of follow-up.	6
Eligibility criteria	#6b	For matched studies, give matching criteria and number of exposed and unexposed	n/a
Variables	#7	Clearly define all outcomes, exposures, predictors, potential confounders, and effect modifiers. Give diagnostic criteria, if applicable	6 and 7
Data sources / measurement	#8	For each variable of interest give sources of data and details of methods of assessment (measurement). Describe comparability of assessment methods if there is more than one group. Give information separately for for exposed and unexposed groups if applicable.	6 and 7
Bias	#9	Describe any efforts to address potential sources of bias	11
Study size	#10	Explain how the study size was arrived at	7
Quantitative variables	#11	Explain how quantitative variables were handled in the analyses. If applicable, describe which groupings were chosen, and why	6
Statistical methods	#12a	Describe all statistical methods, including those used to control for confounding	7
Statistical methods	#12b	Describe any methods used to examine subgroups and interactions	7
Statistical methods	#12c	Explain how missing data were addressed	7
Statistical methods	#12d	If applicable, explain how loss to follow-up was addressed	n/a
Statistical methods	#12e	Describe any sensitivity analyses	n/a
**Results**			
Participants	#13a	Report numbers of individuals at each stage of study—eg numbers potentially eligible, examined for eligibility, confirmed eligible, included in the study, completing follow-up, and analysed. Give information separately for for exposed and unexposed groups if applicable.	7

For peer review only

Participants	#13b	Give reasons for non-participation at each stage	7
Participants	#13c	Consider use of a flow diagram	n/a
Descriptive data	#14a	Give characteristics of study participants (eg demographic, clinical, social) and information on exposures and potential confounders. Give information separately for exposed and unexposed groups if applicable.	7
Descriptive data	#14b	Indicate number of participants with missing data for each variable of interest	7
Descriptive data	#14c	Summarise follow-up time (eg, average and total amount)	na
Outcome data	#15	Report numbers of outcome events or summary measures over time. Give information separately for exposed and unexposed groups if applicable.	7 and 8
Main results	#16a	Give unadjusted estimates and, if applicable, confounder-adjusted estimates and their precision (eg, 95% confidence interval). Make clear which confounders were adjusted for and why they were included	7
Main results	#16b	Report category boundaries when continuous variables were categorized	7
Main results	#16c	If relevant, consider translating estimates of relative risk into absolute risk for a meaningful time period	na
Other analyses	#17	Report other analyses done—e.g., analyses of subgroups and interactions, and sensitivity analyses	9
**Discussion**			
Key results	#18	Summarise key results with reference to study objectives	9
Limitations	#19	Discuss limitations of the study, taking into account sources of potential bias or imprecision. Discuss both direction and magnitude of any potential bias.	11
Interpretation	#20	Give a cautious overall interpretation considering objectives, limitations, multiplicity of analyses, results from similar studies, and other relevant evidence.	11
Generalisability	#21	Discuss the generalisability (external validity) of the study results	11
**Other Information**			

For peer review only

Funding	#22	Give the source of funding and the role of the funders for the present study and, if applicable, for the original study on which the present article is based	1

The STROBE checklist is distributed under the terms of the Creative Commons Attribution License CC-BY. This checklist was completed on 16. August 2020 using https://www.goodreports.org/, a tool made by the EQUATOR Network in collaboration with Penelope.ai

For peer review only

BMJ Open

Health condition at first fit note and number of fit notes: evidence from a large primary care sample in south London

Journal:	*BMJ Open*
Manuscript ID	bmjopen-2020-043889.R1
Article Type:	Original research
Date Submitted by the Author:	05-Jan-2021
Complete List of Authors:	Dorrington, Sarah; King's College London Institute of Psychiatry Psychology and Neuroscience, Department of Psychological Medicine; South London and Maudsley NHS Foundation Trust, NIHR Maudsley Biomedical Research Centre<br>Carr, Ewan ; King's College London Institute of Psychiatry Psychology and Neuroscience, Department of Psychological Medicine<br>Polling, C; King's College London Institute of Psychiatry Psychology and Neuroscience, Department of Psychological Medicine; South London and Maudsley Mental Health NHS Trust, NIHR Maudsley Biomedical Research Centre<br>Stevelink, Sharon; King's College London Institute of Psychiatry Psychology and Neuroscience, Department of Psychological Medicine<br>Ashworth, Mark; King's College London, School of Population Health & Environmental Sciences<br>Roberts , Emmert; King's College London Institute of Psychiatry Psychology and Neuroscience, National Addiction Centre; South London and Maudsley Mental Health NHS Trust, NIHR Maudsley Biomedical Research Centre<br>Broadbent, Matthew; South London and Maudsley Mental Health NHS Trust, NIHR Maudsley Biomedical Research Centre<br>HATCH, STEPHANI; King's College London Institute of Psychiatry Psychology and Neuroscience, Department of Psychological Medicine<br>MADAN, IRA; Guy's and St Thomas' Hospitals NHS Trust, Department of Occupational Health<br>Hotopf, Matthew; King's College London Institute of Psychiatry Psychology and Neuroscience, Department of Psychological Medicine; South London and Maudsley Mental Health NHS Trust, NIHR Maudsley Biomedical Research Centre
**Primary Subject Heading**:	General practice / Family practice
Secondary Subject Heading:	Health policy, Health services research, Epidemiology, Occupational and environmental medicine, Public health
Keywords:	PRIMARY CARE, PUBLIC HEALTH, OCCUPATIONAL & INDUSTRIAL MEDICINE, Health policy < HEALTH SERVICES ADMINISTRATION & MANAGEMENT

SCHOLARONE™
Manuscripts

I, the Submitting Author has the right to grant and does grant on behalf of all authors of the Work (as defined in the below author licence), an exclusive licence and/or a non-exclusive licence for contributions from authors who are: i) UK Crown employees; ii) where BMJ has agreed a CC-BY licence shall apply, and/or iii) in accordance with the terms applicable for US Federal Government officers or employees acting as part of their official duties; on a worldwide, perpetual, irrevocable, royalty-free basis to BMJ Publishing Group Ltd ("BMJ") its licensees and where the relevant Journal is co-owned by BMJ to the co-owners of the Journal, to publish the Work in this journal and any other BMJ products and to exploit all rights, as set out in our licence.

The Submitting Author accepts and understands that any supply made under these terms is made by BMJ to the Submitting Author unless you are acting as an employee on behalf of your employer or a postgraduate student of an affiliated institution which is paying any applicable article publishing charge ("APC") for Open Access articles. Where the Submitting Author wishes to make the Work available on an Open Access basis (and intends to pay the relevant APC), the terms of reuse of such Open Access shall be governed by a Creative Commons licence – details of these licences and which Creative Commons licence will apply to this Work are set out in our licence referred to above.

Other than as permitted in any relevant BMJ Author's Self Archiving Policies, I confirm this Work has not been accepted for publication elsewhere, is not being considered for publication elsewhere and does not duplicate material already published. I confirm all authors consent to publication of this Work and authorise the granting of this licence.

For peer review only

Health condition at first fit note and number of fit notes: evidence from a large primary care sample in south London

Sarah Dorrington (1,2)*
Ewan Carr (1)
Catherine Polling (1,2)
Sharon A.M. Stevelink (1)
Mark Ashworth (3)
Emmert Roberts (1,2)
Matthew Broadbent (2)
Stephani L Hatch (1)
Ira Madan (4)
Matthew Hotopf (1,2)

(1) Department of Psychological Medicine, Institute of Psychiatry, Psychology & Neuroscience King's College London, 16 De Crespigny Park, London SE5 8AF
(2) NIHR Maudsley Biomedical Research Centre, South London and Maudsley NHS Foundation Trust, London, UK
(3) Department of Population Health Sciences, School of Population Health and Environmental Sciences, King's College London, Guy's Campus, Addison House, London SE1 1UL
(4) Department of Occupational Health, Guy's and St Thomas' NHS Foundation Trust, London, UK

Word count: 3508

*Corresponding author.
3rd Floor, Institute of Psychiatry, Psychology and Neuroscience, De Crespigny Park, London SE5 8AF; Sarah.dorrington@kcl.ac.uk; 02078485817.

For peer review only

We used the STROBE cohort checklist when writing our report.

Funding statement: This paper represents independent research part funded by the Royal College of Psychiatrists' Donald Dean Fellowship and the National Institute for Health Research (NIHR) Biomedical Research Centre at South London and Maudsley NHS Foundation Trust and King's College London (IS-BRC-1215-20018). The views expressed are those of the authors and not necessarily those of the Royal College of Psychiatrists, the NHS, the NIHR or the Department of Health and NIHR Maudsley Biomedical Research Centre, South London and Maudsley NHS Foundation Trust, London, UK.

Acknowledgements: Particular thanks to Megan Pritchard and Amelia Jewell at the NIHR Maudsley Biomedical Research Centre for their support with this study.

Conflict of interests: Professor Matthew Hotopf receives funding from Janssen as part of the RADAR-CNS consortium, which includes a project on depression. He is a principal investigator of RADAR-CNS, a pre-competitive public private partnership co-funded by Innovative Medicines Initiative (European Commission) and European Federation of Pharmaceutical Industries and Associations (EFPIA). He has also been an independent expert witness in group litigations instructed by claimants against pharmaceutical companies for alleged harmful effects of their products.

Authors have no other conflict of interest to declare.

Key words: fit note, health policy, primary care, public health, occupational health

Abstract

Objectives: The fit note replaced the sick note in the UK in 2010, with the aim of improving support for patients requiring sickness absence, yet there has been very little research into fit note use. This study aims to describe number of fit notes by condition, to improve our understanding of patterns of fit note use in primary care. Previous fit note research has relied on extracting diagnoses directly from fit notes, rather than extracting information from clinical records. In this paper we extract information from clinical records to explore demographic factors and conditions associated with number of fit notes issued.

Design: This is a longitudinal study of clinical data. We analysed individual-level anonymised data from general practitioner consultations, including demographic information and condition recorded at first fit note. The latter encompassed diagnoses, individual symptoms and psychosocial issues.

For peer review only

Setting: A database called Lambeth DataNet, containing electronic clinical records on 326,415 adults (ages 16-60) from all 45 general practices within the London borough of Lambeth from 1st Jan 2014 to 30th April 2017.

Participants: Our analytical sample contained 40,698 people with a condition recorded at first fit note.

Primary outcome measure: Predicted number of fit notes in the period Jan 2014-April 2017

Results: Of all studied diagnostic groups, mental illness had the highest number of fit notes (n=3.3; 95% CI 3.1,3.4) after controlling for demographic factors and long-term conditions. The highest predicted number of fit notes for any condition subgroup was among patients presenting for drug and/or alcohol misuse (NFN = 4.5; 95% CI 4.1, 4.8).

Conclusions: For the first time, we show drug and/or alcohol misuse at first fit note are associated with the highest number of fit notes. Research is needed to understand the trajectories of individuals at highest risk of long-term sickness absence, in particular, people presenting with drug and/or alcohol misuse.

Strengths and limitations of this study

- This is the largest study to analyse fit note use and clinical information from GP records at point of first fit note, using individual-level clinical data; a major strength of using clinical records data is that they contain more detail on condition type than information reported on the fit note which previous research has relied on.
- The study was conducted in a single geographical area for which we had GP data; this had the benefit of providing information on virtually all individuals seeking help within that population, but it had the disadvantage of a possible loss of generalisability, and our findings should be tested in other areas.
- We do not have access to the information written on the fit note to compare it with the information recorded in clinical notes, and we do not know the length of time that each fit note was prescribed for.
- Each of the condition groups and subgroups encompasses a very wide variety of disorders.

For peer review only

- There is additional information that would have been extremely useful, such as nature of employment, educational level, occupational status or benefit use, which were not available in this dataset.

Introduction

In the UK long term sickness absence is a major driver of inequalities, leading to social exclusion and adverse health outcomes[1]. The impact of long term sickness absence is usually expressed in financial terms, with an estimated £15bn lost to the economy in the year 2017/18[2]. In 2008, a government report suggested that the process of sickness certification by general practitioners (GPs) was a contributor to the problem of long-term sickness absence, and recommended the introduction of a new form of sickness certification: the fit note[2].

The sick note, used to certify episodes of sickness absence of over 7 days, was replaced by the fit note in 2010[3]. The fit note was designed to reduce long term sickness absence by replacing the sick note's binary 'fit' versus 'not fit to work', with the addition of a third option, 'maybe fit'[2], and encouraging GPs to certify shorter periods of sickness absence, with more frequent reviews. 'Not fit for work' and the new 'maybe fit' option both provide evidence of sickness to an employer and can be used in the administration of sickness benefits. The fit note also includes space for GPs to recommend work adjustments for employees, to encourage employers to adapt to the health needs of workers[4]. The digitisation of the fit note in 2012 created the opportunity for researchers and policy makers to describe patterns of fit note use in the UK population[5-7].

A single fit note following an injury or illness is a common occurrence. A study found that 60% of all fit notes issued in the UK from 2011-13 were single fit notes[8]. In contrast, repeated fit notes are rarer and suggest a more serious injury or chronic illness, or other reasons for not recovering, such as multimorbidity, illness complications or barriers to return to work. Identifying conditions and demographic groups associated with high fit note use enables us to define groups with high levels of need and to inform future interventions.

Mental disorders are the single most common reason for a fit note, making up over 30% of fit notes nationally (NHS Digital 2014-2017)[5]. Research to date has found multiple fit note use to be associated with mental illness and stress-related problems, higher levels of deprivation, minority ethnic groups, and female gender[9-11]. In the majority of fit note research, the reason for sickness absence is taken directly from the fit note, where information is written for the worker, employer and/or the Department of Work and Pensions[12 13]. There are limitations to this approach. Firstly, half of all fit notes do not provide adequate information for the reason for sickness absence to be

For peer review only

coded[14], and secondly, information on the fit note may differ from information in the clinical notes, for reasons such as individual confidentiality or clinicians' preference[13 15 16].

International research suggests that a large proportion of workers presenting for sickness certification have multimorbidity[17 18] ; yet, the extent of multimorbidity is rarely captured on the fit note itself, even though there is no limit to the number of conditions that can be recorded. Even when information about multimorbidity is provided on the fit note, it is often excluded from analysis[8 19].

In this paper we overcome some of the limitations of relying on information provided on the fit note by extracting conditions recorded in clinical records at the point of first fit note receipt from Read codes recorded by general practitioners. Conditions include diagnoses, individual symptoms, psychosocial issues and codes related to the healthcare provided. We explore the relationship between variables known to be associated with fit note receipt and number of fit notes, anticipating that the number of fit notes issued in our sample, between Jan 2014-April 2017, would be higher among individuals with mental disorders, those with multimorbidity, women, those living in areas of greater deprivation, older age groups, and minority ethnic groups. We will test for a multiplier effect of mental health and physical co-morbidity on number of fit notes. We expect demographic associations to be partially explained by long-term conditions and that individuals with infection, injury and obstetric conditions will tend to have a fewer fit notes.

In summary, the first aim of this paper is to describe the predicted number of fit notes by condition at first fit note extracted from clinical notes. The second objective is to describe demographic variation in predicted number of fit notes, lastly we will explore the interaction effects of co-morbid conditions at first fit note on predicted number of fit notes.

Methods

Data

Prospective longitudinal data for 326,415 patients were extracted from electronic clinical records using a database of primary care providers in the London Borough of Lambeth, Lambeth DataNet (LDN)[11]. The London Borough of Lambeth, home to a population of just over 318,000, is the 22nd most deprived local authority in England[20] and has the 4th highest level of income inequality of any borough in London[21]. Lambeth contains a young and ethnically diverse population, including large Portuguese, South American and Black populations[22]. Around 150 different languages are spoken and around 17,000 people (6% of the population) speak a main language other than English[20]. Thirty-eight percent of the population was born outside of the

For peer review only

UK[20]. The number of individuals registered on LDN at any one time (n=405,000) exceeds the total Lambeth population recorded in the Census, due to the cross-boundary registration of individuals not living in Lambeth, GP list inflation[23], and the temporary residence of students in the Lambeth area from surrounding universities. We analysed records covering a period of 40 months from 1st January 2014 to 30th April 2017. Data were extracted in May 2017 from the primary care clinical record system, EMIS Web. We restricted the sample to adults age 16-60 years to exclude exits from the workforce due to reaching statutory pension age (ages 60-65 years), which was not measured in our sample.

Demographic variables

Information on individuals' first recorded sex, age at the beginning of window, ethnicity and lower super output area (LSOA) of residence (an area covering an average population of 1,722[24]) were extracted from the GP records. Ethnicity was coded using seven subcategories: Asian, Black African, Black Caribbean, Black Other, Mixed, Other and White). This uses the five broad groups used in the 2011 census, with the Black group split into sub-groups, following previous research within South London suggesting differences in mental health outcomes between Black Caribbean and Black African groups[25 26]. Deprivation was measured using the Index of Multiple Deprivation (IMD) 2015 rank of their LSOA of residence. IMD scores were divided into quintiles, based on deprivation scores in Lambeth[27].

Fit note

Date of first fit note was derived for each participant based on digital fit notes issued between 1st January 2014 to 30th April 2017. We excluded fit notes issued before this period because although digital fit notes were introduced in 2012, they were not in widespread use until 2014. Due to the lack of information on prior sickness certification (before January 2014) we could not determine whether a 'first fit note' during the study period represented the individual's first ever sickness certification. Number of fit notes are measured as the total number of fit notes received in the study window.

Presenting condition at first fit note

There were a wide variety of conditions (recorded using Read codes) in the electronic GP record when a first fit note was issued. These were split into two broad categories of "Mental Health" and "Non-Mental Health", containing 11 broad groups comprising 37 subgroups. The creation of 11 groups was based on a pragmatic mix of the size of the groups, the way diagnoses are grouped in ICD-10 and past fit note research[28 19 29]. The three Mental Health groups were: mental disorder, stress and specific external stressors (which includes bereavement and relationship breakdown) and the eight Non-Mental Health groups were musculoskeletal, infection, surgery, obstetrics, injury, physical health, fatigue, and a group for 'other' conditions

For peer review only

that could not easily be categorised into one of the other groups (e.g. administrative codes). The broad 'physical health' group was created for individual symptoms that could not be attributed to a single system or group of diagnoses. We defined 37 diagnostic subgroups (Supplementary Figure 1 and Supplementary Table 1). Where a condition fit into more than one diagnostic group, we used a hierarchy to allocate codes ensuring each condition was allocated to a single diagnostic subgroup (see Supplementary Figure 1). For patients with more than one condition at first fit note (14% of people) one condition was selected at random as the primary condition, additional conditions were taken into account in the analysis of multiple symptoms.

Long term conditions and pain

Presence of long-term conditions was assessed in addition to condition at first fit note, using the Quality and Outcomes Framework (QOF), an annual reward and incentive programme for all GP practices in England. QOF measures have been shown to underestimate prevalence of disorders in the general population due to the reliance on the GP for a diagnosis[30]. QOF diagnosis is therefore a specific, but not sensitive measure[31] with a low false-positive but high false-negative rates. In this paper 'long term condition' comprises 15 QOF[32] conditions: depression, epilepsy, diabetes mellitus (DM), coronary heart disease (CHD), chronic obstructive pulmonary disorder (COPD), cancer (non-specified), atrial fibrillation (AF), heart failure (HF), stroke, rheumatoid arthritis, obesity, severe mental illness (SMI; schizophrenia, bipolar disorder), learning disability, hypertension and asthma. QOF conditions excluded from our analysis were smoking (not a long-term condition), palliative care, osteoporosis, dementia and chronic kidney disease (due to the small numbers with these diagnosis in the age group under study). In addition, chronic pain[33] was derived based on receipt (yes/no) of any prescriptions listed in British National Formulary medication chapters 4.7.2 or 4.7.3 (opioid analgesics and neuropathic pain medication) with repeat, repeat dispensed or automatic issue type.

Patient and public involvement: Findings from this study were presented to and discussed with the NIHR Biomedical Research Nucleus Data Linkage Service User and Carer Advisory Group.

Statistical analysis

We undertook a descriptive analysis of demographic variables (age, gender, ethnicity and deprivation), long-term conditions and conditions at first fit note. To assess associations between conditions and fit note counts we used zero-truncated negative binomial regression models. A truncated model was used because all individuals in our sample had at least one fit note to ensure predictions were made on the range $[1, \infty)$ rather than $[0, \infty)$. A negative binomial model was used to account for overdispersion in the number of fit notes. To account for clustering of individuals within GP practices, standard errors were adjusted using a sandwich estimator[34]. Models are presented in terms of model predicted number of fit notes because this is the most

For peer review only

For peer review only - http://bmjopen.bmj.com/site/about/guidelines.xhtml

clinically relevant outcome. The predicted number of fit notes was calculated for each group, holding other variables to their sample mean values. Regarding the difference between predicted and actual counts: these are connected but different. The actual counts are the counts observed in the raw data, without any adjustment or clustering. The predicted counts are derived from the regression model, and as such, account for other variables in the model. In model 1 we adjust for demographic variables: age, gender, ethnicity and deprivation, and in model 2 we adjust for demographic variables and additionally the number of long-term conditions. We did a full case analysis, described in detail in a recent publication[11]. Lastly we test for an interaction between co-morbid conditions and predicted number of fit notes.

For peer review only

Results

Our analytical sample included 40,698 patients with at least one condition code recorded at first fit note, having excluded 284,913 patients without any fit notes, and 804 people with undefined reasons for fit note use, these included administrative codes reported in the clinical record at point of first fit note. Of 40,698 patients, 86% of individuals had one condition code and 14% of people had more than one condition recorded in the clinical notes at first fit note. The number of conditions types varied by age, with 13% of 16-40 year-olds having multiple condition types compared with 17% among 51-60 year-olds ($p<0.001$). The largest group of conditions was the physical health group (31% of all conditions), followed by musculoskeletal (21%), infection (17%) and mental illness (14%).

Table 1 shows that the number of fit notes received varied by age, ethnicity and deprivation, but were similar among women and men. Older age, high deprivation, Black Caribbean, Black Other and Mixed ethnic groups were strongly associated with a higher number of fit notes. Twice as many individuals in the oldest age group (51-60 years) received 4+ fit notes compared to those aged 20-25 years. The percentage of individuals receiving 4+ fit notes was higher among Black Caribbean and Black Other individuals (47% and 45%, compared with 34% or less among Other, Asian and White individuals), and for individuals living in more deprived areas (compared to those living in less deprived areas). These differences remained after adjustment for long-term conditions.

Table 2 and Figure 1 show the number of fit notes by condition at first fit note. Mental illness, stress and musculoskeletal conditions were associated with the highest number of fit notes, after adjusting for demographic factors and long-term conditions. Infection and surgery were associated with the lowest numbers of fit notes. After adjustment for demographic factors and long-term conditions, of the 37 subgroups the highest predicted number of fit notes were for drug

and/or alcohol misuse, common mental disorders, and severe mental illness (Table 2, Supplementary Table 1 and Supplementary Figure 2).

Patients with comorbid mental and physical health conditions had a higher predicted number of fit notes than patients with either mental health or physical health conditions alone. The predicted number of fit notes increased across all condition groups when coupled with a mental health condition but interaction effects reached statistical significance only for mental health conditions co-morbid with infection at first fit note (predicted fit notes for infection 1.8 [1.7,1.8] versus co-morbid infection and mental health condition 3.2 [2.4,4.0] (chi2 3.7 (1) p=0.05), these results are in the text only.

We found no associations between number of condition types and number of fit notes, but we found a higher number of fit notes amongst individuals with mental health conditions than those with non-mental health conditions, independent of the number of condition types recorded at first fit note.

Discussion

We show for the first time that individuals with drug and/or alcohol misuse have the highest number of fit notes of any condition at first fit note. High numbers of fit note represent complexity, chronicity, high service use, longer periods of sickness absence and more frequent primary care reviews. We are aware of one study identifying alcohol and drug misuse recorded on fit notes which found that alcohol and drug misuse was more common in the most socially deprived areas, making up 9% of fit notes for mild-moderate mental health disorders, compared to 1% in the least deprived areas[35]. We anticipate that such analyses of drug and/or alcohol misuse recorded on fit notes (rather than in clinical records as in this study) are likely to lead to an underestimation, because GPs writing fit notes are aware that if they record substance misuse disorders directly on the fit note, it will be seen by the individual's employer[16].

Across the 11 main groups, our findings were consistent with previous studies; mental illness at first fit note was associated with the highest number of fit notes, followed by 'feeling stressed' and 'external stressors'[11 36]. In contrast, infection and minor surgery were associated with the smallest number of fit notes[8]. Evidence suggests that co-existing mental and physical conditions are associated with poorer clinical and functional outcomes and lower quality of life, than physical conditions alone[37-48]. We found that the combination of mental and physical disorders at first fit note increased NFN significantly compared to physical conditions alone, but we did not find a "multiplier effect" of mental health across physical conditions. However, amongst people who

For peer review only

presented with infection and mental health conditions at first fit note, the combination doubled the predicted number of fit notes of infection (p=0.05).

Older individuals, people living in deprived areas and those in Black Other, Black Caribbean, and Mixed groups continue to receive on average a higher number of fit notes in the final fully adjusted model. This remaining association is likely to be explained by exposure to a multitude of disadvantages that we have not been able to measure, such as long term conditions not captured by the QOF, occupational disadvantages such as precarity, high risk occupations (e.g. construction, health and social care)[19] and work place discrimination[49]. Although women are more likely to receive a first fit note, we find that they then received a similar number of fit notes to men.

The largest study of fit notes to date is national reporting on fit notes issued, published by NHS Digital[7]. At first glance, compared to NHS Digital, there appears to be an underrepresentation of mental health in our sample, despite being a population with a high prevalence of mental health problems[50]. The lower prevalence of fit notes for mental health in our sample compared to previous studies is expected because we have extracted diagnosis at first fit note, rather than diagnosis on all fit notes, of which mental health takes up a larger proportion due to the association between mental health conditions and multiple fit note use[14]. Secondly, we know that 1.7-9.6% of non-mental health diagnosis will change to mental health conditions on future fit notes, which we have not captured in our study[8].

In contrast to mental health conditions, infections make up a much higher proportion of first fit notes in our sample than total fit note samples (16.5% at first fit note vs 3.5% of NHS Digital sample). Infection is associated with lower number of fit notes and younger populations, so we expect to see a higher proportion of fit notes for infection at first fit note than found in the NHS digital sample which includes diagnosis on all fit notes, not just the first. In addition, the categorisation of condition types in our study is likely to explain some of the difference: we have categorised conditions using a hierarchy of condition groups, infections were grouped together instead of added to their corresponding system group[28]. Our findings are in keeping with the labour force survey in which ‘minor illnesses’, including infections, were a large contributor to sickness absence[51].

The ‘drug and/or alcohol misuse’ subgroup in our study represents a wide spectrum of conditions, including harmful use and dependence on multiple different substances (Supplementary Table 2). Substance misuse, in particular alcohol misuse, is common and associated with absenteeism[52], and higher risk drinking profiles in early adulthood are an important predictor of receiving a disability pension later in life.[53]. The Alcohol Use Disorders

For peer review only

Identification Test (AUDIT) used in the Adult Psychiatric Morbidity Survey 2014 found that 20% of adults in England drink at hazardous levels and above (AUDIT $\geq$ 8). White men and women are more likely to drink at hazardous, and harmful levels than Black, Asian and minority ethnic groups. Some workforce groups, such as doctors, and male dominated industries such as the military and construction, have been identified as having particularly high levels of alcohol misuse[54-56]. Despite high levels of physical and mental comorbidity, workers with drug and/or alcohol misuse often present late, and struggle to access health services[57]. In addition, in recent years funding for addiction services has been drastically cut in England, and addiction budgets removed from ringfencing protections within the NHS[58 59]. The findings suggest a strong case for employment based treatment services such as the successful in-house Drug and Alcohol Treatment Service developed by Transport for London[60], a major employer in Lambeth.

Patients with multiple fit notes are groups with high service use, chronicity and complexity, who may benefit from earlier intervention and focused occupational health support. We found that communities with highest number of fit note use are at highest risk of insecure work, and workers with mental health conditions, particularly drug and/or alcohol misuse, experience major barriers to work. Countries in which changes have been most successful in tackling exclusion from the workplace due to sickness and disability, include those with systems for early identification of occupational health problems and where employer incentives and legislation have supported workers to exercise their right to remain in work[61]. Further research is needed into access to healthcare and occupational support amongst patients with multiple fit note use.

Strengths and Limitations

This is the largest study to analyse fit note use and clinical information from GP records at point of first fit note, using individual-level clinical data. A major strength of using clinical records data is that they contain more detail on condition type than information reported on the fit note which previous research has relied on. Clinical record data also provided usable information on reason for fit note for 98% of first fit notes, compared to the 50% of NHS Digital fit notes with diagnostic information. The study was limited because we do not have access to the information written on the fit note to compare it with the information recorded in clinical notes, and we do not know the length of time that each fit note was prescribed for, or the number of sickness absence episodes. Therefor we are unable to describe the precise pattern of multiple fit note use. For example, higher numbers of fit notes could be due to longer periods of sickness absence, or more frequent reviews in response to diagnostic uncertainty, negotiation of sickness absence or provision of health care[62]. We cannot differentiate whether a high predicted number of fit notes represent longer sickness absence, a tendency for more frequent review by GPs, or both. We also did not have access to sickness certification before the start of our study window, therefore our results do not represent lifetime first fit note, but first fit note in the study window. Each of the condition

For peer review only

groups and subgroups encompasses a very wide variety of disorders. For example, the subgroup ‘drug and/or alcohol misuse’ is made up of harmful use and dependence diagnoses on multiple different substances (see Supplementary Table 2). The lack of granularity in the ‘drug and/or alcohol’ subgroup, and other groups/subgroups is a limitation.

The study was conducted in a single geographical area for which we had GP data. This had the benefit of providing information on virtually all individuals seeking help within that population, but it had the disadvantage of a possible loss of generalisability, and our findings should be tested in other areas. There is additional information that would have been extremely useful, such as nature of employment, educational level or benefit use, which were not available in this dataset. Without this information it was impossible to ascertain whether fit notes were issued for individuals in work versus those already out of work who were applying for health benefits. Furthermore, we did not know the occupational status of individuals within the population—for example whether they were unemployed, retired or in full-time education.

Conclusions

For the first time, we show drug and/or alcohol misuse at first fit note is associated with the highest number of fit notes, and that there is evidence that co-morbid mental health conditions at first fit note may have a multiplier effect on predicted number of fit notes for individuals presenting with infection. Demographic risk factors for high numbers of fit notes include Black Caribbean, Black Other or Mixed ethnicity, and higher levels of deprivation; these are likely to be explained by risk factors that we have not been able to measure, such as high risk occupations[19] and work place discrimination[49]. An understanding of the predicted number of fit notes by condition and demographic group enables policy makers to evaluate the primary care resources available to working age adults’ at high risk of multiple fit note receipt. Linked occupational and health data sets are needed to better understand the occupational, clinical and wider structural risk factors for high number of fit notes, and to explore the trajectories of individuals after first fit note use.

For peer review only

Contributorship statement: Sarah Dorrington designed the study, analysed the data and drafted the manuscript. Matthew Hotopf, Stephani L. Hatch and Ira Madan advised extensively throughout the process. Ewan Carr advised on the statistical analysis. Catherine Polling advised on the categorisation of read codes into groups and subgroups. Matthew Broadbent, Mark Ashworth and Sharon A.M. Stevelink provided expertise and guidance on extracting, analysing and interpreting the data. Emmert Roberts and all authors commented on the final manuscript.

Ethics approval CRIS was established in 2008 and approved by the Oxfordshire Research Ethics Committee in 2008 (reference 18/SC/0372). Approval for linkage with Lambeth DataNet was granted by Lambeth Clinical Commissioning Group and Information Governance Committee. This project was approved by the CRIS Oversight Committee in 2015. All patients have the choice to opt-out of their anonymised data being used.

Data sharing statement Data may be obtained from a third party and are not publicly available. Access to the deidentified data in this study can be applied for through Lambeth Datanet and South London and Maudsley Biomedical Research Centre (BRC), De Crespigny Park, London SE5 8AF (cris.administrator@slam.nhs.uk). Access is granted only if approval is given by both the BRC and the Lambeth Datanet Steering Group.

Conflict of interests: Professor Matthew Hotopf receives funding from Janssen as part of the RADAR-CNS consortium, which includes a project on depression. He is a principal investigator of RADAR-CNS, a pre-competitive public private partnership co-funded by Innovative Medicines Initiative (European Commission) and European Federation of Pharmaceutical Industries and Associations (EFPIA). He has also been an independent expert witness in group litigations instructed by claimants against pharmaceutical companies for alleged harmful effects of their products. Authors have no other conflict of interest to declare.

Funding: This project was funded by the Royal College of Psychiatrists' Donald Dean Fellowship and the National Institute for Health Research Award IS-BRC-1215-20018.

Figure Legends:

Figure 1: Percentage of patients with 1, 2, 3 or 4+ fit notes by condition at first fit note

Supplementary Figure 1: Classification of conditions into sub-groups, groups and categories

Supplementary Figure 2: Percentage of patients with 1, 2, 3 or 4+ fit notes by symptom at first fit note

For peer review only

References

1. Henderson M, Clark C, Stansfeld S, et al. A lifecourse approach to long-term sickness absence-a Cohort study. *PLoS ONE* 2012;7(5):e36645. doi: 10.1371/journal.pone.0036645
2. Health and safety at work: summary statistics for Great Britain 2019: Health and Safety Executive, 2019.
3. Coggon D, Palmer KT. Assessing fitness for work and writing a “fit note”. *BMJ (Clinical research ed)* 2010;341:c6305.
4. Thornhill S, Williams N. Completion of the statement of fitness for work: concise guidance. *Clin Med (Lond)* 2012;12(1):63-6. [published Online First: 2012/03/01]
5. Digital N. Fit notes issued by GP practices : December 2014 - March 2017 : experimental statistics. 2017
6. Gabbay MB. Electronic fit notes: sickness certification in the new decade. *Br J Gen Pract* 2010;60(573):235-6. doi: 10.3399/bjgp10X483841
7. NHSDigital. Fit notes issued by GP practices, England. December 2014 - March 2017 2017 [Available from: https://digital.nhs.uk/data-and-information/publications/statistical/fit-notes-issued-by-gp-practices/fit-notes-issued-by-gp-practices-england-december-2014-march-2017.
8. Shiels C, Gabbay MB, Hillage J. Change in diagnosis on fit notes within an episode of certified sickness absence. *Family Practice* 2016;33(5):510-16.
9. Dorrington S, Roberts E, Mykletun A, et al. Systematic review of fit note use for workers in the UK. *Occupational and environmental medicine* 2018 doi: 10.1136/oemed-2017-104730 [published Online First: 2018/05/08]
10. Gabbay M, Shiels C, Hillage J. Factors associated with the length of fit note-certified sickness episodes in the UK. *Occupational and environmental medicine* 2015;72(7):467-75. doi: 10.1136/oemed-2014-102307 [published Online First: 2015/02/26]
11. Dorrington S, Carr E, Stevelink SAM, et al. Demographic variation in fit note receipt and long-term conditions in south London. *Occupational & Environmental Medicine* 2020 doi: 10.1136/oemed-2019-106035 [published Online First: 2020/03/27]
12. Coole C, Potgieter I, Nouri F, et al. Return-to-work outcomes and usefulness of actual fit notes received by employers. *Family Practice* 2015;32(5):551-56.
13. Hussey S, Hoddinott P, Wilson P, et al. Sickness certification system in the United Kingdom: qualitative study of views of general practitioners in Scotland. *BMJ (Clinical research ed)* 2004;328(7431):88. doi: 10.1136/bmj.37949.656389.EE
14. Digital N. Fit Notes Issued by GP Practices 2020 [Available from: https://digital.nhs.uk/data-and-information/publications/statistical/fit-notes-issued-by-gp-practices/guide#csv-metadata accessed April 2020.
15. Rachman R, Bunce D, Thorley K, et al. Patients' attitudes to sickness certification in general practice. *Occupational medicine (Oxford, England)* 2015;65(6):485-8. doi: 10.1093/occmed/kqv069 [published Online First: 2015/06/17]

For peer review only

16. Fletcher-Brown R. 'Mind Your Own Business' - how can we best support employers to improve the mental health of their employees? *Perspect Public Heal* 2015;135(1):18-20.
17. Buist-Bouwman MA, de Graaf R, Vollebergh WA, et al. Comorbidity of physical and mental disorders and the effect on work-loss days. *Acta Psychiatr Scand* 2005;111(6):436-43. doi: 10.1111/j.1600-0447.2005.00513.x [published Online First: 2005/05/10]
18. Dorrington S, Carr E, Stevelink SAM, et al. Multimorbidity and fit note receipt in working-age adults with long-term health conditions. *Psychol Med* 2020:1-10. doi: 10.1017/S0033291720002937 [published Online First: 2020/09/09]
19. Starzmann K, Hjerpe P, Dalemo S, et al. Diagnoses have the greatest impact on variation in sick-leave certification rate among primary-care patients in Sweden: A multilevel analysis including patient, physician and primary health-care centre levels. *Scand J Public Health* 2015;43(7):704-12. doi: 10.1177/1403494815591898 [published Online First: 2015/07/01]
20. Lambeth Council: State of the Borough. Lambeth Council, 2016.
21. Overview of London Boroughs. https://www.trustforlondon.org.uk/data/boroughs/overview-of-london-boroughs/. : Trust for London: Tackling poverty and inequality, 2019.
22. Mixed and balanced communities: Census Information Service, Greater London Authority, London, 2014.
23. Patient Register: quality assurance of administrative data used in population statistics: Office for National Statistics; 2016 [Available from: https://www.ons.gov.uk/peoplepopulationandcommunity/populationandmigration/populationestimates/methodologies/patientregisterqualityassuranceofadministrativedatausedinpopulationstatisticsdec2016.
24. LSOA Atlas. https://data.london.gov.uk/dataset/lsoa-atlas: Greater London Authority, 2014.
25. 2011 Census analysis: Ethnicity and religion of the non-UK born population in England and Wales: 2011. In: Statistics OoN, ed. https://www.ons.gov.uk/peoplepopulationandcommunity/culturalidentity/ethnicity/articles/2011censusanalysisethnicityandreligionofthenonukbornpopulationinenglandandwales/2015-06-18, 2011.
26. Hatch SL, Frissa S, Verdecchia M, et al. Identifying socio-demographic and socioeconomic determinants of health inequalities in a diverse London community: the South East London Community Health (SELCoH) study. *BMC Public Health* 2011;11:861. doi: 10.1186/1471-2458-11-861 [published Online First: 2011/11/15]
27. The English Index of Multiple Deprivation (IMD) 2015. In: Government DfCaL, ed. https://assets.publishing.service.gov.uk/government/uploads/system/uploads/attachment_data/file/464430/English_Index_of_Multiple_Deprivation_2015_-_Guidance.pdf, 2015.
28. Shiels C, Gabbay MB, Hillage J. Change in diagnosis on fit notes within an episode of certified sickness absence. *Fam Pract* 2016;33(5):510-6. doi: 10.1093/fampra/cmw050 [published Online First: 2016/06/28]
29. Shiels C, Gabbay M, Hillage J. Factors associated with prevalence and types of 'may be fit' advice on fit notes: a cross-sectional primary care analysis. *Br J Gen Pract* 2014;64(620):e137-43. doi: 10.3399/bjgp14X677509 [published Online First: 2014/02/26]

30. The King's Fund. Impact of Quality and Outcomes Framework on health inequalities. 2011
31. Economou A, Grey M, McGregor J, et al. The health informatics cohort enhancement project (HICE): using routinely collected primary care data to identify people with a lifetime diagnosis of psychotic disorder. *BMC Research Notes* 2012;5:95. doi: 10.1186/1756-0500-5-95 [published Online First: 2012/02/16]
32. QOF Business Rules - version 35. Department of Health 2017.
33. From One to Many: Exploring people's progression to multiple long-term conditions in an urban environment https://www.gsttcharity.org.uk/what-we-do/our-programmes/multiple-long-term-conditions/one-many-exploring-peoples-progression2018 [
34. Rogers W. Regression standard errors in clustered samples. *Stata Technical Bulletin* 1994;3(13)
35. Shiels CH, J; Pollard E; Gabbay, M. Evaluation of the statement of fitness for work (fit note) : quantitative survey of fit notes. *Research report* 2013(no 841):95 pages.
36. Gabbay M, Shiels C, Hillage J. Sickness certification for common mental disorders and GP return-to-work advice. *Primary Health Care Research Development* 2016:1-11. doi: 10.1017/s1463423616000074 [published Online First: 2016/03/11]
37. Scott KM, Bruffaerts R, Tsang A, et al. Depression-anxiety relationships with chronic physical conditions: results from the World Mental Health Surveys. *Journal of Affective Disorders* 2007;103(1-3):113-20. doi: 10.1016/j.jad.2007.01.015 [published Online First: 2007/02/13]
38. Teesson M, Mitchell PB, Deady M, et al. Affective and anxiety disorders and their relationship with chronic physical conditions in Australia: findings of the 2007 National Survey of Mental Health and Wellbeing. *Australian and New Zealand Journal of Psychiatry* 2011;45(11):939-46. doi: 10.3109/00048674.2011.614590 [published Online First: 2011/10/05]
39. Teesson M, Slade T, Mills K. Comorbidity in Australia: findings of the 2007 National Survey of Mental Health and Wellbeing. *Aust N Z J Psychiatry* 2009;43(7):606-14. doi: 10.1080/00048670902970908 [published Online First: 2009/06/17]
40. Li C, Ford ES, Zhao G, et al. Prevalence and correlates of undiagnosed depression among U.S. adults with diabetes: the Behavioral Risk Factor Surveillance System, 2006. *Diabetes Res Clin Pract* 2009;83(2):268-79. doi: 10.1016/j.diabres.2008.11.006 [published Online First: 2008/12/30]
41. Das-Munshi J, Stewart R, Ismail K, et al. Diabetes, common mental disorders, and disability: findings from the UK National Psychiatric Morbidity Survey. *Psychosomatic Medicine* 2007;69(6):543-50. doi: 10.1097/PSY.0b013e3180cc3062 [published Online First: 2007/07/20]
42. Tsang A, Von Korff M, Lee S, et al. Common chronic pain conditions in developed and developing countries: gender and age differences and comorbidity with depression-anxiety disorders. *J Pain* 2008;9(10):883-91. doi: 10.1016/j.jpain.2008.05.005 [published Online First: 2008/07/08]
43. Moussavi S, Chatterji S, Verdes E, et al. Depression, chronic diseases, and decrements in health: results from the World Health Surveys. *Lancet* 2007;370(9590):851-8. doi: 10.1016/S0140-6736(07)61415-9 [published Online First: 2007/09/11]

For peer review only

44. Evans DL, Charney DS, Lewis L, et al. Mood disorders in the medically ill: scientific review and recommendations. *Biol Psychiatry* 2005;58(3):175-89. doi: 10.1016/j.biopsych.2005.05.001 [published Online First: 2005/08/09]
45. Whooley MA, Caska CM, Hendrickson BE, et al. Depression and inflammation in patients with coronary heart disease: findings from the Heart and Soul Study. *Biol Psychiatry* 2007;62(4):314-20. doi: 10.1016/j.biopsych.2006.10.016 [published Online First: 2007/04/17]
46. Chapman DP, Perry GS, Strine TW. The vital link between chronic disease and depressive disorders. *Prev Chronic Dis* 2005;2(1):A14. [published Online First: 2005/01/27]
47. Woodhead C, Ashworth M, Schofield P, et al. Patterns of physical co-/multi-morbidity among patients with serious mental illness: a London borough-based cross-sectional study. *BMC Family Practice* 2014;15:117. doi: 10.1186/1471-2296-15-117 [published Online First: 2014/06/13]
48. Making more of multimorbidity: an emerging priority. *The Lancet* 2018;391(10131):1637. doi: 10.1016/s0140-6736(18)30941-3
49. Hatch SL, Gazard B, Williams DR, et al. Discrimination and common mental disorder among migrant and ethnic groups: findings from a South East London Community sample. *Soc Psychiatry Psychiatr Epidemiol* 2016 doi: 10.1007/s00127-016-1191-x
50. Hatch SL, Woodhead C, Frissa S, et al. Importance of thinking locally for mental health: data from cross-sectional surveys representing South East London and England. *PLoS One* 2012;7(12):e48012. doi: 10.1371/journal.pone.0048012 [published Online First: 2012/12/20]
51. Labour Force Survey: Sickness absence in the UK labour market 2018 GovUK, 2018.
52. Roche A, Pidd K, Kostadinov V. Alcohol- and drug-related absenteeism: a costly problem. *Aust N Z J Public Health* 2016;40(3):236-8. doi: 10.1111/1753-6405.12414 [published Online First: 2015/08/12]
53. Bockerman P, Hyytinen A, Maczulskij T. Devil in disguise: Does drinking lead to a disability pension? *Prev Med* 2016;86:130-5. doi: 10.1016/j.ypmed.2016.03.008 [published Online First: 2016/03/13]
54. Jones E, Fear NT. Alcohol use and misuse within the military: a review. *Int Rev Psychiatry* 2011;23(2):166-72. doi: 10.3109/09540261.2010.550868 [published Online First: 2011/04/28]
55. Fowlie DG. The misuse of alcohol and other drugs by doctors: a UK report and one region's response. *Alcohol Alcohol* 1999;34(5):666-71. doi: 10.1093/alcalc/34.5.666 [published Online First: 1999/10/21]
56. Flannery J, Ajayi SO, Oyegoke AS. Alcohol and substance misuse in the construction industry. *Int J Occup Saf Ergon* 2019:1-16. doi: 10.1080/10803548.2019.1601376 [published Online First: 2019/04/03]
57. Brooke DE, Griffiths; Taylor, Colin. Addiction as an occupational hazard. *British journal of addiction* 1991
58. Drummond C. Cuts to addiction services are a false economy. *BMJ : British Medical Journal* 2017
59. Roberts E, Hotopf M, Drummond C. The relationship between alcohol-related hospital admission and specialist alcohol treatment provision across local authorities in England since passage of the Health and Social Care Act 2012. *Br J Psychiatry* 2020:1-3. doi: 10.1192/bjp.2020.120 [published Online First: 2020/07/02]

For peer review only

60. Ballard J. TfL's Drug and Alcohol Assessment and Treatment Service. *Occupational Health at Work* 2019;15/5:36-38.
61. Hemmings P. Sickness and disability systems: comparing outomes and policies in Norway with those in Sweden, Netherlands and Switzerland. 2020
62. Byrne P, Ring A, Salmon P, et al. Tussles and Rollovers: Negotiating Sickness Certification in Primary Care. *Advances in Applied Sociology* 2014;Vol.04No.12:14. doi: 10.4236/aasoci.2014.412029

For peer review only

Health condition at first fit note and number of fit notes: evidence from a large primary care sample in south London

Table 1 Number of fit notes received and predicted number of fit notes by sociodemographic variables

Demographic variables	Number of fit notes received: 1st January 2014 to 30th April 2017 n (%)				Predicted NFN unadjusted (95%CI)	Predicted number of fit notes (95% CI)	
	1	2	3	4+		Model 1: Adjusted for age, gender, ethnicity and deprivation	Model 2: +long-term conditions
Whole pop				40,698	2.0 [1.8,2.1]	2.2 [2.1,2.3]	2.4 [2.3,2.5]
Gender							
Male	6,097 (35.2)	3,014 (17.4)	1,878 (10.9)	6,312 (36.5)	2.0 [1.8,2.2]	2.2 [2.1,2.3]	2.4 [2.3,2.6]
Female	8,304 (35.5)	4,231 (18.1)	2,557 (10.9)	8,305 (35.5)	1.9 [1.8,2.1]	2.2 [2.1,2.3]	2.3 [2.2,2.4]
Age group							
16-20	428 (46.9)	178 (19.5)	80 (8.8)	227 (24.9)	1.4 [1.2,1.6]	1.4 [1.2,1.6]	1.7 [1.5,1.9]
21-25	2,054 (49.3)	807 (19.4)	379 (9.1)	926 (22.2)	1.3 [1.2,1.5]	1.4 [1.3,1.5]	1.7 [1.5,2.8]
36-30	2,825 (46.0)	1,138 (18.5)	650 (10.6)	1,532 (24.9)	1.5 [1.4,1.6]	1.6 [1.4,1.7]	1.8 [2.7,2.9]
31-35	2,311 (38.7)	1,136 (19.0)	691 (11.6)	1,839 (30.8)	1.8 [1.7,2.0]	1.9 [2.8,2.0]	2.2 [2.1,2.3]
36-40	1,771 (34.3)	996 (19.3)	591 (11.5)	1,803 (34.9)	2.1 [2.0,2.3]	2.2 [2.0,2.3]	2.4 [2.3,2.5]
41-45	1,541 (31.2)	881 (17.8)	532 (10.8)	1,985 (40.2)	2.4 [2.3,2.6]	2.5 [2.3,2.6]	2.6 [2.4,2.7]
46-50	1,528 (28.1)	880 (16.2)	594 (10.9)	2,437 (44.8)	2.7 [2.5,2.9]	2.7 [2.6,2.9]	2.7 [2.5,2.8]
51-55	1,226 (26.2)	709 (15.1)	536 (11.4)	2,218 (47.3)	2.9 [2.7,3.1]	2.9 [1.8,3.1]	2.8 [2.6,2.9]
56-60	717 (21.9)	520 (15.9)	382 (11.7)	1,650 (50.5)	3.1 [2.9,3.3]	3.2 [3.0,3.4]	2.9 [2.7,3.0]
Ethnicity							
White	8,592 (39.4)	3,979 (18.2)	2,264 (10.4)	6,980 (32.0)	1.8 [1.6,2.0]	2.0 [1.9,2.1]	2.2 [2.1,2.3]
Black African	1,889 (32.4)	1,090 (18.7)	650 (11.1)	2,211 (37.9)	2.1 [2.0,2.3]	2.2 [2.1,2.4]	2.4 [2.2,2.5]
Asian	845 (35.5)	430 (18.1)	292 (12.3)	815 (34.2)	1.9 [1.7,2.2]	2.2 [1.9,2.4]	2.3 [2.1,2.5]
Black Caribbean	1,279 (25.4)	809 (16.1)	567 (11.3)	2,380 (47.3)	2.7 [2.5,2.9]	2.8 [2.7,2.9]	2.9 [2.8,3.0]
Mixed	774 (31.5)	404 (16.5)	300 (12.2)	978 (39.8)	2.2 [2.1,2.4]	2.6 [2.4,2.8]	2.7 [2.6,2.9]
Other	503 (36.2)	268 (19.3)	167 (12.0)	450 (32.4)	1.9 [1.7,2.1]	2.0 [1.8,2.2]	2.3 [2.1,2.5]
Black Other	519 (29.1)	265 (14.9)	195 (10.9)	803 (45.1)	2.6 [2.4,2.8]	2.8 [2.6,3.0]	2.9 [2.7,3.2]
Deprivation							
1	2,325 (39.8)	1,142 (19.5)	626 (10.7)	1,750 (30.0)	1.7 [1.5,1.8]	1.9 [1.8,2.0]	2.0 [2.0,2.2]
2	2,575 (38.1)	1,265 (18.1)	705 (10.4)	2,254 (33.3)	1.8 [1.7,2.0]	2.1 [2.9,2.2]	2.1 [2.1,2.4]
3	2,725 (37.6)	1,411 (17.1)	823 (11.4)	2,836 (33.9)	1.9 [1.7,2.1]	2.1 [2.0,2.3]	2.1 [2.1,2.4]
4	3,067 (32.7)	1,606 (17.8)	1,033 (11.0)	3,521 (38.5)	2.2 [2.0,2.3]	2.3 [2.2,2.5]	2.4 [2.4,2.6]
5	3,353 (32.1)	1,817 (17.1)	1,134 (10.9)	4,195 (39.9)	2.2 [2.1,2.4]	2.4 [2.3,2.6]	2.6 [2.4,2.7]

*mutually adjusted for age, gender, ethnicity and deprivation **mutually adjusted as above, also adjusted for number of long-term conditions (QOF)

For peer review only

Table 2 Predicted number of fit notes by diagnostic group

Category	Groups	Selected sub-groups	N people (%)	Predicted number of fit notes (NFN) [95%CI]	Predicted NFN adj* [95%CI]	Predicted NFN adj** [95%CI]
Mental Health	Total		40,698	2.0 [1.8,2.1]	2.2 [2.1,2.3]	2.4 [2.3,2.5]
	Mental illness***		5,841 (14.4)	2.8 [2.6,3.0]	3.3 [3.1,3.5]	3.3 [3.1,3.4]
		Drug and alcohol	584 (1.4)	3.9 [3.6,4.3]	4.2 [3.8,4.6]	4.5 [4.1,4.8]
		Common Mental Disorders	4,393(10.8)	2.7 [2.5,2.9]	3.2 [3.1,3.4]	3.2 [3.1,3.3]
		Severe Mental Illness	374 (0.9)	2.7 [2.5,3.0]	3.0 [2.8,3.3]	2.6 [2.4,2.8]
	Stress		1,037 (2.6)	2.1 [1.8,2.3]	2.5 [2.2,2.7]	2.7 [2.4,2.9]
	Stress Ext		845 (2.1)	2.2 [1.9,2.4]	2.3 [2.1,2.5]	2.5 [2.3,2.7]
Non Mental Health	MSK		8,366 (20.6)	2.2 [2.1,2.4]	2.3 [2.2,2.5]	2.6 [2.4,2.7]
	Fatigue		541 (1.3)	1.9 [1.6,2.2]	2.2 [2.0,2.5]	2.4 [2.1,2.7]
	Injury		2,183 (5.4)	1.8 [1.6,1.9]	2.1 [1.9,2.2]	2.3 [2.2,2.5]
	Physical health		12,710 (31.2)	2.1 [1.9,2.3]	2.2 [2.1,2.3]	2.3 [2.2,2.4]
	Other		448 (1.1)	2.0 [1.8,2.3]	2.2 [2.0,2.4]	2.3 [2.1,2.5]
	Obstetrics		1,372 (3.4)	1.4 [1.3,1.6]	2.0 [1.8,2.2]	2.2 [2.0,2.4]
	Infection		6,726 (16.5)	1.5 [1.3,1.6]	1.8 [1.7,1.9]	1.9 [1.8,2.0]
	Surgery		629 (1.6)	1.5 [1.3,1.8]	1.6 [1.4,1.9]	1.8 [1.5,2.1]

*adjusted for age, gender, ethnicity and deprivation
**adjusted for age, gender, ethnicity, deprivation and number of long-term conditions
***made up of drug & alcohol, common mental disorders, severe mental illness, mental health treatment and mental health other

For peer review only

For peer review only

Figure 1

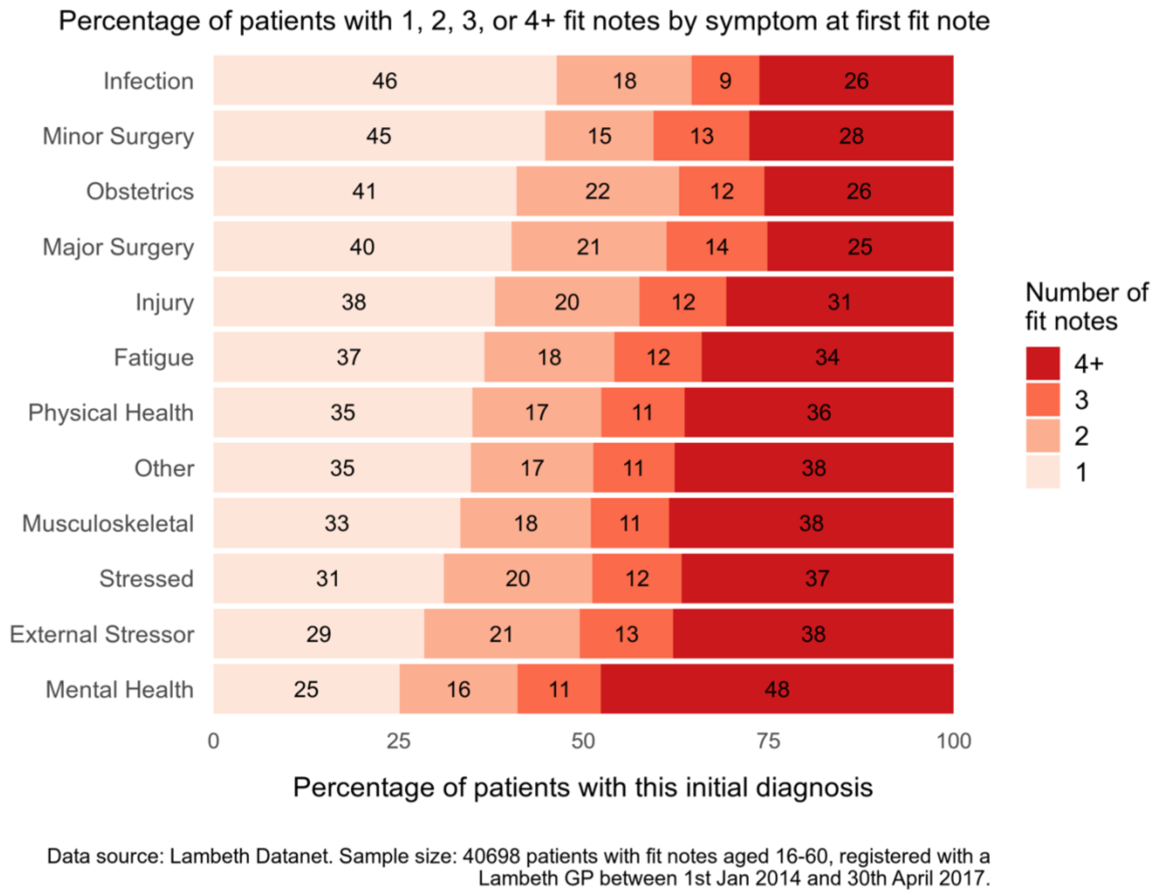
Percentage of patients with 1, 2, 3, or 4+ fit notes by symptom at first fit note
Infection
46
18
9
26
Minor Surgery
45
15
13
28
Obstetrics
41
22
12
26
Major Surgery
40
21
14
25
Injury
38
20
12
31
Fatigue
37
18
12
34
Physical Health
35
17
11
36
Other
35
17
11
38
Musculoskeletal
33
18
11
38
Stressed
31
20
12
37
External Stressor
29
21
13
38
Mental Health
25
16
11
48
0
25
50
75
100
Percentage of patients with this initial diagnosis
Number of fit notes
4+
3
2
1
Data source: Lambeth Datanet. Sample size: 40698 patients with fit notes aged 16-60, registered with a Lambeth GP between 1st Jan 2014 and 30th April 2017.

For peer review only

SUPPLEMENTARY MATERIAL TABLES

Table 1 Predicted number of fit notes by condition type

Condition sub-group	Number (%)	Predicted number of fit notes (NFN)	Predicted NFN adj*	Predicted NFN adj**
Drug and alcohol	584 (1.4)	3.9 (3.6-4.3)	4.2 (3.8-4.6)	4.5 (4.1-4.8)
Other Mental Health Problem	294 (0.7)	2.7 (2.3-3.0)	3.7 (3.2-4.2)	3.6 (3.1-4.0)
Common Mental Disorders	4,393(10.8)	2.7 (2.5-2.9)	3.2 (3.1-3.4)	3.2 (3.1-3.3)
Severe Mental Illness	374 (0.9)	2.7 (2.5-3.0)	3.0 (2.8-3.3)	2.6 (2.4-2.8)
Neurology	640 (1.6)	2.5 (2.2-2.8)	2.8 (2.5-3.1)	2.8 (2.5-3.1)
Haematology	134 (0.3)	2.5 (2.1-2.9)	2.8 (2.3-3.3)	3.2 (2.6-3.8)
Obesity	74 (0.2)	2.7 (2.0-3.3)	2.7 (2.0-3.4)	2.4 (1.8-2.9)
Insomnia	174 (0.4)	2.2 (1.8-2.7)	2.6 (2.0-3.2)	2.7 (2.1-3.3)
Mental Health Treatment	192 (0.5)	2.3 (1.7-2.8)	2.6 (2.0-3.1)	2.4 (1.9-2.9)
Cancer	239 (0.6)	2.5 (2.3-2.8)	2.5 (2.2-2.7)	2.5 (2.2-2.7)
Stressed	1,039 (2.6)	2.1 (1.9-2.3)	2.5 (2.2-2.7)	2.7 (2.5-2.9)
Diabetes	58 (0.1)	2.8 (2.0-3.6)	2.5 (1.8-3.2)	2.1 (1.5-2.7)
Systemic Illness	1,028 (2.5)	2.6 (2.3-2.8)	2.4 (2.2-2.6)	2.1 (1.9-2.3)
Physical symptom	1,439 (3.5)	2.2 (2.0-2.4)	2.4 (2.2-2.5)	2.5 (2.3-2.6)
Other	326 (0.8)	2.2 (1.9-2.5)	2.4 (2.1-2.6)	2.5 (2.2-2.7)
External stressor	349 (0.9)	2.1 (1.8-2.5)	2.4 (2.0-2.7)	2.6 (2.2-2.9)
Musculoskeletal	8,288 (20.4)	2.3 (2.1 2.4)	2.3 (2.2-2.5)	2.6 (2.4-2.7)
Bereavement	495 (1.2)	2.2 (1.9-2.5)	2.3 (2.1-2.6)	2.5 (2.3-2.8)
Test	26 (0.1)	2.7 (1.7-3.7)	2.3 (1.6-3.1)	2.3 (1.6-3.0)
Cardiovascular	1,234 (3.0)	2.5 (2.2-2.7)	2.2 (2.1-2.4)	2.2 (2.0-2.3)
Gastroenterology	2,241 (5.5)	2.0 (1.8-2.1)	2.2 (2.1-2.4)	2.4 (2.3-2.6)
Gynaecology	768 (1.9)	1.9 (1.7-2.0)	2.2 (2.0-2.3)	2.4 (2.3-2.6)
Dermatology	1,129 (2.8)	1.9 (1.6-2.1)	2.2 (1.9-2.4)	2.3 (2.1-2.6)
Respiratory	1,663 (4.1)	2.0 (1.8-2.2)	2.1 (2.0-2.3)	2.0 (1.9-2.2)
Injury	2,183 (5.4)	1.8 (1.6-2.0)	2.1 (1.9-2.3)	2.4 (2.2-2.5)
Fatigue	367 (0.9)	1.8 (1.5-2.1)	2.1 (1.8-2.4)	2.3 (2.0-2.6)
Physical Health Treatment	13 (0.0)	1.9 (1.0-2.9)	2.1 (1.0-3.2)	2.1 (0.8-3.3)
Genitourinary Medicine/Urology	481 (1.2)	2.0 (1.7-2.3)	2.0 (1.8-2.3)	2.1 (1.9-2.4)
Obstetrics	1,371 (3.4)	1.4 (1.3-1.6)	2.0 (1.8-2.2)	2.2 (2.0-2.5)
Ear, Nose, Throat and Dental	1,016 (2.5)	1.8 (1.6-2.1)	2.0 (1.7-2.2)	2.1 (1.9-2.4)
Wound	562 (1.4)	1.8 (1.6-2.0)	1.9 (1.8-2.1)	2.1 (1.9-2.3)
Infection	6,727 (16.5)	1.5 (1.4-1.7)	1.8 (1.7-1.9)	1.9 (1.8-2.0)
Risk factor	33 (0.1)	1.8 (1.1-2.5)	1.8 (1.2-2.3)	1.8 (1.2-2.4)
Minor Surgery	232 (0.6)	1.6 (1.3-1.9)	1.7 (1.4-2.0)	1.9 (1.5-2.3)
Allergy	88 (0.2)	1.4 (1.0-1.9)	1.7 (1.2-2.2)	1.9 (1.3-2.4)
Major Surgery	397 (1.0)	1.6 (1.3-1.8)	1.6 (1.4-1.9)	1.8 (1.5-2.0)
Renal	47 (0.1)	1.6 (0.8-2.3)	1.6 (0.9-2.4)	1.9 (1.0-2.7)

*adjusted for age, gender, ethnicity and deprivation

**adjusted for age, gender, ethnicity, deprivation and number of long term condition

Table 2 Example of subgroup codes:

Drug and Alcohol Codes:
[RFC] Drug/ substance abuse
[RFC] Substance abuse
[X]Alcohol addiction
[X]Drug addiction - cannabis
[X]Heroin addiction
[X]Men & behav disorder multiple drug use/psychoactive subst
[X]Mental and behav dis due to use alcohol: dependence syndr
[X]Mental and behav dis due to use cannabinoids: harmful use
[X]Mental and behav dis due to use of alcohol: harmful use
[X]Mental and behav dis due to use of cocaine: harmful use
[X]Mental and behav dis due to use of opioids: harmful use
[X]Mental and behav dis due to use opioids: dependence syndr
[X]Mental behav disorders due use crack cocaine: harmful use
Alcohol dependence syndrome
Alcohol dependence syndrome NOS
Alcohol misuse - enhanced service completed
Alcohol problem drinking
Alcohol withdrawal syndrome
Alcoholism
Analgesic overuse headache
Benzodiazepine dependence
Cannabis dependence, continuous
Cannabis dependence, unspecified
Cannabis type drug dependence
Chronic alcoholism
Chronic alcoholism in remission
Cocaine dependence, episodic
Cocaine dependence, unspecified
Cocaine drug dependence NOS
Cocaine type drug dependence
Diazepam dependence
Drug addiction
Drug addiction therapy
Drug addictn therap-methadone
Drug dependence
Drug dependence NOS
Drug dependence therapy
Drug seeking behaviour
Drug withdrawal syndrome
Drunkenness NOS
Hangover (alcohol)
Harmful alcohol use
Hazardous alcohol use
Heavy drinker - 7-9u/day
Heroin dependence
Intoxication - alcohol
Methadone dependence
Misuse of drugs NOS
Morphine dependence
Nondependent alcohol abuse
Nondependent cannabis abuse
Opioid drug dependence NOS
Opioid type drug dependence
Refer to drugs service
Very heavy drinker - >9u/day

For peer review only

Supplementary Figure 1 Classification of conditions into sub-groups, groups and categories

**Categories**	**Groups**	**Sub-groups**
Non-Mental Health	Injury*	Injury*
	Infection*	Infection*
	Surgery*	Minor Surgery*
		Major Surgery*
	Fatigue/Insomnia	Insomnia
		Fatigue
	Obstetrics	Obstetrics
	Musculoskeletal	Musculoskeletal
	Physical Health	Cancer
		Neurology
		Haematology
		Obesity
		Test
		Cardiovascular
		Gastroenterology
		Gynaecology
		Dermatology
		Respiratory
		Physical Health Treatment
		Genitourinary Medicine/Urology
		Allergy
		Risk factor
		Ear, Nose, Throat and Dental
		Renal
		Diabetes
		Systemic Illness
		Physical symptom
		Wound
	Other	Other Physical Health
Mental Health	Mental Illness	Common Mental Disorders
		Severe Mental Illness
		Drug and Alcohol
		Mental Health Treatment
		Other Mental Health Problem**
	External Stressor	Bereavement
		External stressor
	Stressed	Stressed

*Injury, infection and surgery were at the top of the two-tier hierarchy of groupings, as an alternative to assigning injuries, infections and surgery to their system groups.
** Other mental health problem includes self-harm, developmental disorders, personality disorder

Supplementary Figure 2: Percentage of patients with 1, 2, 3 or 4+ fit notes by symptom at first fit note

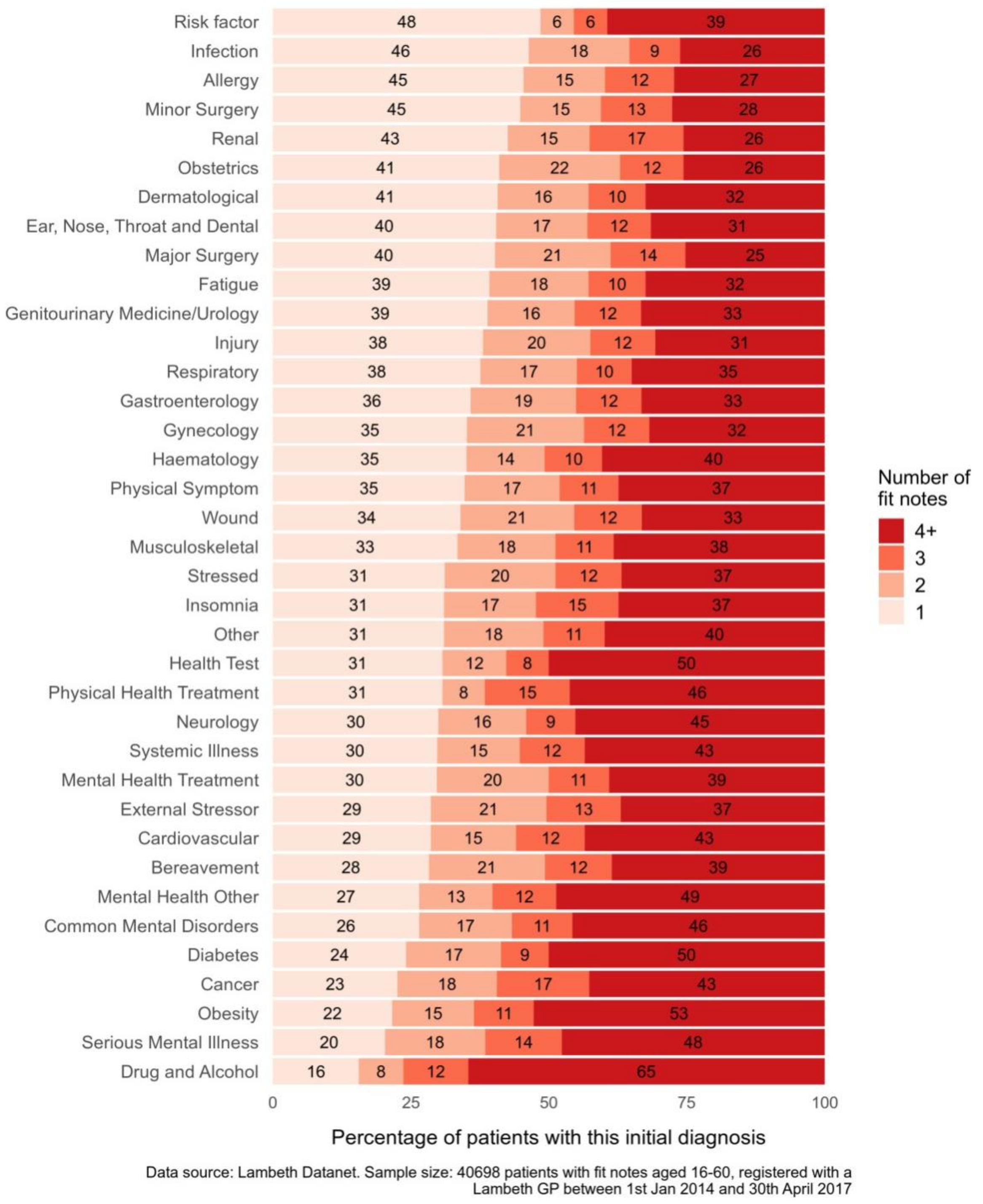

Reporting checklist for cohort study.

Based on the STROBE cohort guidelines.

Instructions to authors

Complete this checklist by entering the page numbers from your manuscript where readers will find each of the items listed below.

Your article may not currently address all the items on the checklist. Please modify your text to include the missing information. If you are certain that an item does not apply, please write "n/a" and provide a short explanation.

Upload your completed checklist as an extra file when you submit to a journal.

In your methods section, say that you used the STROBE cohortreporting guidelines, and cite them as:

von Elm E, Altman DG, Egger M, Pocock SJ, Gotzsche PC, Vandenbroucke JP. The Strengthening the Reporting of Observational Studies in Epidemiology (STROBE) Statement: guidelines for reporting observational studies.

For peer review only

		Reporting Item	Page Number
**Title and abstract**			
Title	#1a	Indicate the study's design with a commonly used term in the title or the abstract	1
Abstract	#1b	Provide in the abstract an informative and balanced summary of what was done and what was found	3
**Introduction**			
Background / rationale	#2	Explain the scientific background and rationale for the investigation being reported	4
Objectives	#3	State specific objectives, including any prespecified hypotheses	4
**Methods**			
Study design	#4	Present key elements of study design early in the paper	5
Setting	#5	Describe the setting, locations, and relevant dates, including periods of	5

		recruitment, exposure, follow-up, and data collection	
Eligibility criteria	#6a	Give the eligibility criteria, and the sources and methods of selection of participants. Describe methods of follow-up.	6
Eligibility criteria	#6b	For matched studies, give matching criteria and number of exposed and unexposed	n/a
Variables	#7	Clearly define all outcomes, exposures, predictors, potential confounders, and effect modifiers. Give diagnostic criteria, if applicable	6 and 7
Data sources / measurement	#8	For each variable of interest give sources of data and details of methods of assessment (measurement). Describe comparability of assessment methods if there is more than one group. Give information separately for for exposed and unexposed groups if applicable.	6 and 7
Bias	#9	Describe any efforts to address potential sources of bias	11
Study size	#10	Explain how the study size was arrived at	7
Quantitative variables	#11	Explain how quantitative variables were handled in the analyses. If applicable, describe which groupings were chosen, and why	6
Statistical methods	#12a	Describe all statistical methods, including those used to control for confounding	7
Statistical methods	#12b	Describe any methods used to examine subgroups and interactions	7
Statistical methods	#12c	Explain how missing data were addressed	7
Statistical methods	#12d	If applicable, explain how loss to follow-up was addressed	n/a
Statistical methods	#12e	Describe any sensitivity analyses	n/a
**Results**			
Participants	#13a	Report numbers of individuals at each stage of study—eg numbers potentially eligible, examined for eligibility, confirmed eligible, included in the study, completing follow-up, and analysed. Give information separately for for exposed and unexposed groups if applicable.	7

For peer review only

Participants	#13b	Give reasons for non-participation at each stage	7
Participants	#13c	Consider use of a flow diagram	n/a
Descriptive data	#14a	Give characteristics of study participants (eg demographic, clinical, social) and information on exposures and potential confounders. Give information separately for exposed and unexposed groups if applicable.	7
Descriptive data	#14b	Indicate number of participants with missing data for each variable of interest	7
Descriptive data	#14c	Summarise follow-up time (eg, average and total amount)	na
Outcome data	#15	Report numbers of outcome events or summary measures over time. Give information separately for exposed and unexposed groups if applicable.	7 and 8
Main results	#16a	Give unadjusted estimates and, if applicable, confounder-adjusted estimates and their precision (eg, 95% confidence interval). Make clear which confounders were adjusted for and why they were included	7
Main results	#16b	Report category boundaries when continuous variables were categorized	7
Main results	#16c	If relevant, consider translating estimates of relative risk into absolute risk for a meaningful time period	na
Other analyses	#17	Report other analyses done—e.g., analyses of subgroups and interactions, and sensitivity analyses	9
**Discussion**			
Key results	#18	Summarise key results with reference to study objectives	9
Limitations	#19	Discuss limitations of the study, taking into account sources of potential bias or imprecision. Discuss both direction and magnitude of any potential bias.	11
Interpretation	#20	Give a cautious overall interpretation considering objectives, limitations, multiplicity of analyses, results from similar studies, and other relevant evidence.	11
Generalisability	#21	Discuss the generalisability (external validity) of the study results	11
**Other Information**			

For peer review only

Funding	#22	Give the source of funding and the role of the funders for the present study and, if applicable, for the original study on which the present article is based	1

The STROBE checklist is distributed under the terms of the Creative Commons Attribution License CC-BY. This checklist was completed on 16. August 2020 using https://www.goodreports.org/, a tool made by the EQUATOR Network in collaboration with Penelope.ai

For peer review only

BMJ Open

Health condition at first fit note and number of fit notes: a longitudinal study of primary care records in south London

Journal:	*BMJ Open*
Manuscript ID	bmjopen-2020-043889.R2
Article Type:	Original research
Date Submitted by the Author:	27-Feb-2021
Complete List of Authors:	Dorrington, Sarah; King's College London Institute of Psychiatry Psychology and Neuroscience, Department of Psychological Medicine; South London and Maudsley NHS Foundation Trust, NIHR Maudsley Biomedical Research Centre<br>Carr, Ewan ; King's College London Institute of Psychiatry Psychology and Neuroscience, Department of Psychological Medicine<br>Polling, C; King's College London Institute of Psychiatry Psychology and Neuroscience, Department of Psychological Medicine; South London and Maudsley Mental Health NHS Trust, NIHR Maudsley Biomedical Research Centre<br>Stevelink, Sharon; King's College London Institute of Psychiatry Psychology and Neuroscience, Department of Psychological Medicine<br>Ashworth, Mark; King's College London, School of Population Health & Environmental Sciences<br>Roberts , Emmert; King's College London Institute of Psychiatry Psychology and Neuroscience, National Addiction Centre; South London and Maudsley Mental Health NHS Trust, NIHR Maudsley Biomedical Research Centre<br>Broadbent, Matthew; South London and Maudsley Mental Health NHS Trust, NIHR Maudsley Biomedical Research Centre<br>HATCH, STEPHANI; King's College London Institute of Psychiatry Psychology and Neuroscience, Department of Psychological Medicine<br>MADAN, IRA; Guy's and St Thomas' Hospitals NHS Trust, Department of Occupational Health<br>Hotopf, Matthew; King's College London Institute of Psychiatry Psychology and Neuroscience, Department of Psychological Medicine; South London and Maudsley Mental Health NHS Trust, NIHR Maudsley Biomedical Research Centre
**Primary Subject Heading**:	General practice / Family practice
Secondary Subject Heading:	Health policy, Health services research, Epidemiology, Occupational and environmental medicine, Public health
Keywords:	PRIMARY CARE, PUBLIC HEALTH, OCCUPATIONAL & INDUSTRIAL MEDICINE, Health policy < HEALTH SERVICES ADMINISTRATION & MANAGEMENT

SCHOLARONE™
Manuscripts

I, the Submitting Author has the right to grant and does grant on behalf of all authors of the Work (as defined in the below author licence), an exclusive licence and/or a non-exclusive licence for contributions from authors who are: i) UK Crown employees; ii) where BMJ has agreed a CC-BY licence shall apply, and/or iii) in accordance with the terms applicable for US Federal Government officers or employees acting as part of their official duties; on a worldwide, perpetual, irrevocable, royalty-free basis to BMJ Publishing Group Ltd ("BMJ") its licensees and where the relevant Journal is co-owned by BMJ to the co-owners of the Journal, to publish the Work in this journal and any other BMJ products and to exploit all rights, as set out in our licence.

The Submitting Author accepts and understands that any supply made under these terms is made by BMJ to the Submitting Author unless you are acting as an employee on behalf of your employer or a postgraduate student of an affiliated institution which is paying any applicable article publishing charge ("APC") for Open Access articles. Where the Submitting Author wishes to make the Work available on an Open Access basis (and intends to pay the relevant APC), the terms of reuse of such Open Access shall be governed by a Creative Commons licence – details of these licences and which Creative Commons licence will apply to this Work are set out in our licence referred to above.

Other than as permitted in any relevant BMJ Author's Self Archiving Policies, I confirm this Work has not been accepted for publication elsewhere, is not being considered for publication elsewhere and does not duplicate material already published. I confirm all authors consent to publication of this Work and authorise the granting of this licence.

For peer review only

Health condition at first fit note and number of fit notes: a longitudinal study of primary care records in south London

Sarah Dorrington (1,2)*
Ewan Carr (1)
Catherine Polling (1,2)
Sharon A.M. Stevelink (1)
Mark Ashworth (3)
Emmert Roberts (1,2)
Matthew Broadbent (2)
Stephani L Hatch (1)
Ira Madan (4)
Matthew Hotopf (1,2)

(1) Department of Psychological Medicine, Institute of Psychiatry, Psychology & Neuroscience King's College London, 16 De Crespigny Park, London SE5 8AF
(2) NIHR Maudsley Biomedical Research Centre, South London and Maudsley NHS Foundation Trust, London, UK
(3) Department of Population Health Sciences, School of Population Health and Environmental Sciences, King's College London, Guy's Campus, Addison House, London SE1 1UL
(4) Department of Occupational Health, Guy's and St Thomas' NHS Foundation Trust, London, UK

Word count: 3508

*Corresponding author.
3rd Floor, Institute of Psychiatry, Psychology and Neuroscience, De Crespigny Park, London SE5 8AF; Sarah.dorrington@kcl.ac.uk; 02078485817.

For peer review only

Abstract

Objectives: The fit note replaced the sick note in the UK in 2010, with the aim of improving support for patients requiring sickness absence, yet there has been very little research into fit note use. This study aims to describe number of fit notes by condition, to improve our understanding of patterns of fit note use in primary care. Previous fit note research has relied on extracting diagnoses directly from fit notes, rather than extracting information from clinical records. In this paper we extract information from clinical records to explore demographic factors and conditions associated with number of fit notes issued.

Design: This is a longitudinal study of clinical data. We analysed individual-level anonymised data from general practitioner consultations, including demographic information and condition recorded at first fit note. The latter encompassed diagnoses, individual symptoms and psychosocial issues.

Setting: A database called Lambeth DataNet, containing electronic clinical records on 326,415 adults (ages 16-60) from all 45 general practices within the London borough of Lambeth from 1st Jan 2014 to 30th April 2017.

Participants: Our analytical sample contained 40,698 people with a condition recorded at first fit note.

Primary outcome measure: Predicted number of fit notes in the period Jan 2014-April 2017

Results: Of all studied diagnostic groups, mental illness had the highest number of fit notes (n=3.3; 95% CI 3.1,3.4) after controlling for demographic factors and long-term conditions. The highest predicted number of fit notes for any condition subgroup was among patients presenting for drug and/or alcohol misuse (NFN = 4.5; 95% CI 4.1, 4.8).

Conclusions: For the first time, we show drug and/or alcohol misuse at first fit note are associated with the highest number of fit notes. Research is needed to understand the trajectories of individuals at highest risk of long-term sickness absence, in particular, people presenting with drug and/or alcohol misuse.

Strengths and limitations of this study

For peer review only

- This is the largest study to analyse fit note use and clinical information from GP records at point of first fit note, using individual-level clinical data; a major strength of using clinical records data is that they contain more detail on condition type than information reported on the fit note which previous research has relied on.
- The study was conducted in a single geographical area for which we had GP data; this had the benefit of providing information on virtually all individuals seeking help within that population, but it had the disadvantage of a possible loss of generalisability, and our findings should be tested in other areas.
- We do not have access to the information written on the fit note to compare it with the information recorded in clinical notes, and we do not know the length of time that each fit note was prescribed for.
- Each of the condition groups and subgroups encompasses a very wide variety of disorders.
- There is additional information that would have been extremely useful, such as nature of employment, educational level, occupational status or benefit use, which were not available in this dataset.

Introduction

In the UK long term sickness absence is a major driver of inequalities, leading to social exclusion and adverse health outcomes[1]. The impact of long term sickness absence is usually expressed in financial terms, with an estimated £15bn lost to the economy in the year 2017/18[2]. In 2008, a government report suggested that the process of sickness certification by general practitioners (GPs) was a contributor to the problem of long-term sickness absence, and recommended the introduction of a new form of sickness certification: the fit note[2].

The sick note, used to certify episodes of sickness absence of over 7 days, was replaced by the fit note in 2010[3]. The fit note was designed to reduce long term sickness absence by replacing the sick note's binary 'fit' versus 'not fit to work', with the addition of a third option, 'maybe fit'[2], and encouraging GPs to certify shorter periods of sickness absence, with more frequent reviews. 'Not fit for work' and the new 'maybe fit' option both provide evidence of sickness to an employer and can be used in the administration of sickness benefits. The fit note also includes space for GPs to recommend work adjustments for employees, to encourage employers to adapt to the health needs of workers[4]. The digitisation of the fit note in 2012 created the opportunity for researchers and policy makers to describe patterns of fit note use in the UK population[5-7].

A single fit note following an injury or illness is a common occurrence. A study found that 60% of all fit notes issued in the UK from 2011-13 were single fit notes[8]. In contrast, repeated fit notes

For peer review only

are rarer and suggest a more serious injury or chronic illness, or other reasons for not recovering, such as multimorbidity, illness complications or barriers to return to work. Identifying conditions and demographic groups associated with high fit note use enables us to define groups with high levels of need and to inform future interventions.

Mental disorders are the single most common reason for a fit note, making up over 30% of fit notes nationally (NHS Digital 2014-2017)[5]. Research to date has found multiple fit note use to be associated with mental illness and stress-related problems, higher levels of deprivation, minority ethnic groups, and female gender[9-11]. In the majority of fit note research, the reason for sickness absence is taken directly from the fit note, where information is written for the worker, employer and/or the Department of Work and Pensions[12 13]. There are limitations to this approach. Firstly, half of all fit notes do not provide adequate information for the reason for sickness absence to be coded[14], and secondly, information on the fit note may differ from information in the clinical notes, for reasons such as individual confidentiality or clinicians' preference[13 15 16].

International research suggests that a large proportion of workers presenting for sickness certification have multimorbidity[17 18] ; yet, the extent of multimorbidity is rarely captured on the fit note itself, even though there is no limit to the number of conditions that can be recorded. Even when information about multimorbidity is provided on the fit note, it is often excluded from analysis[8 19].

In this paper we overcome some of the limitations of relying on information provided on the fit note by extracting conditions recorded in clinical records at the point of first fit note receipt from Read codes recorded by general practitioners. Conditions include diagnoses, individual symptoms, psychosocial issues and codes related to the healthcare provided. We explore the relationship between variables known to be associated with fit note receipt and number of fit notes, anticipating that the number of fit notes issued in our sample, between Jan 2014-April 2017, would be higher among individuals with mental disorders, those with multimorbidity, women, those living in areas of greater deprivation, older age groups, and minority ethnic groups. We will test for a multiplier effect of mental health and physical co-morbidity on number of fit notes. We expect demographic associations to be partially explained by long-term conditions and that individuals with infection, injury and obstetric conditions will tend to have a fewer fit notes.

In summary, the first aim of this paper is to describe the predicted number of fit notes by condition at first fit note extracted from clinical notes. The second objective is to describe demographic variation in predicted number of fit notes. Lastly we will explore the interaction effects of co-morbid conditions at first fit note on predicted number of fit notes.

For peer review only

Methods

Data

Prospective longitudinal data for 326,415 patients were extracted from electronic clinical records using a database of primary care providers in the London Borough of Lambeth, Lambeth DataNet (LDN)[11]. The London Borough of Lambeth, home to a population of just over 318,000, is the 22nd most deprived local authority in England[20] and has the 4th highest level of income inequality of any borough in London[21]. Lambeth contains a young and ethnically diverse population, including large Portuguese, South American and Black populations[22]. Around 150 different languages are spoken and around 17,000 people (6% of the population) speak a main language other than English[20]. Thirty-eight percent of the population was born outside of the UK[20]. The number of individuals registered on LDN at any one time (n=405,000) exceeds the total Lambeth population recorded in the Census, due to the cross-boundary registration of individuals not living in Lambeth, GP list inflation[23], and the temporary residence of students in the Lambeth area from surrounding universities. We analysed records covering a period of 40 months from 1st January 2014 to 30th April 2017. Data were extracted in May 2017 from the primary care clinical record system, EMIS Web. We restricted the sample to adults age 16-60 years to exclude exits from the workforce due to reaching statutory pension age (ages 60-65 years), which was not measured in our sample.

Demographic variables

Information on individuals' first recorded sex, age at the beginning of window, ethnicity and lower super output area (LSOA) of residence (an area covering an average population of 1,722[24]) were extracted from the GP records. Ethnicity was coded using seven subcategories: Asian, Black African, Black Caribbean, Black Other, Mixed, Other and White). This uses the five broad groups used in the 2011 census, with the Black group split into sub-groups, following previous research within South London suggesting differences in mental health outcomes between Black Caribbean and Black African groups[25 26]. Deprivation was measured using the Index of Multiple Deprivation (IMD) 2015 rank of their LSOA of residence. IMD scores were divided into quintiles, based on deprivation scores in Lambeth[27].

Fit note

Date of first fit note was derived for each participant based on digital fit notes issued between 1st January 2014 to 30th April 2017. We excluded fit notes issued before this period because although digital fit notes were introduced in 2012, they were not in widespread use until 2014. Due to the lack of information on prior sickness certification (before January 2014) we could not determine whether a 'first fit note' during the study period represented the individual's first ever

For peer review only

sickness certification. Number of fit notes are measured as the total number of fit notes received in the study window.

Presenting condition at first fit note

There were a wide variety of conditions (recorded using Read codes) in the electronic GP record when a first fit note was issued. These were split into two broad categories of “Mental Health” and “Non-Mental Health”, containing 11 broad groups comprising 37 subgroups. The creation of 11 groups was based on a pragmatic mix of the size of the groups, the way diagnoses are grouped in ICD-10 and past fit note research[28 19 29]. The three Mental Health groups were: mental disorder, stress and specific external stressors (which includes bereavement and relationship breakdown) and the eight Non-Mental Health groups were musculoskeletal, infection, surgery, obstetrics, injury, physical health, fatigue, and a group for ‘other’ conditions that could not easily be categorised into one of the other groups (e.g. administrative codes). The broad ‘physical health’ group was created for individual symptoms that could not be attributed to a single system or group of diagnoses. We defined 37 diagnostic subgroups (Supplementary Figure 1 and Supplementary Table 1). Where a condition fit into more than one diagnostic group, we used a hierarchy to allocate codes ensuring each condition was allocated to a single diagnostic subgroup (see Supplementary Figure 1). For patients with more than one condition at first fit note (14% of people) one condition was selected at random as the primary condition, additional conditions were taken into account in the analysis of multiple symptoms.

Long term conditions and pain

Presence of long-term conditions was assessed in addition to condition at first fit note, using the Quality and Outcomes Framework (QOF), an annual reward and incentive programme for all GP practices in England. QOF measures have been shown to underestimate prevalence of disorders in the general population due to the reliance on the GP for a diagnosis[30]. QOF diagnosis is therefore a specific, but not sensitive measure[31] with a low false-positive but high false-negative rates. In this paper ‘long term condition’ comprises 15 QOF[32] conditions: depression, epilepsy, diabetes mellitus (DM), coronary heart disease (CHD), chronic obstructive pulmonary disorder (COPD), cancer (non-specified), atrial fibrillation (AF), heart failure (HF), stroke, rheumatoid arthritis, obesity, severe mental illness (SMI; schizophrenia, bipolar disorder), learning disability, hypertension and asthma. QOF conditions excluded from our analysis were smoking (not a long-term condition), palliative care, osteoporosis, dementia and chronic kidney disease (due to the small numbers with these diagnosis in the age group under study). In addition, chronic pain[33] was derived based on receipt (yes/no) of any prescriptions listed in British National Formulary medication chapters 4.7.2 or 4.7.3 (opioid analgesics and neuropathic pain medication) with repeat, repeat dispensed or automatic issue type.

For peer review only

Patient and public involvement: Findings from this study were presented to and discussed with the NIHR Biomedical Research Nucleus Data Linkage Service User and Carer Advisory Group.

Statistical analysis

We undertook a descriptive analysis of demographic variables (age, gender, ethnicity and deprivation), long-term conditions and conditions at first fit note. To assess associations between conditions and fit note counts we used zero-truncated negative binomial regression models. A truncated model was used because all individuals in our sample had at least one fit note to ensure predictions were made on the range $[1, \infty)$ rather than $[0, \infty)$. A negative binomial model was used to account for overdispersion in the number of fit notes. To account for clustering of individuals within GP practices, standard errors were adjusted using a sandwich estimator[34]. Models are presented in terms of model predicted number of fit notes because this is the most clinically relevant outcome. The predicted number of fit notes was calculated for each group, holding other variables to their sample mean values. Regarding the difference between predicted and actual counts: these are connected but different. The actual counts are the counts observed in the raw data, without any adjustment or clustering. The predicted counts are derived from the regression model, and as such, account for other variables in the model. In model 1 we adjust for demographic variables: age, gender, ethnicity and deprivation, and in model 2 we adjust for demographic variables and additionally the number of long-term conditions. We did a full case analysis, described in detail in a recent publication[11]. Lastly, we test for an interaction between co-morbid conditions and predicted number of fit notes.

Results

Our analytical sample included 40,698 patients with at least one condition code recorded at first fit note, having excluded 284,913 patients without any fit notes, and 804 people with undefined reasons for fit note use, these included administrative codes reported in the clinical record at point of first fit note. Of 40,698 patients, 86% of individuals had one condition code and 14% of people had more than one condition recorded in the clinical notes at first fit note. The number of conditions types varied by age, with 13% of 16-40 year-olds having multiple condition types compared with 17% among 51-60 year-olds ($p<0.001$). The largest group of conditions was the physical health group (31% of all conditions), followed by musculoskeletal (21%), infection (17%) and mental illness (14%).

Table 1 shows that the number of fit notes received varied by age, ethnicity and deprivation, but were similar among women and men. Older age, high deprivation, Black Caribbean, Black Other and Mixed ethnic groups were strongly associated with a higher number of fit notes. Twice as

For peer review only

many individuals in the oldest age group (51-60 years) received 4+ fit notes compared to those aged 20-25 years. The percentage of individuals receiving 4+ fit notes was higher among Black Caribbean and Black Other individuals (47% and 45%, compared with 34% or less among Other, Asian and White individuals), and for individuals living in more deprived areas (compared to those living in less deprived areas). These differences remained after adjustment for long-term conditions.

Table 2 and Figure 1 show the number of fit notes by condition at first fit note. Mental illness, stress and musculoskeletal conditions were associated with the highest number of fit notes, after adjusting for demographic factors and long-term conditions. Infection and surgery were associated with the lowest numbers of fit notes. After adjustment for demographic factors and long-term conditions, of the 37 subgroups the highest predicted number of fit notes were for drug and/or alcohol misuse, common mental disorders, and severe mental illness (Table 2, Supplementary Table 1 and Supplementary Figure 2).

Patients with comorbid mental and physical health conditions had a higher predicted number of fit notes than patients with either mental health or physical health conditions alone. The predicted number of fit notes increased across all condition groups when coupled with a mental health condition but interaction effects reached statistical significance only for mental health conditions co-morbid with infection at first fit note (predicted fit notes for infection 1.8 [1.7,1.8] versus co-morbid infection and mental health condition 3.2 [2.4,4.0] (chi2 3.7 (1) p=0.05), these results are in the text only.

We found no associations between number of condition types and number of fit notes, but we found a higher number of fit notes amongst individuals with mental health conditions than those with non-mental health conditions, independent of the number of condition types recorded at first fit note.

Discussion

We show for the first time that individuals with drug and/or alcohol misuse have the highest number of fit notes of any condition at first fit note. High numbers of fit note represent complexity, chronicity, high service use, longer periods of sickness absence and more frequent primary care reviews. We are aware of one study identifying alcohol and drug misuse recorded on fit notes which found that alcohol and drug misuse was more common in the most socially deprived areas, making up 9% of fit notes for mild-moderate mental health disorders, compared to 1% in the least deprived areas[35]. We anticipate that such analyses of drug and/or alcohol misuse recorded on fit notes (rather than in clinical records as in this study) are likely to lead to an

For peer review only

underestimation, because GPs writing fit notes are aware that if they record substance misuse disorders directly on the fit note, it will be seen by the individual's employer[16].

Across the 11 main groups, our findings were consistent with previous studies; mental illness at first fit note was associated with the highest number of fit notes, followed by 'feeling stressed' and 'external stressors'[11 36]. In contrast, infection and minor surgery were associated with the smallest number of fit notes[8]. Evidence suggests that co-existing mental and physical conditions are associated with poorer clinical and functional outcomes and lower quality of life, than physical conditions alone[37-48]. We found that the combination of mental and physical disorders at first fit note increased NFN significantly compared to physical conditions alone, but we did not find a "multiplier effect" of mental health across physical conditions. However, amongst people who presented with infection and mental health conditions at first fit note, the combination doubled the predicted number of fit notes of infection (p=0.05).

Older individuals, people living in deprived areas and those in Black Other, Black Caribbean, and Mixed groups continue to receive on average a higher number of fit notes in the final fully adjusted model. This remaining association is likely to be explained by exposure to a multitude of disadvantages that we have not been able to measure, such as long term conditions not captured by the QOF, occupational disadvantages such as precarity, high risk occupations (e.g. construction, health and social care)[19] and work place discrimination[49]. Although women are more likely to receive a first fit note, we find that they then received a similar number of fit notes to men.

The largest study of fit notes to date is national reporting on fit notes issued, published by NHS Digital[7]. At first glance, compared to NHS Digital, there appears to be an underrepresentation of mental health in our sample, despite being a population with a high prevalence of mental health problems[50]. The lower prevalence of fit notes for mental health in our sample compared to previous studies is expected because we have extracted diagnosis at first fit note, rather than diagnosis on all fit notes, of which mental health takes up a larger proportion due to the association between mental health conditions and multiple fit note use[14]. Secondly, we know that 1.7-9.6% of non-mental health diagnosis will change to mental health conditions on future fit notes, which we have not captured in our study[8].

In contrast to mental health conditions, infections make up a much higher proportion of first fit notes in our sample than total fit note samples (16.5% at first fit note vs 3.5% of NHS Digital sample). Infection is associated with lower number of fit notes and younger populations, so we expect to see a higher proportion of fit notes for infection at first fit note than found in the NHS digital sample which includes diagnosis on all fit notes, not just the first. In addition, the

categorisation of condition types in our study is likely to explain some of the difference: we have categorised conditions using a hierarchy of condition groups, infections were grouped together instead of added to their corresponding system group[28]. Our findings are in keeping with the labour force survey in which 'minor illnesses', including infections, were a large contributor to sickness absence[51].

The 'drug and/or alcohol misuse' subgroup in our study represents a wide spectrum of conditions, including harmful use and dependence on multiple different substances (Supplementary Table 2). Substance misuse, in particular alcohol misuse, is common and associated with absenteeism[52], and higher risk drinking profiles in early adulthood are an important predictor of receiving a disability pension later in life.[53]. The Alcohol Use Disorders Identification Test (AUDIT) used in the Adult Psychiatric Morbidity Survey 2014 found that 20% of adults in England drink at hazardous levels and above (AUDIT $\geq$ 8). White men and women are more likely to drink at hazardous, and harmful levels than Black, Asian and minority ethnic groups. Some workforce groups, such as doctors, and male dominated industries such as the military and construction, have been identified as having particularly high levels of alcohol misuse[54-56]. Despite high levels of physical and mental comorbidity, workers with drug and/or alcohol misuse often present late, and struggle to access health services[57]. In addition, in recent years funding for addiction services has been drastically cut in England, and addiction budgets removed from ringfencing protections within the NHS[58 59]. The findings suggest a strong case for employment based treatment services such as the successful in-house Drug and Alcohol Treatment Service developed by Transport for London[60], a major employer in Lambeth.

Patients with multiple fit notes are groups with high service use, chronicity and complexity, who may benefit from earlier intervention and focused occupational health support. We found that communities with highest number of fit note use are at highest risk of insecure work, and workers with mental health conditions, particularly drug and/or alcohol misuse, experience major barriers to work. Countries in which changes have been most successful in tackling exclusion from the workplace due to sickness and disability, include those with systems for early identification of occupational health problems and where employer incentives and legislation have supported workers to exercise their right to remain in work[61]. Further research is needed into access to healthcare and occupational support amongst patients with multiple fit note use.

Strengths and Limitations

This is the largest study to analyse fit note use and clinical information from GP records at point of first fit note, using individual-level clinical data. A major strength of using clinical records data is that they contain more detail on condition type than information reported on the fit note which previous research has relied on. Clinical record data also provided usable information on reason

For peer review only - http://bmjopen.bmj.com/site/about/guidelines.xhtml

for fit note for 98% of first fit notes, compared to the 50% of NHS Digital fit notes with diagnostic information. The study was limited because we do not have access to the information written on the fit note to compare it with the information recorded in clinical notes, and we do not know the length of time that each fit note was prescribed for, or the number of sickness absence episodes. Therefore we are unable to describe the precise pattern of multiple fit note use. For example, higher numbers of fit notes could be due to longer periods of sickness absence, or more frequent reviews in response to diagnostic uncertainty, negotiation of sickness absence or provision of health care[62]. We cannot differentiate whether a high predicted number of fit notes represent longer sickness absence, a tendency for more frequent review by GPs, or both. We also did not have access to sickness certification before the start of our study window, therefore our results do not represent lifetime first fit note, but first fit note in the study window. Each of the condition groups and subgroups encompasses a very wide variety of disorders. For example, the subgroup 'drug and/or alcohol misuse' is made up of harmful use and dependence diagnoses on multiple different substances (see Supplementary Table 2). The lack of granularity in the 'drug and/or alcohol' subgroup, and other groups/subgroups is a limitation.

The study was conducted in a single geographical area for which we had GP data. This had the benefit of providing information on virtually all individuals seeking help within that population, but it had the disadvantage of a possible loss of generalisability, and our findings should be tested in other areas. There is additional information that would have been extremely useful, such as nature of employment, educational level or benefit use, which were not available in this dataset. Without this information it was impossible to ascertain whether fit notes were issued for individuals in work versus those already out of work who were applying for health benefits. Furthermore, we did not know the occupational status of individuals within the population—for example whether they were unemployed, retired or in full-time education.

For peer review only

Conclusions

For the first time, we show drug and/or alcohol misuse at first fit note is associated with the highest number of fit notes, and that there is evidence that co-morbid mental health conditions at first fit note may have a multiplier effect on predicted number of fit notes for individuals presenting with infection. Demographic risk factors for high numbers of fit notes include Black Caribbean, Black Other or Mixed ethnicity, and higher levels of deprivation; these are likely to be explained by risk factors that we have not been able to measure, such as high risk occupations[19] and work place discrimination[49]. An understanding of the predicted number of fit notes by condition and demographic group enables policy makers to evaluate the primary care resources available to working age adults at high risk of multiple fit note receipt. Linked occupational and

health data sets are needed to better understand the occupational, clinical and wider structural risk factors for high number of fit notes, and to explore the trajectories of individuals after first fit note use.

For peer review only

Contributorship statement: Sarah Dorrington designed the study, analysed the data and drafted the manuscript. Matthew Hotopf, Stephani L. Hatch and Ira Madan advised extensively throughout the process. Ewan Carr advised on the statistical analysis. Catherine Polling advised on the categorisation of read codes into groups and subgroups. Matthew Broadbent, Mark Ashworth and Sharon A.M. Stevelink provided expertise and guidance on extracting, analysing and interpreting the data. Emmert Roberts and all authors commented on the final manuscript.

Acknowledgements: Particular thanks to Megan Pritchard and Amelia Jewell at the NIHR Maudsley Biomedical Research Centre for their support with this study.

Ethics approval CRIS was established in 2008 and approved by the Oxfordshire Research Ethics Committee in 2008 (reference 18/SC/0372). Approval for linkage with Lambeth DataNet was granted by Lambeth Clinical Commissioning Group and Information Governance Committee. This project was approved by the CRIS Oversight Committee in 2015. All patients have the choice to opt-out of their anonymised data being used.

Data sharing statement Data may be obtained from a third party and are not publicly available. Access to the deidentified data in this study can be applied for through Lambeth Datanet and South London and Maudsley Biomedical Research Centre (BRC), De Crespigny Park, London SE5 8AF (cris.administrator@slam.nhs.uk). Access is granted only if approval is given by both the BRC and the Lambeth Datanet Steering Group.

Conflict of interests: Professor Matthew Hotopf receives funding from Janssen as part of the RADAR-CNS consortium, which includes a project on depression. He is a principal investigator of RADAR-CNS, a pre-competitive public private partnership co-funded by Innovative Medicines Initiative (European Commission) and European Federation of Pharmaceutical Industries and Associations (EFPIA). He has also been an independent expert witness in group litigations instructed by claimants against pharmaceutical companies for alleged harmful effects of their products. Authors have no other conflict of interest to declare.

Funding statement: This paper represents independent research part funded by the Royal College of Psychiatrists' Donald Dean Fellowship and the National Institute for Health Research (NIHR) Biomedical Research Centre at South London and Maudsley NHS Foundation Trust and King's College London (IS-BRC-1215-20018). The views expressed are those of the authors and not necessarily those of the Royal College of Psychiatrists, the NHS, the NIHR or the Department of Health and NIHR Maudsley Biomedical Research Centre, South London and Maudsley NHS Foundation Trust, London, UK.

For peer review only

Figure Legends:

Figure 1: Percentage of patients with 1, 2, 3 or 4+ fit notes by condition at first fit note

Supplementary Figure 1: Classification of conditions into sub-groups, groups and categories

Supplementary Figure 2: Percentage of patients with 1, 2, 3 or 4+ fit notes by symptom at first fit note

We used the STROBE cohort checklist when writing our report.

For peer review only

References

1. Henderson M, Clark C, Stansfeld S, et al. A lifecourse approach to long-term sickness absence-a Cohort study. *PLoS ONE* 2012;7(5):e36645. doi: 10.1371/journal.pone.0036645
2. Health and safety at work: summary statistics for Great Britain 2019: Health and Safety Executive, 2019.
3. Coggon D, Palmer KT. Assessing fitness for work and writing a “fit note”. *BMJ (Clinical research ed)* 2010;341:c6305.
4. Thornhill S, Williams N. Completion of the statement of fitness for work: concise guidance. *Clin Med (Lond)* 2012;12(1):63-6. [published Online First: 2012/03/01]
5. Digital N. Fit notes issued by GP practices : December 2014 - March 2017 : experimental statistics. 2017
6. Gabbay MB. Electronic fit notes: sickness certification in the new decade. *Br J Gen Pract* 2010;60(573):235-6. doi: 10.3399/bjgp10X483841
7. NHSDigital. Fit notes issued by GP practices, England. December 2014 - March 2017 2017 [Available from: https://digital.nhs.uk/data-and-information/publications/statistical/fit-notes-issued-by-gp-practices/fit-notes-issued-by-gp-practices-england-december-2014-march-2017.
8. Shiels C, Gabbay MB, Hillage J. Change in diagnosis on fit notes within an episode of certified sickness absence. *Family Practice* 2016;33(5):510-16.
9. Dorrington S, Roberts E, Mykletun A, et al. Systematic review of fit note use for workers in the UK. *Occupational and environmental medicine* 2018 doi: 10.1136/oemed-2017-104730 [published Online First: 2018/05/08]
10. Gabbay M, Shiels C, Hillage J. Factors associated with the length of fit note-certified sickness episodes in the UK. *Occupational and environmental medicine* 2015;72(7):467-75. doi: 10.1136/oemed-2014-102307 [published Online First: 2015/02/26]
11. Dorrington S, Carr E, Stevelink SAM, et al. Demographic variation in fit note receipt and long-term conditions in south London. *Occupational & Environmental Medicine* 2020 doi: 10.1136/oemed-2019-106035 [published Online First: 2020/03/27]
12. Coole C, Potgieter I, Nouri F, et al. Return-to-work outcomes and usefulness of actual fit notes received by employers. *Family Practice* 2015;32(5):551-56.
13. Hussey S, Hoddinott P, Wilson P, et al. Sickness certification system in the United Kingdom: qualitative study of views of general practitioners in Scotland. *BMJ (Clinical research ed)* 2004;328(7431):88. doi: 10.1136/bmj.37949.656389.EE
14. Digital N. Fit Notes Issued by GP Practices 2020 [Available from: https://digital.nhs.uk/data-and-information/publications/statistical/fit-notes-issued-by-gp-practices/guide#csv-metadata accessed April 2020.
15. Rachman R, Bunce D, Thorley K, et al. Patients' attitudes to sickness certification in general practice. *Occupational medicine (Oxford, England)* 2015;65(6):485-8. doi: 10.1093/occmed/kqv069 [published Online First: 2015/06/17]

For peer review only

16. Fletcher-Brown R. 'Mind Your Own Business' - how can we best support employers to improve the mental health of their employees? *Perspect Public Heal* 2015;135(1):18-20.
17. Buist-Bouwman MA, de Graaf R, Vollebergh WA, et al. Comorbidity of physical and mental disorders and the effect on work-loss days. *Acta Psychiatr Scand* 2005;111(6):436-43. doi: 10.1111/j.1600-0447.2005.00513.x [published Online First: 2005/05/10]
18. Dorrington S, Carr E, Stevelink SAM, et al. Multimorbidity and fit note receipt in working-age adults with long-term health conditions. *Psychol Med* 2020:1-10. doi: 10.1017/S0033291720002937 [published Online First: 2020/09/09]
19. Starzmann K, Hjerpe P, Dalemo S, et al. Diagnoses have the greatest impact on variation in sick-leave certification rate among primary-care patients in Sweden: A multilevel analysis including patient, physician and primary health-care centre levels. *Scand J Public Health* 2015;43(7):704-12. doi: 10.1177/1403494815591898 [published Online First: 2015/07/01]
20. Lambeth Council: State of the Borough. Lambeth Council, 2016.
21. Overview of London Boroughs. https://www.trustforlondon.org.uk/data/boroughs/overview-of-london-boroughs/. : Trust for London: Tackling poverty and inequality, 2019.
22. Mixed and balanced communities: Census Information Service, Greater London Authority, London, 2014.
23. Patient Register: quality assurance of administrative data used in population statistics: Office for National Statistics; 2016 [Available from: https://www.ons.gov.uk/peoplepopulationandcommunity/populationandmigration/populationestimates/methodologies/patientregisterqualityassuranceofadministrativedatausedinpopulationstatisticsdec2016.
24. LSOA Atlas. https://data.london.gov.uk/dataset/lsoa-atlas: Greater London Authority, 2014.
25. 2011 Census analysis: Ethnicity and religion of the non-UK born population in England and Wales: 2011. In: Statistics OoN, ed. https://www.ons.gov.uk/peoplepopulationandcommunity/culturalidentity/ethnicity/articles/2011censusanalysisethnicityandreligionofthenonukbornpopulationinenglandandwales/2015-06-18, 2011.
26. Hatch SL, Frissa S, Verdecchia M, et al. Identifying socio-demographic and socioeconomic determinants of health inequalities in a diverse London community: the South East London Community Health (SELCoH) study. *BMC Public Health* 2011;11:861. doi: 10.1186/1471-2458-11-861 [published Online First: 2011/11/15]
27. The English Index of Multiple Deprivation (IMD) 2015. In: Government DfCaL, ed. https://assets.publishing.service.gov.uk/government/uploads/system/uploads/attachment_data/file/464430/English_Index_of_Multiple_Deprivation_2015_-_Guidance.pdf, 2015.
28. Shiels C, Gabbay MB, Hillage J. Change in diagnosis on fit notes within an episode of certified sickness absence. *Fam Pract* 2016;33(5):510-6. doi: 10.1093/fampra/cmw050 [published Online First: 2016/06/28]
29. Shiels C, Gabbay M, Hillage J. Factors associated with prevalence and types of 'may be fit' advice on fit notes: a cross-sectional primary care analysis. *Br J Gen Pract* 2014;64(620):e137-43. doi: 10.3399/bjgp14X677509 [published Online First: 2014/02/26]

For peer review only

30. The King's Fund. Impact of Quality and Outcomes Framework on health inequalities. 2011
31. Economou A, Grey M, McGregor J, et al. The health informatics cohort enhancement project (HICE): using routinely collected primary care data to identify people with a lifetime diagnosis of psychotic disorder. *BMC Research Notes* 2012;5:95. doi: 10.1186/1756-0500-5-95 [published Online First: 2012/02/16]
32. QOF Business Rules - version 35. Department of Health 2017.
33. From One to Many: Exploring people's progression to multiple long-term conditions in an urban environment https://www.gsttcharity.org.uk/what-we-do/our-programmes/multiple-long-term-conditions/one-many-exploring-peoples-progression2018 [
34. Rogers W. Regression standard errors in clustered samples. *Stata Technical Bulletin* 1994;3(13)
35. Shiels CH, J; Pollard E; Gabbay, M. Evaluation of the statement of fitness for work (fit note) : quantitative survey of fit notes. *Research report* 2013(no 841):95 pages.
36. Gabbay M, Shiels C, Hillage J. Sickness certification for common mental disorders and GP return-to-work advice. *Primary Health Care Research Development* 2016:1-11. doi: 10.1017/s1463423616000074 [published Online First: 2016/03/11]
37. Scott KM, Bruffaerts R, Tsang A, et al. Depression-anxiety relationships with chronic physical conditions: results from the World Mental Health Surveys. *Journal of Affective Disorders* 2007;103(1-3):113-20. doi: 10.1016/j.jad.2007.01.015 [published Online First: 2007/02/13]
38. Teesson M, Mitchell PB, Deady M, et al. Affective and anxiety disorders and their relationship with chronic physical conditions in Australia: findings of the 2007 National Survey of Mental Health and Wellbeing. *Australian and New Zealand Journal of Psychiatry* 2011;45(11):939-46. doi: 10.3109/00048674.2011.614590 [published Online First: 2011/10/05]
39. Teesson M, Slade T, Mills K. Comorbidity in Australia: findings of the 2007 National Survey of Mental Health and Wellbeing. *Aust N Z J Psychiatry* 2009;43(7):606-14. doi: 10.1080/00048670902970908 [published Online First: 2009/06/17]
40. Li C, Ford ES, Zhao G, et al. Prevalence and correlates of undiagnosed depression among U.S. adults with diabetes: the Behavioral Risk Factor Surveillance System, 2006. *Diabetes Res Clin Pract* 2009;83(2):268-79. doi: 10.1016/j.diabres.2008.11.006 [published Online First: 2008/12/30]
41. Das-Munshi J, Stewart R, Ismail K, et al. Diabetes, common mental disorders, and disability: findings from the UK National Psychiatric Morbidity Survey. *Psychosomatic Medicine* 2007;69(6):543-50. doi: 10.1097/PSY.0b013e3180cc3062 [published Online First: 2007/07/20]
42. Tsang A, Von Korff M, Lee S, et al. Common chronic pain conditions in developed and developing countries: gender and age differences and comorbidity with depression-anxiety disorders. *J Pain* 2008;9(10):883-91. doi: 10.1016/j.jpain.2008.05.005 [published Online First: 2008/07/08]
43. Moussavi S, Chatterji S, Verdes E, et al. Depression, chronic diseases, and decrements in health: results from the World Health Surveys. *Lancet* 2007;370(9590):851-8. doi: 10.1016/S0140-6736(07)61415-9 [published Online First: 2007/09/11]

For peer review only

44. Evans DL, Charney DS, Lewis L, et al. Mood disorders in the medically ill: scientific review and recommendations. *Biol Psychiatry* 2005;58(3):175-89. doi: 10.1016/j.biopsych.2005.05.001 [published Online First: 2005/08/09]
45. Whooley MA, Caska CM, Hendrickson BE, et al. Depression and inflammation in patients with coronary heart disease: findings from the Heart and Soul Study. *Biol Psychiatry* 2007;62(4):314-20. doi: 10.1016/j.biopsych.2006.10.016 [published Online First: 2007/04/17]
46. Chapman DP, Perry GS, Strine TW. The vital link between chronic disease and depressive disorders. *Prev Chronic Dis* 2005;2(1):A14. [published Online First: 2005/01/27]
47. Woodhead C, Ashworth M, Schofield P, et al. Patterns of physical co-/multi-morbidity among patients with serious mental illness: a London borough-based cross-sectional study. *BMC Family Practice* 2014;15:117. doi: 10.1186/1471-2296-15-117 [published Online First: 2014/06/13]
48. Making more of multimorbidity: an emerging priority. *The Lancet* 2018;391(10131):1637. doi: 10.1016/s0140-6736(18)30941-3
49. Hatch SL, Gazard B, Williams DR, et al. Discrimination and common mental disorder among migrant and ethnic groups: findings from a South East London Community sample. *Soc Psychiatry Psychiatr Epidemiol* 2016 doi: 10.1007/s00127-016-1191-x
50. Hatch SL, Woodhead C, Frissa S, et al. Importance of thinking locally for mental health: data from cross-sectional surveys representing South East London and England. *PLoS One* 2012;7(12):e48012. doi: 10.1371/journal.pone.0048012 [published Online First: 2012/12/20]
51. Labour Force Survey: Sickness absence in the UK labour market 2018 GovUK, 2018.
52. Roche A, Pidd K, Kostadinov V. Alcohol- and drug-related absenteeism: a costly problem. *Aust N Z J Public Health* 2016;40(3):236-8. doi: 10.1111/1753-6405.12414 [published Online First: 2015/08/12]
53. Bockerman P, Hyytinen A, Maczulskij T. Devil in disguise: Does drinking lead to a disability pension? *Prev Med* 2016;86:130-5. doi: 10.1016/j.ypmed.2016.03.008 [published Online First: 2016/03/13]
54. Jones E, Fear NT. Alcohol use and misuse within the military: a review. *Int Rev Psychiatry* 2011;23(2):166-72. doi: 10.3109/09540261.2010.550868 [published Online First: 2011/04/28]
55. Fowlie DG. The misuse of alcohol and other drugs by doctors: a UK report and one region's response. *Alcohol Alcohol* 1999;34(5):666-71. doi: 10.1093/alcalc/34.5.666 [published Online First: 1999/10/21]
56. Flannery J, Ajayi SO, Oyegoke AS. Alcohol and substance misuse in the construction industry. *Int J Occup Saf Ergon* 2019:1-16. doi: 10.1080/10803548.2019.1601376 [published Online First: 2019/04/03]
57. Brooke DE, Griffiths; Taylor, Colin. Addiction as an occupational hazard. *British journal of addiction* 1991
58. Drummond C. Cuts to addiction services are a false economy. *BMJ : British Medical Journal* 2017
59. Roberts E, Hotopf M, Drummond C. The relationship between alcohol-related hospital admission and specialist alcohol treatment provision across local authorities in England since passage of the Health and Social Care Act 2012. *Br J Psychiatry* 2020:1-3. doi: 10.1192/bjp.2020.120 [published Online First: 2020/07/02]

For peer review only

60. Ballard J. TfL's Drug and Alcohol Assessment and Treatment Service. *Occupational Health at Work* 2019;15/5:36-38.
61. Hemmings P. Sickness and disability systems: comparing outomes and policies in Norway with those in Sweden, Netherlands and Switzerland. 2020
62. Byrne P, Ring A, Salmon P, et al. Tussles and Rollovers: Negotiating Sickness Certification in Primary Care. *Advances in Applied Sociology* 2014;Vol.04No.12:14. doi: 10.4236/aasoci.2014.412029

For peer review only

Health condition at first fit note and number of fit notes: evidence from a large primary care sample in south London

Table 1 Number of fit notes received and predicted number of fit notes by sociodemographic variables

Demographic variables	Number of fit notes received: 1st January 2014 to 30th April 2017 n (%)				Predicted NFN unadjusted (95%CI)	Predicted number of fit notes (95% CI)	
	1	2	3	4+		Model 1: Adjusted for age, gender, ethnicity and deprivation	Model 2: +long-term conditions
Whole pop				40,698	2.0 [1.8,2.1]	2.2 [2.1,2.3]	2.4 [2.3,2.5]
Gender							
Male	6,097 (35.2)	3,014 (17.4)	1,878 (10.9)	6,312 (36.5)	2.0 [1.8,2.2]	2.2 [2.1,2.3]	2.4 [2.3,2.6]
Female	8,304 (35.5)	4,231 (18.1)	2,557 (10.9)	8,305 (35.5)	1.9 [1.8,2.1]	2.2 [2.1,2.3]	2.3 [2.2,2.4]
Age group							
16-20	428 (46.9)	178 (19.5)	80 (8.8)	227 (24.9)	1.4 [1.2,1.6]	1.4 [1.2,1.6]	1.7 [1.5,1.9]
21-25	2,054 (49.3)	807 (19.4)	379 (9.1)	926 (22.2)	1.3 [1.2,1.5]	1.4 [1.3,1.5]	1.7 [1.5,2.8]
36-30	2,825 (46.0)	1,138 (18.5)	650 (10.6)	1,532 (24.9)	1.5 [1.4,1.6]	1.6 [1.4,1.7]	1.8 [2.7,2.9]
31-35	2,311 (38.7)	1,136 (19.0)	691 (11.6)	1,839 (30.8)	1.8 [1.7,2.0]	1.9 [2.8,2.0]	2.2 [2.1,2.3]
36-40	1,771 (34.3)	996 (19.3)	591 (11.5)	1,803 (34.9)	2.1 [2.0,2.3]	2.2 [2.0,2.3]	2.4 [2.3,2.5]
41-45	1,541 (31.2)	881 (17.8)	532 (10.8)	1,985 (40.2)	2.4 [2.3,2.6]	2.5 [2.3,2.6]	2.6 [2.4,2.7]
46-50	1,528 (28.1)	880 (16.2)	594 (10.9)	2,437 (44.8)	2.7 [2.5,2.9]	2.7 [2.6,2.9]	2.7 [2.5,2.8]
51-55	1,226 (26.2)	709 (15.1)	536 (11.4)	2,218 (47.3)	2.9 [2.7,3.1]	2.9 [1.8,3.1]	2.8 [2.6,2.9]
56-60	717 (21.9)	520 (15.9)	382 (11.7)	1,650 (50.5)	3.1 [2.9,3.3]	3.2 [3.0,3.4]	2.9 [2.7,3.0]
Ethnicity							
White	8,592 (39.4)	3,979 (18.2)	2,264 (10.4)	6,980 (32.0)	1.8 [1.6,2.0]	2.0 [1.9,2.1]	2.2 [2.1,2.3]
Black African	1,889 (32.4)	1,090 (18.7)	650 (11.1)	2,211 (37.9)	2.1 [2.0,2.3]	2.2 [2.1,2.4]	2.4 [2.2,2.5]
Asian	845 (35.5)	430 (18.1)	292 (12.3)	815 (34.2)	1.9 [1.7,2.2]	2.2 [1.9,2.4]	2.3 [2.1,2.5]
Black Caribbean	1,279 (25.4)	809 (16.1)	567 (11.3)	2,380 (47.3)	2.7 [2.5,2.9]	2.8 [2.7,2.9]	2.9 [2.8,3.0]
Mixed	774 (31.5)	404 (16.5)	300 (12.2)	978 (39.8)	2.2 [2.1,2.4]	2.6 [2.4,2.8]	2.7 [2.6,2.9]
Other	503 (36.2)	268 (19.3)	167 (12.0)	450 (32.4)	1.9 [1.7,2.1]	2.0 [1.8,2.2]	2.3 [2.1,2.5]
Black Other	519 (29.1)	265 (14.9)	195 (10.9)	803 (45.1)	2.6 [2.4,2.8]	2.8 [2.6,3.0]	2.9 [2.7,3.2]
Deprivation							
1	2,325 (39.8)	1,142 (19.5)	626 (10.7)	1,750 (30.0)	1.7 [1.5,1.8]	1.9 [1.8,2.0]	2.0 [2.0,2.2]
2	2,575 (38.1)	1,265 (18.1)	705 (10.4)	2,254 (33.3)	1.8 [1.7,2.0]	2.1 [2.9,2.2]	2.1 [2.1,2.4]
3	2,725 (37.6)	1,411 (17.1)	823 (11.4)	2,836 (33.9)	1.9 [1.7,2.1]	2.1 [2.0,2.3]	2.1 [2.1,2.4]
4	3,067 (32.7)	1,606 (17.8)	1,033 (11.0)	3,521 (38.5)	2.2 [2.0,2.3]	2.3 [2.2,2.5]	2.4 [2.4,2.6]
5	3,353 (32.1)	1,817 (17.1)	1,134 (10.9)	4,195 (39.9)	2.2 [2.1,2.4]	2.4 [2.3,2.6]	2.6 [2.4,2.7]

*mutually adjusted for age, gender, ethnicity and deprivation **mutually adjusted as above, also adjusted for number of long-term conditions (QOF)

For peer review only

Table 2 Predicted number of fit notes by diagnostic group

Category	Groups	Selected sub-groups	N people (%)	Predicted number of fit notes (NFN) [95%CI]	Predicted NFN adj* [95%CI]	Predicted NFN adj** [95%CI]
Mental Health	Total		40,698	2.0 [1.8,2.1]	2.2 [2.1,2.3]	2.4 [2.3,2.5]
	Mental illness***		5,841 (14.4)	2.8 [2.6,3.0]	3.3 [3.1,3.5]	3.3 [3.1,3.4]
		Drug and alcohol	584 (1.4)	3.9 [3.6,4.3]	4.2 [3.8,4.6]	4.5 [4.1,4.8]
		Common Mental Disorders	4,393(10.8)	2.7 [2.5,2.9]	3.2 [3.1,3.4]	3.2 [3.1,3.3]
		Severe Mental Illness	374 (0.9)	2.7 [2.5,3.0]	3.0 [2.8,3.3]	2.6 [2.4,2.8]
	Stress		1,037 (2.6)	2.1 [1.8,2.3]	2.5 [2.2,2.7]	2.7 [2.4,2.9]
	Stress Ext		845 (2.1)	2.2 [1.9,2.4]	2.3 [2.1,2.5]	2.5 [2.3,2.7]
Non Mental Health	MSK		8,366 (20.6)	2.2 [2.1,2.4]	2.3 [2.2,2.5]	2.6 [2.4,2.7]
	Fatigue		541 (1.3)	1.9 [1.6,2.2]	2.2 [2.0,2.5]	2.4 [2.1,2.7]
	Injury		2,183 (5.4)	1.8 [1.6,1.9]	2.1 [1.9,2.2]	2.3 [2.2,2.5]
	Physical health		12,710 (31.2)	2.1 [1.9,2.3]	2.2 [2.1,2.3]	2.3 [2.2,2.4]
	Other		448 (1.1)	2.0 [1.8,2.3]	2.2 [2.0,2.4]	2.3 [2.1,2.5]
	Obstetrics		1,372 (3.4)	1.4 [1.3,1.6]	2.0 [1.8,2.2]	2.2 [2.0,2.4]
	Infection		6,726 (16.5)	1.5 [1.3,1.6]	1.8 [1.7,1.9]	1.9 [1.8,2.0]
	Surgery		629 (1.6)	1.5 [1.3,1.8]	1.6 [1.4,1.9]	1.8 [1.5,2.1]

*adjusted for age, gender, ethnicity and deprivation
**adjusted for age, gender, ethnicity, deprivation and number of long-term conditions
***made up of drug & alcohol, common mental disorders, severe mental illness, mental health treatment and mental health other

For peer review only

For peer review only

Figure 1

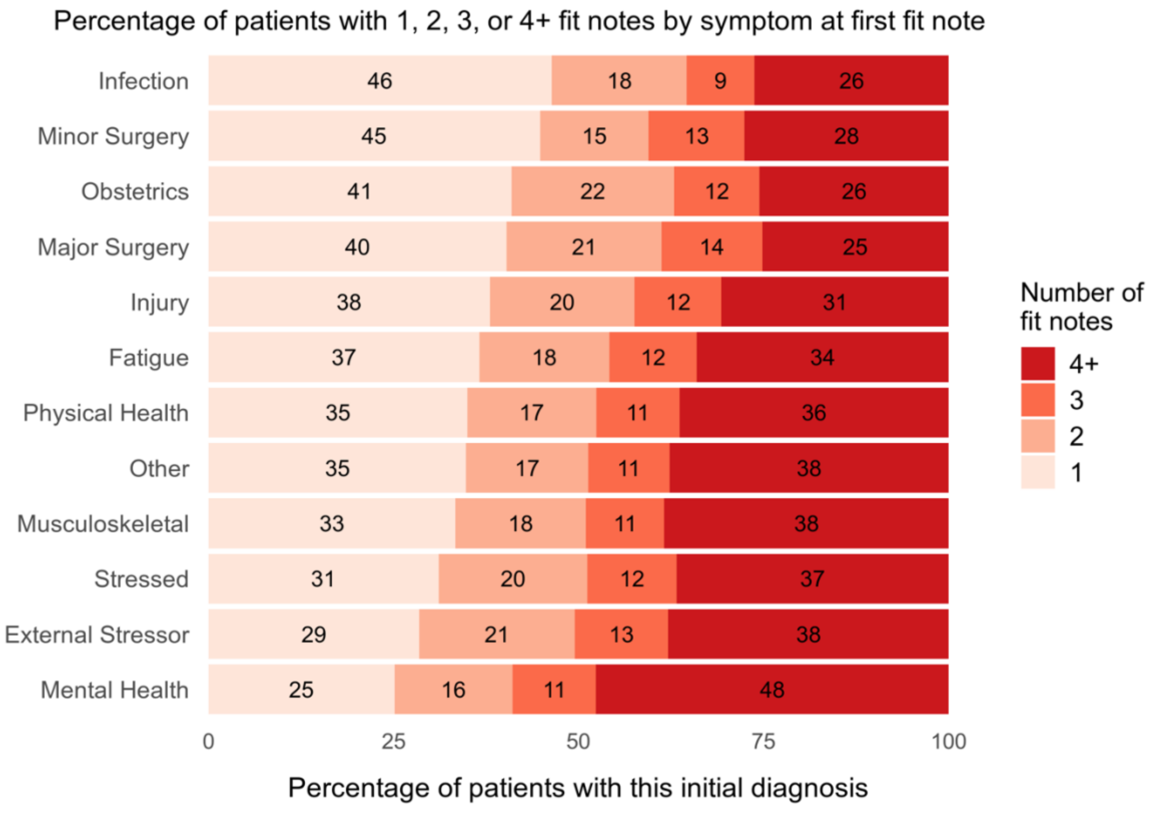

Data source: Lambeth Datanet. Sample size: 40698 patients with fit notes aged 16-60, registered with a Lambeth GP between 1st Jan 2014 and 30th April 2017.

For peer review only - http://bmjopen.bmj.com/site/about/guidelines.xhtml

For peer review only

SUPPLEMENTARY MATERIAL TABLES

Table 1 Predicted number of fit notes by condition type

Condition sub-group	Number (%)	Predicted number of fit notes (NFN)	Predicted NFN adj*	Predicted NFN adj**
Drug and alcohol	584 (1.4)	3.9 (3.6-4.3)	4.2 (3.8-4.6)	4.5 (4.1-4.8)
Other Mental Health Problem	294 (0.7)	2.7 (2.3-3.0)	3.7 (3.2-4.2)	3.6 (3.1-4.0)
Common Mental Disorders	4,393(10.8)	2.7 (2.5-2.9)	3.2 (3.1-3.4)	3.2 (3.1-3.3)
Severe Mental Illness	374 (0.9)	2.7 (2.5-3.0)	3.0 (2.8-3.3)	2.6 (2.4-2.8)
Neurology	640 (1.6)	2.5 (2.2-2.8)	2.8 (2.5-3.1)	2.8 (2.5-3.1)
Haematology	134 (0.3)	2.5 (2.1-2.9)	2.8 (2.3-3.3)	3.2 (2.6-3.8)
Obesity	74 (0.2)	2.7 (2.0-3.3)	2.7 (2.0-3.4)	2.4 (1.8-2.9)
Insomnia	174 (0.4)	2.2 (1.8-2.7)	2.6 (2.0-3.2)	2.7 (2.1-3.3)
Mental Health Treatment	192 (0.5)	2.3 (1.7-2.8)	2.6 (2.0-3.1)	2.4 (1.9-2.9)
Cancer	239 (0.6)	2.5 (2.3-2.8)	2.5 (2.2-2.7)	2.5 (2.2-2.7)
Stressed	1,039 (2.6)	2.1 (1.9-2.3)	2.5 (2.2-2.7)	2.7 (2.5-2.9)
Diabetes	58 (0.1)	2.8 (2.0-3.6)	2.5 (1.8-3.2)	2.1 (1.5-2.7)
Systemic Illness	1,028 (2.5)	2.6 (2.3-2.8)	2.4 (2.2-2.6)	2.1 (1.9-2.3)
Physical symptom	1,439 (3.5)	2.2 (2.0-2.4)	2.4 (2.2-2.5)	2.5 (2.3-2.6)
Other	326 (0.8)	2.2 (1.9-2.5)	2.4 (2.1-2.6)	2.5 (2.2-2.7)
External stressor	349 (0.9)	2.1 (1.8-2.5)	2.4 (2.0-2.7)	2.6 (2.2-2.9)
Musculoskeletal	8,288 (20.4)	2.3 (2.1 2.4)	2.3 (2.2-2.5)	2.6 (2.4-2.7)
Bereavement	495 (1.2)	2.2 (1.9-2.5)	2.3 (2.1-2.6)	2.5 (2.3-2.8)
Test	26 (0.1)	2.7 (1.7-3.7)	2.3 (1.6-3.1)	2.3 (1.6-3.0)
Cardiovascular	1,234 (3.0)	2.5 (2.2-2.7)	2.2 (2.1-2.4)	2.2 (2.0-2.3)
Gastroenterology	2,241 (5.5)	2.0 (1.8-2.1)	2.2 (2.1-2.4)	2.4 (2.3-2.6)
Gynaecology	768 (1.9)	1.9 (1.7-2.0)	2.2 (2.0-2.3)	2.4 (2.3-2.6)
Dermatology	1,129 (2.8)	1.9 (1.6-2.1)	2.2 (1.9-2.4)	2.3 (2.1-2.6)
Respiratory	1,663 (4.1)	2.0 (1.8-2.2)	2.1 (2.0-2.3)	2.0 (1.9-2.2)
Injury	2,183 (5.4)	1.8 (1.6-2.0)	2.1 (1.9-2.3)	2.4 (2.2-2.5)
Fatigue	367 (0.9)	1.8 (1.5-2.1)	2.1 (1.8-2.4)	2.3 (2.0-2.6)
Physical Health Treatment	13 (0.0)	1.9 (1.0-2.9)	2.1 (1.0-3.2)	2.1 (0.8-3.3)
Genitourinary Medicine/Urology	481 (1.2)	2.0 (1.7-2.3)	2.0 (1.8-2.3)	2.1 (1.9-2.4)
Obstetrics	1,371 (3.4)	1.4 (1.3-1.6)	2.0 (1.8-2.2)	2.2 (2.0-2.5)
Ear, Nose, Throat and Dental	1,016 (2.5)	1.8 (1.6-2.1)	2.0 (1.7-2.2)	2.1 (1.9-2.4)
Wound	562 (1.4)	1.8 (1.6-2.0)	1.9 (1.8-2.1)	2.1 (1.9-2.3)
Infection	6,727 (16.5)	1.5 (1.4-1.7)	1.8 (1.7-1.9)	1.9 (1.8-2.0)
Risk factor	33 (0.1)	1.8 (1.1-2.5)	1.8 (1.2-2.3)	1.8 (1.2-2.4)
Minor Surgery	232 (0.6)	1.6 (1.3-1.9)	1.7 (1.4-2.0)	1.9 (1.5-2.3)
Allergy	88 (0.2)	1.4 (1.0-1.9)	1.7 (1.2-2.2)	1.9 (1.3-2.4)
Major Surgery	397 (1.0)	1.6 (1.3-1.8)	1.6 (1.4-1.9)	1.8 (1.5-2.0)
Renal	47 (0.1)	1.6 (0.8-2.3)	1.6 (0.9-2.4)	1.9 (1.0-2.7)

*adjusted for age, gender, ethnicity and deprivation

**adjusted for age, gender, ethnicity, deprivation and number of long term condition

For peer review only

Table 2 Example of subgroup codes:

Drug and Alcohol Codes:
[RFC] Drug/ substance abuse<br>[RFC] Substance abuse<br>[X]Alcohol addiction<br>[X]Drug addiction - cannabis<br>[X]Heroin addiction<br>[X]Men & behav disorder multiple drug use/psychoactive subst<br>[X]Mental and behav dis due to use alcohol: dependence syndr<br>[X]Mental and behav dis due to use cannabinoids: harmful use<br>[X]Mental and behav dis due to use of alcohol: harmful use<br>[X]Mental and behav dis due to use of cocaine: harmful use<br>[X]Mental and behav dis due to use of opioids: harmful use<br>[X]Mental and behav dis due to use opioids: dependence syndr<br>[X]Mental behav disorders due use crack cocaine: harmful use<br>Alcohol dependence syndrome<br>Alcohol dependence syndrome NOS<br>Alcohol misuse - enhanced service completed<br>Alcohol problem drinking<br>Alcohol withdrawal syndrome<br>Alcoholism<br>Analgesic overuse headache<br>Benzodiazepine dependence<br>Cannabis dependence, continuous<br>Cannabis dependence, unspecified<br>Cannabis type drug dependence<br>Chronic alcoholism<br>Chronic alcoholism in remission<br>Cocaine dependence, episodic<br>Cocaine dependence, unspecified<br>Cocaine drug dependence NOS<br>Cocaine type drug dependence<br>Diazepam dependence<br>Drug addiction<br>Drug addiction therapy<br>Drug addictn therap-methadone<br>Drug dependence<br>Drug dependence NOS<br>Drug dependence therapy<br>Drug seeking behaviour<br>Drug withdrawal syndrome<br>Drunkenness NOS<br>Hangover (alcohol)<br>Harmful alcohol use<br>Hazardous alcohol use<br>Heavy drinker - 7-9u/day<br>Heroin dependence<br>Intoxication - alcohol<br>Methadone dependence<br>Misuse of drugs NOS<br>Morphine dependence<br>Nondependent alcohol abuse<br>Nondependent cannabis abuse<br>Opioid drug dependence NOS<br>Opioid type drug dependence<br>Refer to drugs service<br>Very heavy drinker - >9u/day

For peer review only

Supplementary Figure 1 Classification of conditions into sub-groups, groups and categories

**Categories**	**Groups**	**Sub-groups**
Non-Mental Health	Injury*	Injury*
	Infection*	Infection*
	Surgery*	Minor Surgery*<br>Major Surgery*
	Fatigue/Insomnia	Insomnia<br>Fatigue
	Obstetrics	Obstetrics
	Musculoskeletal	Musculoskeletal
	Physical Health	Cancer<br>Neurology<br>Haematology<br>Obesity<br>Test<br>Cardiovascular<br>Gastroenterology<br>Gynaecology<br>Dermatology<br>Respiratory<br>Physical Health Treatment<br>Genitourinary Medicine/Urology<br>Allergy<br>Risk factor<br>Ear, Nose, Throat and Dental<br>Renal<br>Diabetes<br>Systemic Illness<br>Physical symptom<br>Wound
	Other	Other Physical Health
Mental Health	Mental Illness	Common Mental Disorders<br>Severe Mental Illness<br>Drug and Alcohol<br>Mental Health Treatment<br>Other Mental Health Problem**
	External Stressor	Bereavement<br>External stressor
	Stressed	Stressed

*Injury, infection and surgery were at the top of the two-tier hierarchy of groupings, as an alternative to assigning injuries, infections and surgery to their system groups.
** Other mental health problem includes self-harm, developmental disorders, personality disorder

Supplementary Figure 2: Percentage of patients with 1, 2, 3 or 4+ fit notes by symptom at first fit note

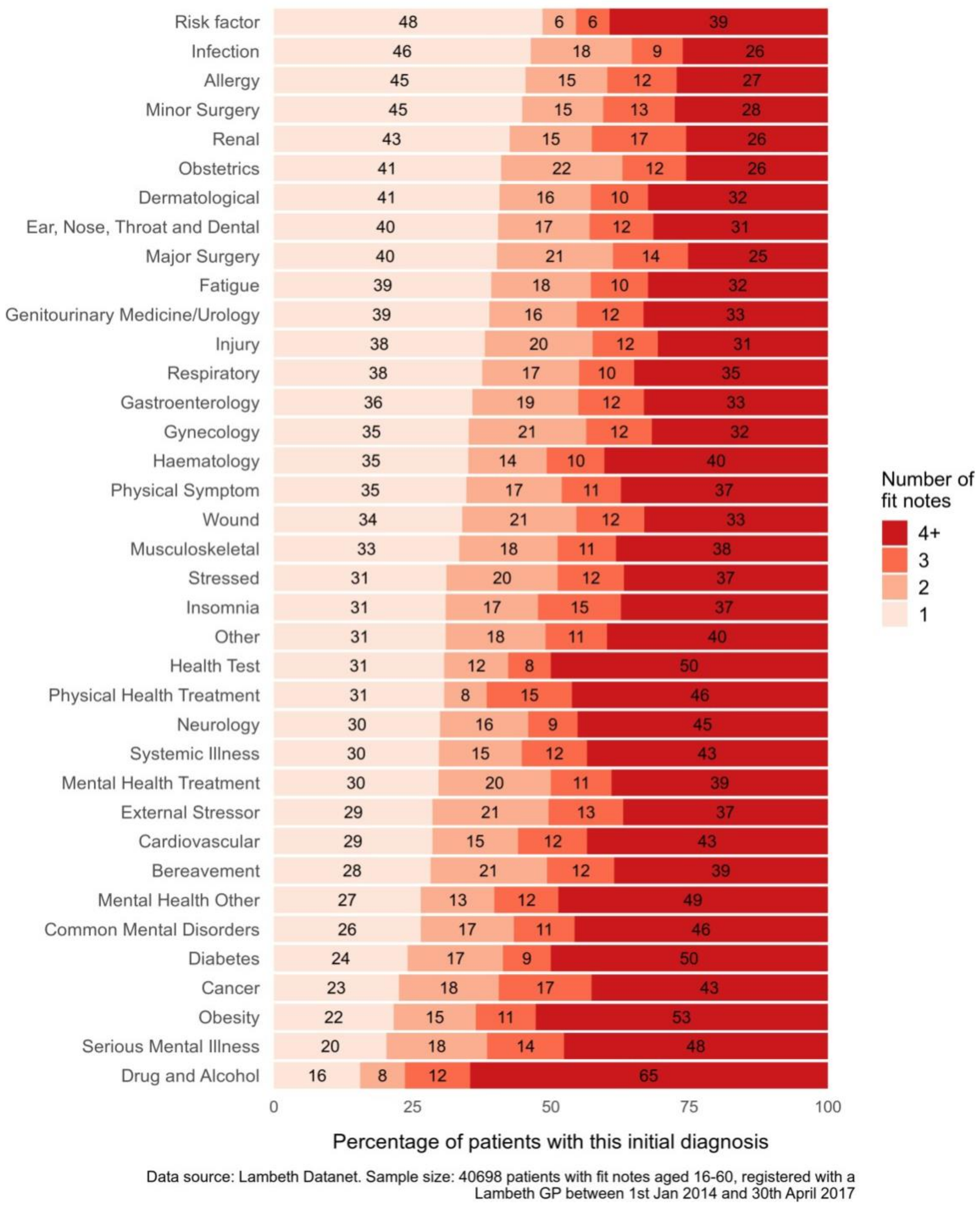

Reporting checklist for cohort study.

Based on the STROBE cohort guidelines.

Instructions to authors

Complete this checklist by entering the page numbers from your manuscript where readers will find each of the items listed below.

Your article may not currently address all the items on the checklist. Please modify your text to include the missing information. If you are certain that an item does not apply, please write "n/a" and provide a short explanation.

Upload your completed checklist as an extra file when you submit to a journal.

In your methods section, say that you used the STROBE cohortreporting guidelines, and cite them as:

von Elm E, Altman DG, Egger M, Pocock SJ, Gotzsche PC, Vandenbroucke JP. The Strengthening the Reporting of Observational Studies in Epidemiology (STROBE) Statement: guidelines for reporting observational studies.

For peer review only

		Reporting Item	Page Number
**Title and abstract**			
Title	#1a	Indicate the study’s design with a commonly used term in the title or the abstract	1
Abstract	#1b	Provide in the abstract an informative and balanced summary of what was done and what was found	3
**Introduction**			
Background / rationale	#2	Explain the scientific background and rationale for the investigation being reported	4
Objectives	#3	State specific objectives, including any prespecified hypotheses	4
**Methods**			
Study design	#4	Present key elements of study design early in the paper	5
Setting	#5	Describe the setting, locations, and relevant dates, including periods of	5

		recruitment, exposure, follow-up, and data collection	
Eligibility criteria	#6a	Give the eligibility criteria, and the sources and methods of selection of participants. Describe methods of follow-up.	6
Eligibility criteria	#6b	For matched studies, give matching criteria and number of exposed and unexposed	n/a
Variables	#7	Clearly define all outcomes, exposures, predictors, potential confounders, and effect modifiers. Give diagnostic criteria, if applicable	6 and 7
Data sources / measurement	#8	For each variable of interest give sources of data and details of methods of assessment (measurement). Describe comparability of assessment methods if there is more than one group. Give information separately for for exposed and unexposed groups if applicable.	6 and 7
Bias	#9	Describe any efforts to address potential sources of bias	11
Study size	#10	Explain how the study size was arrived at	7
Quantitative variables	#11	Explain how quantitative variables were handled in the analyses. If applicable, describe which groupings were chosen, and why	6
Statistical methods	#12a	Describe all statistical methods, including those used to control for confounding	7
Statistical methods	#12b	Describe any methods used to examine subgroups and interactions	7
Statistical methods	#12c	Explain how missing data were addressed	7
Statistical methods	#12d	If applicable, explain how loss to follow-up was addressed	n/a
Statistical methods	#12e	Describe any sensitivity analyses	n/a
**Results**			
Participants	#13a	Report numbers of individuals at each stage of study—eg numbers potentially eligible, examined for eligibility, confirmed eligible, included in the study, completing follow-up, and analysed. Give information separately for for exposed and unexposed groups if applicable.	7

For peer review only

Participants	#13b	Give reasons for non-participation at each stage	7
Participants	#13c	Consider use of a flow diagram	n/a
Descriptive data	#14a	Give characteristics of study participants (eg demographic, clinical, social) and information on exposures and potential confounders. Give information separately for exposed and unexposed groups if applicable.	7
Descriptive data	#14b	Indicate number of participants with missing data for each variable of interest	7
Descriptive data	#14c	Summarise follow-up time (eg, average and total amount)	na
Outcome data	#15	Report numbers of outcome events or summary measures over time. Give information separately for exposed and unexposed groups if applicable.	7 and 8
Main results	#16a	Give unadjusted estimates and, if applicable, confounder-adjusted estimates and their precision (eg, 95% confidence interval). Make clear which confounders were adjusted for and why they were included	7
Main results	#16b	Report category boundaries when continuous variables were categorized	7
Main results	#16c	If relevant, consider translating estimates of relative risk into absolute risk for a meaningful time period	na
Other analyses	#17	Report other analyses done—e.g., analyses of subgroups and interactions, and sensitivity analyses	9
**Discussion**			
Key results	#18	Summarise key results with reference to study objectives	9
Limitations	#19	Discuss limitations of the study, taking into account sources of potential bias or imprecision. Discuss both direction and magnitude of any potential bias.	11
Interpretation	#20	Give a cautious overall interpretation considering objectives, limitations, multiplicity of analyses, results from similar studies, and other relevant evidence.	11
Generalisability	#21	Discuss the generalisability (external validity) of the study results	11
**Other Information**			

For peer review only

Funding	#22	Give the source of funding and the role of the funders for the present study and, if applicable, for the original study on which the present article is based	1

The STROBE checklist is distributed under the terms of the Creative Commons Attribution License CC-BY. This checklist was completed on 16. August 2020 using https://www.goodreports.org/, a tool made by the EQUATOR Network in collaboration with Penelope.ai

For peer review only
